# The gut microbiota of three avian species living in sympatry

**DOI:** 10.1186/s12862-024-02329-9

**Published:** 2024-11-21

**Authors:** Hugo Pereira, Nayden Chakarov, Barbara A. Caspers, Marc Gilles, William Jones, Tafitasoa Mijoro, Sama Zefania, Tamás Székely, Oliver Krüger, Joseph I. Hoffman

**Affiliations:** 1 Department of Animal Behaviour, Bielefeld University, Konsequenz 45, Bielefeld, 33615 Germany; 2 Department of Evolutionary Population Genetics, Bielefeld University, Konsequenz 45, Bielefeld, 33615 Germany; 3 Department of Behavioural Ecology, Bielefeld University, Konsequenz 45, Bielefeld, 33615 Germany; 4 Institut Supérieur de Technologie de Menabe, Université of Toliara & Morondava, Toliara, 601 Madagascar; 5 HUN-REN-Debrecen University Reproductive Strategies Research Group, University of Debrecen, Egyetem tér 1, Debrecen, H-4032 Hungary; 6 Joint Institute for Individualisation in a Changing Environment (JICE), Bielefeld University and University of Münster, Konsequenz 45, Bielefeld, 33615 Germany; 7 British Antarctic Survey, High Cross, Madingley Road, Cambridge, CB3 OET UK; 8 Center for Biotechnology (CeBiTec), Faculty of Biology, Bielefeld University, Universitätsstraße 25, Bielefeld, 33615 Germany; 9 Milner Centre for Evolution, Department of Life Sciences, University of Bath, Claverton Down, Bath, BA2 7AY UK

**Keywords:** Gut microbiota, Genetics, Holobiont, Endemic, Madagascar, Plovers

## Abstract

**Background:**

Evolutionary divergence and genetic variation are often linked to differences in microbial community structure and diversity. While environmental factors and diet heavily influence gut microbial communities, host species contributions are harder to quantify. Closely related species living in sympatry provide a unique opportunity to investigate species differences without the confounding effects of habitat and dietary variation. We therefore compared and contrasted the gut microbiota of three sympatric plover species: the widespread Kittlitz’s and white-fronted plovers (*Anarhynchus pecuarius* and *A. marginatus*) and the endemic and vulnerable Madagascar plover (*A. thoracicus*).

**Results:**

We found no significant differences in the beta diversity (composition) of the gut microbiota of the three species. However, *A. thoracicus* exhibited higher intraspecific compositional similarity (i.e. lower pairwise distances) than the other two species; this pattern was especially pronounced among juveniles. By contrast, microbial alpha diversity varied significantly among the species, being highest in *A. pecuarius*, intermediate in *A. marginatus* and lowest in *A. thoracicus*. This pattern was again stronger among juveniles. Geographical distance did not significantly affect the composition of the gut microbiota, but genetic relatedness did.

**Conclusion:**

While patterns of microbial diversity varied across species, the lack of compositional differences suggests that habitat and diet likely exert a strong influence on the gut microbiota of plovers. This may be enhanced by their precocial, ground-dwelling nature, which could facilitate the horizontal transmission of microbes from the environment. We hypothesise that gut microbiota diversity in plovers primarily reflects the ecological pool of microbiota, which is subsequently modified by host-specific factors including genetics. The reduced microbial and genetic diversity of the endemic *A. thoracicus* may hinder its ability to adapt to environmental changes, highlighting the need for increased conservation efforts for this vulnerable species.

**Supplementary Information:**

The online version contains supplementary material available at 10.1186/s12862-024-02329-9.

## Introduction

All living organisms host a community of microorganisms referred to as the microbiome, with the highest abundance of these organisms located in the gut, collectively known as the gut microbiota [1]. Rosenberg and Zilber-Rosenberg [2] proposed that organisms should be defined along with their microbiota as holobionts. They argued that both the host and its microbial communities are subject to the same evolutionary forces and research should focus on the combined genomic content of the host and microbiota, referred to as the hologenome.

In avian species, gut microbial colonisation commences shortly after hatching, with embryos developing within a closed and essentially sterile environment, the egg [3]. The chick gastrointestinal tract is initially colonised by various transient taxa, with bacterial communities gradually shifting to a stable adult state [4–6]. Numerous studies have demonstrated the importance of gut microbial communities in regulating physiological functions such as digestion, absorption, metabolism, and immune responses, impacting host health across diverse animal species [7]. Meanwhile, the gut microbiota is in turn influenced by factors such as host genetics, environment, diet, immune function, and behaviour [5, 7, 8]. While environmental and dietary factors are recognised as primary drivers of microbiota variation, the specific contributions of each factor remains unclear [9–11].

Host genetics often plays a dominant role in shaping gut microbiota composition in mammals, including humans [12, 13]. However, diet has also been identified as a significant factor [14, 15]. When host-microbe associations persist over the long term, gut microbes tend to show species-specific differences that are shaped by the evolutionary divergence of their hosts [16]. However, birds tend to show less pronounced interspecific differences in their gut microbiota than mammals [17]. One hypothesis posits that differences between birds and non-flying mammals stem from the fact that birds evolved a simpler gastrointestinal tract with significantly decreased gut retention times as an adaptation to flight [17]. These reduced retention times plus a simpler gut environment may promote rapid turnover of the avian gut microbiota, accentuating the influence of diet and environment over host taxonomy in shaping the gut microbiota [17]. For example, species-level differences were identified in the gut microbiota of 37 New Guinean passerine species [18] while in Darwin’s finches, gut microbiota communities tend to cluster more by host habitat than by host species [19]. Similarly, captive birds often have distinct gut microbiota compared to their wild counterparts, likely due to manipulated diets, artificial habitats and interactions with humans [20]. Furthermore, a study of two nightingale species found no significant differences in the gut microbiota of sympatric and allopatric populations, with most of the observed variation being explained by inter-individual differences [21].

The profound influence of microbial interactions on host biology, including adaptation to environmental changes, and the holobiont concept, suggest that hosts function not in isolation but as interconnected networks comprising the host and its associated microbiota [2, 22, 23]. This raises questions about how the gut microbiota may react to population declines and anthropogenic environmental changes. Endangered animal populations facing declines often suffer from inbreeding depression and the loss of genetic diversity through drift, which can impair their adaptive capacity and increase extinction risk [24–27]. Recent research, framed within the hologenome concept, suggests that the gut microbiota can also be affected by these factors, leading to microbial disruptions that can impede host fitness and reduce adaptive capacity [28–30]. Hence, the role of the gut microbiota in the health of endangered wildlife is of increasing concern, especially in the face of climate change and other anthropogenic pressures [31, 32].

This study aims to elucidate the role of host species in shaping the gut microbiota of three plover species of the same genus breeding in sympatry. We leverage the “natural laboratory” provided by the island of Madagascar to investigate the impact of host species occupying similar ecological niches [33]. Shorebirds of the genus *Anarhynchus* (formerly embedded within *Charadrius*) offer a highly tractable system within Madagascar [34]. In the southwestern part of the island, the Madagascar endemic and endangered *A. thoracicus* (Madagascar plover) breeds in sympatry with two more widely distributed African species: *A. pecuarius* (Kittlitz’s plover) and *A. marginatus* (White-fronted plover) [33]. These three plovers are sister species [35], with a recent study placing the divergence time between *A. pecuarius* and *A. thoracicus* at approximately three million years ago (Mya) and the split between *A. marginatus* and the *A. pecuarius*/*A. thoracicus* clade at about eight Mya [36].

**Fig. 1:**
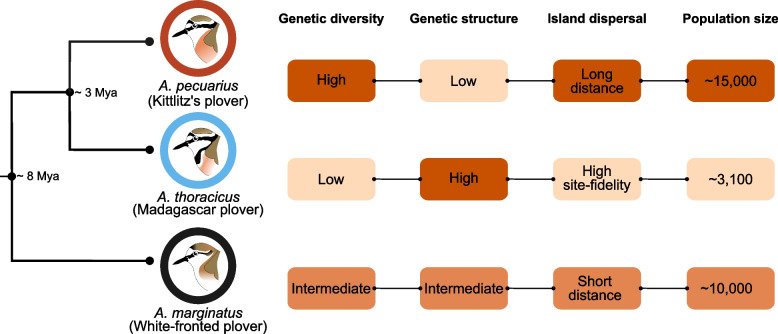
Evolutionary divergence and a summary of the key differences between the three plover species [33, 36, 41]

*A. pecuarius* is the most abundant of the three plover species studied on Madagascar, with an estimated population size of approximately 10,000 to 20,000 individuals. It is widely distributed and has a high dispersal capacity, inhabiting salt marshes in coastal areas as well as wet grasslands and riverbanks further inland [33, 37]. *A. marginatus* has an estimated population size of 5,000 to 15,000 individuals [33, 37]. While also widespread, it primarily inhabits coastal environments, particularly open sandy beaches and salt marshes, and its dispersal ability is not as high as that of *A. pecuarius* [33, 37]. The endemic *A. thoracicus* has the smallest population, estimated at around 3,500 individuals [38]. It is a site specialist, residing in sparsely vegetated shorelines of lakes and salt marshes within 10 km of the west coast [33, 38, 39]. Due to its low abundance, limited habitat and increasing habitat alteration due to human activities, this species is considered vulnerable [40]. A comparative population genetic study found that *A. pecuarius* exhibits high genetic diversity but no population structure, while *A. marginatus* shows intermediate levels of both. By contrast, *A. thoracicus* has the lowest genetic diversity and exhibits strong population structure [41] (Fig. 1 summarises the key characteristics of the three plover species).

We hypothesised that if evolutionary divergence plays a significant role in shaping the composition of the gut microbiota, the three plover species should have compositionally distinct gut microbiota. Moroever, we expected that the two closely related species, *A. pecuarius* and *A. thoracicus*, would carry more similar gut microbial communities compared to the more distantly related *A. marginatus*. We further hypothesised that the Madagascar plover would have the lowest microbiota diversity due to its endemic status, site specialism, small population size and low genetic diversity. By contrast, the abundant and widespread *A. pecuarius* should exhibit the highest microbial diversity, with *A. marginatus* showing intermediate diversity. This study system allows for comparisons between species in sympatry, providing insights into the microbiota from a conservation perspective.

## Methods

### Study area and sample collection

The study was conducted during the breeding seasons of 2021 and 2022 in the vicinity of Andavadoaka, a fishing village located in southwestern Madagascar (S$^{\circ }$22.02, E$^{\circ }$43.39). This area is characterised by a landscape composed by sandy beaches, salt marshes, and temporary saltwater lagoons, all surrounded by dry, spiny forest [42]. Breeding activity follows seasonal heavy rainfall, typically starting as early as December and January and concluding by early June, coinciding with the drying of floodwaters [33]. In the study area, the three plover species breed in sympatry, with sampling conducted primarily within saltwater marshes [33]. Furthermore, long-term observational data from the site suggests that there are no significant dietary differences among the three species.

Adult individuals were trapped using funnel traps or spring traps positioned on top of the nests [43]. Each captured individual was uniquely marked with a combination of Darvic colour rings and alphanumeric SAFRING metal rings. Nest locations were recorded using GPS devices (Garmin Map 64x) and data on egg sizes and clutch sizes were collected. Chicks were carefully captured by hand, following observation and careful approach. Families were identified by continuous observation. Morphological characteristics such as body mass, tarsus length, wing length, and bill length were measured according to established protocols [34]. In addition to standard biometrics, and given the lack of sexual dimorphism of the three species [44], blood samples were collected from all captured individuals for molecular sexing. Blood samples (25-50 $\upmu$l) were obtained via brachial venepuncture and preserved in 96% ethanol [34]. Sex determination followed the standard protocol described by Fridolfsson and Ellegren [45]. For gut microbiota analysis, faecal samples were collected according to the procedure outlined by Knutie and Gotanda [46]. Individuals were temporarily placed in a paper bag containing a sterile tray with a wire grate on top in order to prevent contact with the faecal matter. Individuals remained within the bag for approximately 2-3 min until defecation occurred (if no defecation occurred within this period, the bird was released). The individuals were subsequently released and the faecal samples were transferred into 96% ethanol collection tubes. The tray and wire grate were then sterilised using a 10% bleach solution and 96% ethanol. Environmental controls were acquired by swabbing diverse surfaces around the field working station. Blank swabs were also collected to control for the risk of contamination throughout the sampling procedure.

### Faecal DNA isolation and sequencing

Ethanol-preserved faecal samples were air dried prior to DNA extraction. Microbial DNA extraction was performed using the QIAamp PowerFecal Pro DNA Kit (Qiagen) in accordance with the manufacturer’s instructions, with minor adaptations. Following the addition of solution CD1, the samples were incubated at 65$^{\circ }$C for 10 min, and an additional digestion step with Proteinase K was added (2 h at 56$^{\circ }$C) following mechanical lysis. Microbial DNA extracts were subsequently stored at -80$^{\circ }$C until further analysis.

The amplification and sequencing of the 16 S rRNA gene was outsourced to Biomarker Technologies (BMKGENE) GmbH. The V3-V4 region of the 16 S rRNA gene was targeted utilising the primer set 338F/806R [47], the pooled libraries were then sequenced on an Illumina Novaseq 6000 platform (1% of the run) employing a 2x250 bp paired-end reads protocol. In addition to 202 biological samples, the final library pool also included the $ZymoBIOMICS^{TM}$ Microbial Community DNA Standard (D6305), four environmental controls and two extraction blanks. Negative controls were included to monitor potential contamination throughout the entire procedure, while positive controls served for quality control analysis.

### Bioinformatics analysis

Illumina sequence data were imported into QIIME2 (Quantitative Insights Into Microbial Ecology 2), version 2022.11 [48]. Quality assessment of reads was conducted by visualising quality plots. The Divisive Amplicon Denoising Algorithm pipeline (DADA2) was employed to filter out low quality bases and infer Amplicon Sequencing Variants (ASVs) [49]. Forward and reverse sequences were truncated at 245 and 242 base pairs, respectively, with 20 base pairs trimmed from the 5’ end of the reads. Taxonomy was assigned to the ASVs using a naive Bayes taxonomic classifier trained on the SILVA SSU 138.1 database [50]. The classifier was built and trained using the REference Sequence annotation and CuRatIon Pipeline plugin (RESCRIPt) [51]. The processed data were imported into R version 4.2.2 [52] using the qiime2R package version 0.99.6 [53]. Sequence contaminants were identified and removed using the decontam package version 1.18 [54]. The “prevalence” method, with a probability threshold of 0.1, was applied for contaminant removal. This method compares the prevalence of each sequence feature present in true samples to the prevalence in negative controls. ASVs assigned to Mitochondria, Chloroplast, *Vertebrata*, *Eukaryota*, and unassigned taxa were filtered out, and singletons were removed. Samples containing more than 2000 reads were retained for further analysis. Prevalence and abundance-based filtering was performed, retaining ASVs with an abundance of at least 0.01% in at least 10% of the samples.

To assess the pipeline’s performance, the q2-quality-control plugin was utilised to evaluate the accuracy of taxonomic composition reconstruction against community standards. Quality control results identified 37 false positives (results from microbial community standard analysis are presented in Appendix B); these ASVs were subsequently removed from the data using the R phyloseq package [55]. Using the q2-phylogeny plugin, we then aligned the remaining 28,278 ASVs (Appendix A Table S1) using MAFFT [56] and constructed a phylogeny using FastTree [57]. In order to assess sequencing depth and sample coverage, rarefaction curves were generated using the q2-diversity-alpha-rarefaction plugin (Appendix A Fig. S1). Taxa bar plots were generated exclusively for core taxa, defined as those taxa common to all three species with a minimum prevalence of 95% across individuals. Supplementary tables and figure can be found in Appendix A while detailed scripts for the bioinformatics analyses can be found in Appendix C.

### Statistical analysis

Assessment of gut microbiota differences among the three plover species was implemented as follows: first, we analysed the combined dataset, including both juveniles (chicks) and adults; subsequently, we analysed juveniles and adults separately to determine whether differences/similarities emerged at distinct developmental stages.

#### Microbial composition (beta diversity)

Microbial abundances at the phylum and family levels across different species and age groups were depicted through stacked-bar plots generated using ggplot2 v. 3.4.2 [58]. Additionally, to visualise shared and unique ASVs among species, two Venn diagrams were constructed: one illustrating the raw number of ASVs and the other weighted by relative abundance. The diagrams were created using the MiEco version 0.19.19 R package [59].

Between-species differences were estimated using the unrarified dataset and subjected to Cumulative Sum Scaling (CSS) normalisation [60] with the R package metagenomeseq version 1.30.0 [61]. Differences in composition were inferred based on Bray-Curtis dissimilarities (BC) [22] and weighted UniFrac distances (WU) [62]. Principal Coordinate Analysis (PCoA) was employed to visualise the results using the “ordinate” function implemented in the vegan package version 2.6-4 [63]. Significant associations between beta-diversity metrics and variables of interest were assessed using PERMANOVA (10000 permutations) with the “adonis2” function from the vegan package [63]. The model was built with BC and WU as response variables; and species, age (only for analysis of the combined dataset), sex and year as fixed effects. Nest ID was used as a blocking factor to control for the non-independence of samples. Models were fitted using the “margin” option, which allowed us to test for the marginal effect of each variable while accounting for the other variables in the model. Homogeneity of group dispersion was tested for using the “permutest” function in vegan.

To further investigate species differences, a Bayesian framework was adopted to model pairwise (dyadic) values, as described by Raulo et al. [64]. Bayesian regression models were fitted using the brms package [65]. The models incorporated pairwise comparisons between individuals, with BC and WU fitted as response variables. Fixed effects included matrices of species combination, sex combination, age combination (only for the combined dataset including both adults and juveniles), nest sharing, and year (coded as 0/1 for different/same). To address data dependency resulting from pairwise comparisons, a multi-membership random effect [66] capturing the individuals in each dyad (ID A - ID B) was included in the model. To test for associations between spatial distances among individuals and microbiota beta diversity, a Mantel test [67] (vegan package) was performed. This involved comparing a matrix of geographical distance in metres between each individual’s capture location with the BC and WU matrices. The Mantel test was run with 9,999 permutations and controlled for the relatedness between individuals (Nest ID). Complete scripts are shown in Appendix D.

#### Microbial diversity (alpha diversity)

In QIIME2, sequencing depth and sample coverage were assessed through rarefaction plots, revealing a plateau at approximately 20,000 reads (Appendix A Fig. S1). Subsequently, the dataset was rarefied to the sample with the fewest reads (27,378). Three metrics of alpha diversity - the Shannon diversity index [68], Faith’s Phylogenetic Diversity (Faith PD) [69], and the number of observed ASVs - were computed using the q2-diversity-alpha plugin.

To investigate species differences in gut microbiota diversity, linear mixed models (LMMs) with a Gaussian distribution were computed using the “lmer” function from the lme4 package in R [70]. To account for differences in sampling size between the three species, models were run with a bootstrapping procedure using the lmeresampler package [71] with 10,000 iterations. The significance of model estimates was assessed through analysis of 95% confidence intervals (CIs). A variable was considered to be significantly associated with microbiota diversity when the 95% CIs did not overlap zero. This allowed us to compare gut microbiota diversity among the three plover species, while statistically controlling for differences in sex, age (for the combined dataset including both adults and juveniles), and year of sampling. To accommodate the non-independence of individuals belonging to the same/different families, Nest ID was incorporated as a random effect.

LMM for the combined dataset (juveniles and adults):

*Microbiota diversity* $\sim$ *Species + Sex + Age + Year + (1*|*Nest ID)*

LMM for the dataset split by age:

*Microbiota diversity* $\sim$ *Species + Sex + Year + (1*|*Nest ID)*

The significance of random effects was tested using the “ranova” function from the lmerTest package [72]. Marginal and conditional $R^2$ values were calculated using the MuMIn package [73]. Assumptions of normality and homogeneity of variance of residuals were examined through visual inspection of plots using the performance package [74] and were further assessed with Shapiro-Wilk tests. To meet these assumptions, Faith PD was square root transformed (except for the adults-only dataset), and the number of observed ASVs was log-transformed. Complete scripts and intermediate results can be found Appendix E.

#### Differential abundance analysis

A multivariate analysis by linear models as implemented in the MaAslin2 version 1.16.0 R package [75] was conducted to find associations between the variables of interest and microbial abundance of specific taxa. This analysis included: species, sex, age (for the combined dataset) and sampling year as fixed effects; and Nest ID as a random effect. As part of MaAslin2, the Holm-Bonferroni method [76] was employed to correct the *p*-values for multiple testing. A significance threshold of 0.05, a minimum relative abundance of 0.0001, and a minimum prevalence of 0.01 were set. Detailed scripts and models can be found in Appendix F.

## Results

Given the transient nature of gut microbiota during early development [4], we present the main results separately for adults and juveniles. Tables and figures of the combined dataset can be found in supplementary Appendix A. Of the 201 samples in our dataset, individuals with multiple sampling points (24 in total, reserved for future studies) were excluded. Additionally, individuals for whom molecular sex determination was unsuccessful were omitted, and only nests with multiple individuals were retained.Thus, our final dataset comprised 61 *A. pecuarius*, 56 *A. marginatus* and 19 *A. thoracicus* individuals (Table 1).

### Gut microbiota profile

**Fig. 2:**
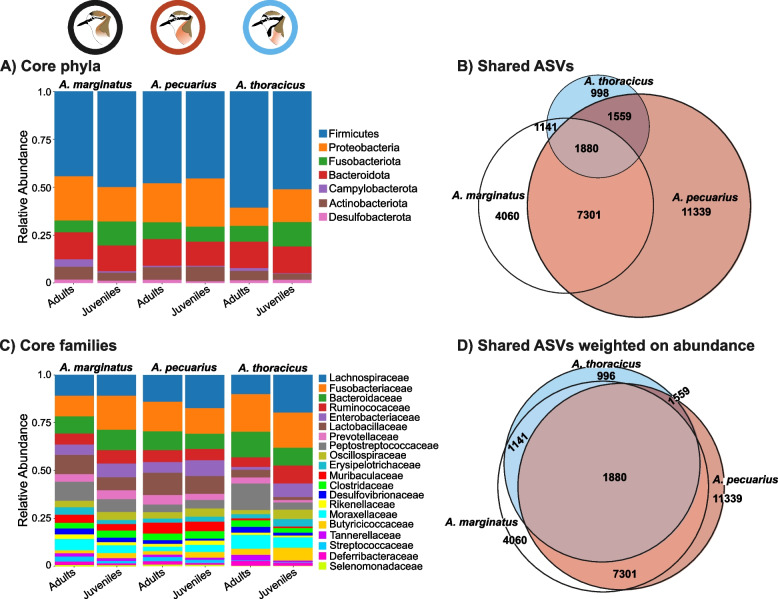
Relative abundances (as percentages) of of core gut microbiota decomposed by: **A** phyla and **C** families. Each species is represented by two age classes (adults and juveniles). Core taxa are defined as microbial taxa present in at least 95% of the samples. Venn diagrams representing **B** shared and unique ASVs among the three plover species, and **D** shared ASVs weighted by relative abundance

A total of 52 bacterial phyla were identified across the three species. Among these, the core gut microbiota consisted of eight phyla, the most abundant being *Firmicutes* (mean ± SD = 47.6 ± 18.6%), *Proteobacteria* (mean ± SD = 19.8 ± 16.34%), *Fusobacteria* (mean ± SD = 9.9 ± 12.05%), *Bacteroidota* (mean ± SD = 13.6 ± 9.6%), and *Actinobacteria* (mean ± SD = 5.8 ± 6.8%). In total, 596 bacterial families were identified, with 20 being classified as core taxa. The most prevalent among these families were *Lachnospiraceae* (mean ± SD = 14.31 ± 10.05%), *Fusobacteriaceae* (mean ± SD = 15.5 ± 15.44%), *Bacteroidaceae* (mean ± SD = 9.55 ± 9.16%), *Ruminococcaceae* (mean ± SD = 6.53 ± 6.09%) and *Enterobacteriaceae* (mean ± SD = 6.6 ± 9.65%) (Fig. 2A & C). A set of 1,880 ASVs were shared among all three species. Specifically, *A. marginatus* and *A. pecuarius* exhibited 7,301 shared ASVs, while 1,559 ASVs were common between *A. pecuarius* and *A. thoracicus* and 1,141 were shared between *A. marginatus* and *A. thoracicus* (Fig. 2B). Additionally, the shared ASVs were observed to be the most abundant, while those ASVs specific to individual species were among the least abundant (Fig. 2D).

### Gut microbiota composition (beta diversity)

**Fig. 3:**
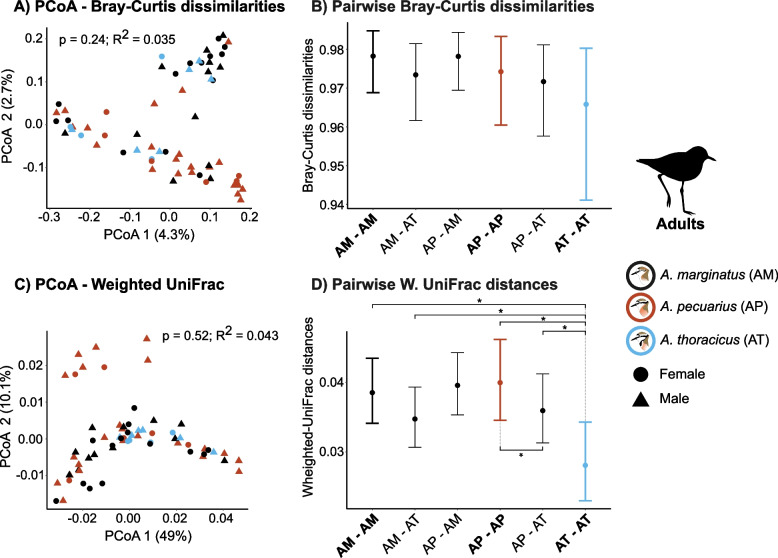
Composition differences (beta diversity) among adult individuals of the three plover species. The results of Principal Component Analyses (PCoA) are shown for **A** Bray-Curtis dissimilarities and **C** Weighted UniFrac distances. Results from PERMANOVAs including *p* and $R^2$ values are also given. **B** and **D** show the results of Bayesian pairwise models; asterisks indicate variables that are significantly associated with microbiota dissimilarity/distance (i.e. the 95% credible intervals do not overlap zero). AM-AM, AP-AP and AT-AT denote pairwise comparisons among pairs of individuals of the same species and are indicated in bold. AM-AT, AP-AM and AP-AT indicate pairwise comparisons among pairs of individuals of different species

**Fig. 4:**
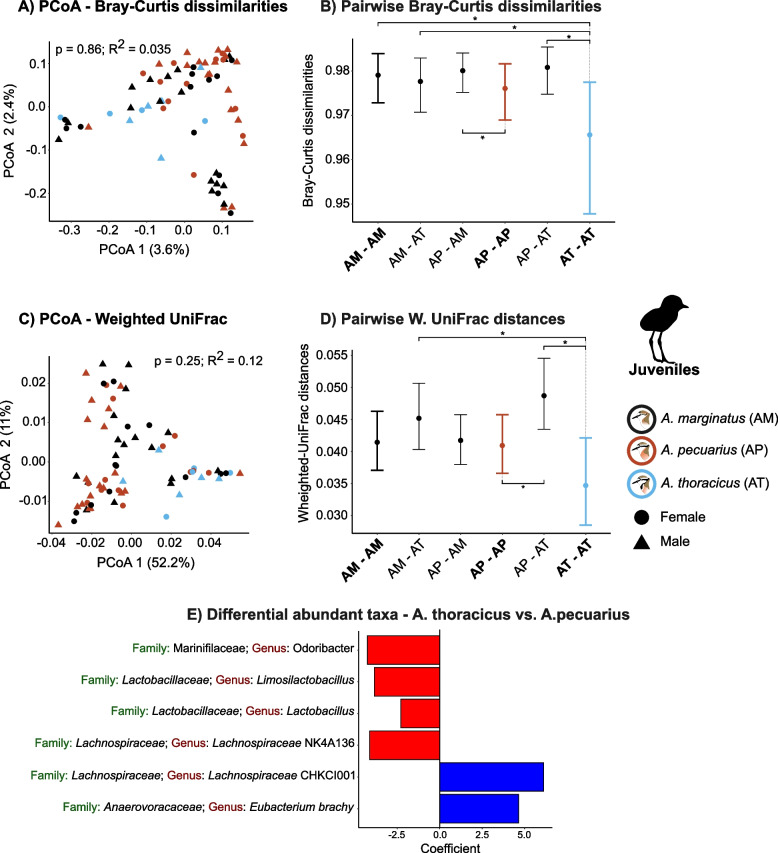
Composition differences (beta diversity) among adult individuals of the three plover species. The results of Principal Component Analyses (PCoA) are shown for **A** Bray-Curtis dissimilarities and **C** Weighted UniFrac distances. Results from PERMANOVAs including *p* and $R^2$ values are also given. **B** and **D** show the results of Bayesian pairwise models; asterisks indicate variables that are significantly associated with microbiota dissimilarity/distance (i.e. the 95% credible intervals do not overlap zero) AM-AM, AP-AP and AT-AT denote pairwise comparisons among pairs of individuals of the same species and are indicated in bold. AM-AT, AP-AM and AP-AT indicate pairwise comparisons among pairs of individuals of different species. **E** Bar plot showing differentially abundant microbial genera between *A. thoracicus* and *A. pecuarius* based on the output of MaAsLin2. Coefficients are presented for genera with corrected *p*-values < 0.05 and correspond to log2(fold change). Taxa that are less abundant in *A. thoracicus* compared to *A. pecuarius* are highlighted in red, while Taxa that are more abundant in *A. thoracicus* compared to *A. pecuarius* are highlighted in blue

Overall, the PERMANOVA analysis revealed no significant differences in gut microbiota composition among the three species based on both Bray-Curtis dissimilarities (BC) and Weighted UniFrac (WU) metrics (Figs. 3A & C; 4A & C; See Appendix A Fig. S2 A & C for the results for the adults and chicks combined). However, the permutation test for homogeneity of multivariate dispersion showed evidence for differences in group dispersion (Adults: $p_{\text {BC}} < 0.001$, $p_{\text {WU}} < 0.001$; Juveniles: $p_{\text {BC}} < 0.001$, $p_{\text {WU}} < 0.001$). These differences in dispersion were found when comparing *A. marginatus* with *A. thoracicus* (Adults: $p_{\text {BC}} < 0.001$, $p_{\text {WU}} < 0.003$; Juveniles: $p_{\text {BC}} < 0.001$ and *A.pecuarius* with *A.thoracicus* (Adults: $p_{\text {BC}} < 0.001$, $p_{\text {WU}} < 0.003$; Juveniles: $p_{\text {BC}} < 0.001$, $p_{\text {WU}} = 0.26$). Furthermore, our findings indicate no significant effects of sex, sampling year (Table 2), or age ($p_{\text {BC}} = 0.06$, $R^2_{\text {BC}} = 0.008$; $p_{\text {WU}} = 0.66$, $R^2_{\text {WU}} = 0.004$) on gut microbiota composition.

Similar findings were obtained from pairwise Bayesian regression models, revealing that most within-species pairwise dissimilarities were comparable to among-species differences (Figs. 3B & D; 4A & D; see Appendix A Fig. S2 B & D for the combined results for adults and chicks. A notable exception was observed in the endemic species, where for adults, WU distances within pairs of individuals of *A. thoracicus* were smaller than those between *A. thoracicus* and *A. pecuarius* ($\mu _{\text {WU}}$ = -0.25, CI [-0.39, -0.12]) and *A. thoracicus* and *A. marginatus* ($\mu _{\text {WU}}$ = -0.22, CI [-0.36, -0.08]; Fig. 3B & D). Furthermore, WU distances within adult individuals of *A. thoracicus* were notably lower compared to those within *A. pecuarius* ($\mu _{\text {WU}}$ = -0.37, CI [-0.58, -0.15] and *A. marginatus* ($\mu _{\text {WU}}$ = -0.33, CI [-0.54, -0.11]. Between-species pairwise differences were more pronounced in juveniles, where both BC dissimilarities and WU distances within pairs of *A. thoracicus* individuals were smaller than those between individuals of *A. thoracicus* and *A. pecuarius* ($\mu _{\text {BC}}$ = -0.61, CI [-0.88, -0.34]; $\mu _{\text {WU}}$ = -0.35, CI [-0.5, -0.21] and *A. marginatus* ($\mu _{\text {BC}}$ = -0.45, CI [-0.72, -0.18]; $\mu _{\text {WU}}$ = -0.28, CI [-0.42, -0.13]; Fig. 4B & D).

We also observed a contrasting pattern of WU distances in adults and juveniles when comparing distances within *A. pecuarius* with those between *A. pecuarius* and *A. thoracicus*. In adults, compositional distances within the same species were larger than those between different species, whereas in juveniles, the opposite pattern was observed, with within-species distances being smaller (Adults: $\mu _{\text {WU}}$ = 0.11, CI [0.01, 0.21]; Juveniles: $\mu _{\text {WU}}$ = -0.28, CI [-0.42, -0.13]; Figs. 3D & 4D).

Finally, we detected an influence of nest sharing on microbial community composition. Specifically, juvenile individuals from the same nest were compositionally similar to one another ($\mu _{\text {Same Nest}}$ = -0.36, CI [-0.96, -0.14]; Appendix A Fig. S3), while for adults, only microbial phylogenetic distances decreased between individuals of the same nest ($\mu _{\text {Same Nest}}$ = -0.16, CI [-0.32, -0.01]; Appendix A Fig. S3). No evidence was found for any effects of sex, year and age on gut microbial community composition (Appendix A Fig. S3 & S4). The Mantel test also revealed no significant correlation between the spatial distance among individuals and the composition of their gut microbiota communities (BC: r = 0.005, p = 0.7; WU: r = -0.03, p = 0.89; Appendix A Fig. S5).

### Differentially abundant taxa

Results from the combined dataset (adults and juveniles combined), revealed six differentially abundant microbial taxa between *A. thoracicus* and *A. pecuarius*, three between *A. thoracicus* and *A. marginatus* and two between *A. pecuarius* and *A. marginatus* (Appendix A Fig. S6). When focusing solely on juveniles, six differentially abundant bacterial genera were identified between *A. thoracicus* and *A. pecuarius*, ($p_{\text {adj}} < 0.05$; Fig. 4E). Among these, four genera exhibited significantly higher abundance in *A. pecuarius*: *Odoribacter*, *Limosilactobacillus*, *Lactobacillus*, and *Lachnospiraceae* NK4A136, while two genera exhibited significant higher abundance in *A. thoracicus*: *Lachnospiraceae* CHKCI001 and *Eubacterium brachy*. No differential abundant taxa were found in pairwise comparisons of adults of the three species or in association with any of the other studied variables.

**Table 1:** Summary of the number of analysed individuals per species

	N$^{\circ }$ individuals	Adults	Juveniles	Male (Adults)	Female (Adults)	Male (Juveniles)	Female (Juveniles)	2021	2022	Nests
**TOTAL**	136	65	71	41	24	41	30	95	41	55
***A. pecuarius***	61	29	32	23	6	19	13	49	12	27
***A. marginatus***	56	26	30	12	14	17	13	31	25	20
***A. thoracicus***	19	10	9	6	4	5	4	15	4	8

**Table 2:** The effect of species, sex, and sampling year on the gut microbial community composition of the three Madagascar plover species, as obtained from a PERMANOVA

		Bray-Curtis dissimilarities			Weighted UniFrac distances		
		R^2^	F	*p*-value	R^2^	F	*p*-value
**Adults**	**Species**	0.04	1.13	0.24	0.04	1.37	0.52
	**Sex**	0.02	1.00	0.26	0.01	0.61	0.82
	**Year**	0.02	1.05	0.92	0.01	0.43	0.72
**Juveniles**	**Species**	0.04	1.24	0.86	0.12	4.91	0.25
	**Sex**	0.01	0.96	0.81	0.03	2.14	0.24
	**Year**	0.02	1.11	0.51	0.01	1.04	0.22

**Fig. 5:**
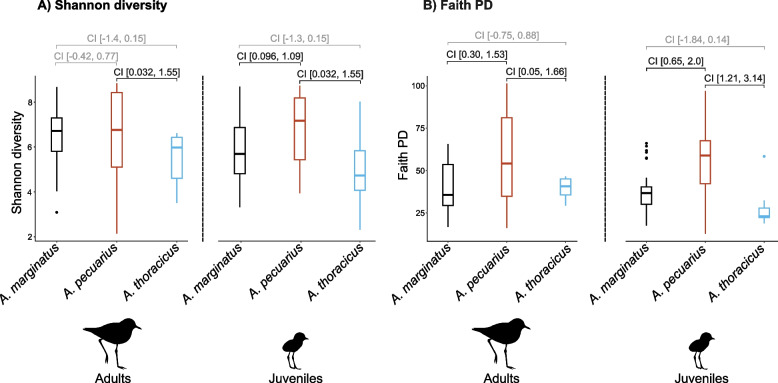
Microbiota diversity **A**) Shannon diversity index and **B**) Faith PD for adults and juveniles of the three plover species. The boxplots display the raw data, representing the interquartile range, with the horizontal line inside each box indicating the median and the vertical lines illustrating the spread and variability of the data. Upper and lower 95% confidence intervals are indicated for each species comparison, with intervals not crossing zero (i.e. significant differences) depicted in black

### Gut microbiota diversity

The sole factor explaining differences in gut bacterial diversity was species identity. Analysis of the combined dataset including both adults and juveniles revealed that *A. pecuarius* exhibited higher gut microbiota diversity, followed by *A. marginatus*, with the endemic species *A. thoracicus* showing the lowest microbiota diversity (Appendix A Fig. S2 E & F). These differences appear to be more pronounced in juveniles. Our findings indicate significant variability in Shannon’s diversity and Faith’s PD between *A. pecuarius* and *A. marginatus* and between *A. thoracicus* and *A. pecuarius* (Fig. 5). However, no diversity differences were found between *A. thoracicus* and *A. marginatus* (Fig. 5). Among adults, differences in Shannon’s diversity were observed only between *A.pecuarius* and *A. thoracicus*. The results with Faith’s PD mirrored those observed in juvenile individuals (Fig. 5). Analysis of the significance of the random effects revealed no evidence for variation driven by the grouping factor Nest ID (Adults: $p_{\text {Shannon}} = 0.17$; $p_{\text {Faith PD}} = 0.08$; Juveniles: $p_{\text {Shannon}} = 0.98$; $p_{\text {Faith PD}} = 1$). As the results for the number of observed ASVs were similar to those for Faith’s PD, they are presented in the supplementary materials (Appendix A Fig. S7).

## Discussion

Genetic factors, including host species and individual genetic variation, have been associated with differences in microbial community structure and diversity [77, 78]. Leveraging the unique ecological context provided by Madagascar, we investigated the impact of host species on gut microbiota composition across three plover species breeding in sympatry. We found no evidence for compositional differences in the gut microbiota among the three plover species, suggesting that shared habitat and diet may exert a stronger influence on the gut microbiota than host species. Furthermore, our findings suggest that differences in microbial diversity among the three plover species appear to reflect broader ecological characteristics such as population size, dispersal ability and genetic diversity.

### No between-species differences in microbiota composition

We observed no discernible differences in gut microbiota composition among the three sympatric plover species. Despite the presence of unique ASVs within each species, these were generally rare or of low abundance and did not contribute significantly to interspecies differences. Our results suggest that shared habitat and diet may facilitate the interspecific transmission of gut bacteria, overshadowing the influence of species evolutionary differences. This may be amplified by the precocial and ground-nesting nature of plovers, as they are in direct contact with their environment from an early age [79]. Interestingly, our results revealed that specific taxa were differentially abundant among juveniles of the closely related species *A. thoracicus* and *A. pecuarius*, but not among adults. This finding suggests that, while species-specific differences might emerge early in life, the subsequent acquisition of environmental microbes likely homogenises the gut microbiota across the three plover species as they mature.

Comparative studies of other shorebird species and avian taxa corroborate our findings by showing that, more generally, habitat and dietary specialisation strongly shape gut microbiota composition [5, 19, 80]. For example, a study of nine Darwin’s finch species showed that habitat, rather than species identity, was the primary determinant of microbial differences among individuals [19]. However, an exception was observed in the vampire finch (*Geospiza septentrionalis*), a dietary specialist that occasionally feeds on the blood of other birds, which exhibits a distinct gut microbiota composition [81]. Similar observations were made in two closely related species of common nightingale (*Luscinia megarhynchos* and *L. luscinia*), where gut microbiota composition did not differ significantly between sympatric or allopatric populations [21]. A prevailing hypothesis to explain the limited phylogenetic signal in avian gut microbiota compared to non-flying mammals is related to the evolutionary adaptation of birds to flight. This adaptation results in shorter gut retention times (from ingestion to defecation) and simplified gut environments [17]. As a consequence, birds may experience a higher turnover in their gut microbiota, leading to diet and the environment playing a more dominant role in determining microbial community structure than host phylogeny [18].

As previously mentioned, the influence of evolutionary divergence on microbiota composition appears to be more pronounced in mammals [17]. For example, a study of six sympatric Malagasy mammals revealed distinct microbial compositions unique to each species [82]. However, species sharing terrestrial habitats (as is the case of the three plover species in our study), exhibited similar microbiota compositions. This may hint at the potential role of ground-dwelling behaviour in facilitating the indirect horizontal transmission of commensal gut bacteria among sympatric wild animals [82].

### Higher compositional similarity and lower diversity in the endemic *A. thoracicus*

The relationship between the gut microbiota and host fitness is increasingly being recognized [83, 84], emphasising the need to consider host-microbe interactions in the context of environmental adaptation. Our study uncovered intriguing patterns in the gut microbiota of the vulnerable [40] endemic *A. thoracicus*. Within-species comparisons showed higher microbial compositional similarity and lower microbiota diversity in *A. thoracicus*, especially among juveniles. Diversity differences were also evident between *A. thoracicus* and *A. pecuarius*, and between *A. pecuarius* and *A. marginatus*. Although similar patterns were observed in both juveniles and adults, microbiota diversity differences appeared to be more noticeable among juvenile individuals.

Interestingly, the observed compositional similarities within the endemic species and the patterns of microbial diversity observed (*A. pecuarius* > *A. marginatus* > *A. thoracicus*) appear to mirror broader ecological trends, including population size, island distribution/dispersion, and genetic diversity (Fig. 1). For instance, *A. thoracicus*, which has the smallest population size, high site specialization, low dispersal capacity, and reduced genetic diversity [33, 41], exhibited higher intraspecific microbial composition similarity and lower gut microbiota diversity. Conversely, *A. pecuarius*, with its wide geographical distribution, greater dispersal ability, and high genetic diversity, exhibited greater microbial diversity [33, 41]. While our empirical data are not suggestive of any significant dietary differences among the three species, it is possible that species with greater dispersal ability, like *A. pecuarius*, may have access to more varied diets, which could contribute to their more diverse gut microbiota. To fully explore this hypothesis, a study investigating the dietary preferences of these species across different regions of the island (not just within our study site) would be required.

Species with small population sizes, habitat specialisation and poor dispersal ability, such as the endemic *A. thoracicus* [39], often confront challenges like inbreeding depression and reduced genetic diversity, which can decrease adaptive potential and increase extinction risk [25, 27, 85]. While this has mainly been studied from the perspective of the host genome, there is growing recognition of the need to consider the hologenome, i.e. the collective genomic content of the host-microbiome ecosystem [2]. This is because a diverse microbiota may provide a wider array of functions performed by different microbial taxa, potentially offering numerous benefits to the host [86]. Accordingly, demographic reductions and the loss of genetic diversity can disrupt the composition of the microbiota, potentially impacting host fitness and accelerating population declines [28, 31, 87]. For example, a recent experimental study by Ørsted et al. [29] showed that population bottlenecks in *Drosophila melanogaster* constrain microbiota richness and diversity, with both microbiota and host genetic diversity influencing population fitness. Similarly, a study by Greenspan et al. [30] on 43 threatened and 90 non-threatened amphibian species revealed that threatened species with narrow environmental tolerances or habitat specialization tended to have lower skin microbiota diversity.

Our findings might be relevant for the conservation of the endemic Madagascar plover. The observed similarities in microbial composition and the reduced microbial diversity of this species may hinder its ability to adapt to a changing environment [28, 88]. Furthermore, a high degree of specialisation may limit the capacity of this species to cope with dietary shifts and environmental stressors [31], particularly in a region of Madagascar that is increasingly subjected to anthropogenic change [33]. Research on other species suggests that changes in the gut microbiota can enable trophic niche expansion. For example, in piscivorous bats, incorporating fish into their diet led to the acquisition of beneficial bacteria not typically available in terrestrial environments. These microbes became established across populations, allowing the bats to successfully exploit a new ecological niche [89]. This capacity for microbiota-mediated adaptability could potentially be important for the long-term survival of *A. thoracicus* as it could offer a mechanism to adjust to shifts in available resources or environmental conditions [32, 88, 90].

### Gut microbiota composition affected by relatedness but not spatial distance

The three plover species in our study bred and were captured in a uniform habitat dominated by salt marsh habitats [33]. To further substantiate our claims of a shared, uniform habitat among the three plover species, we investigated whether the spatial distance between individuals influenced gut microbiota composition. Our results indicated that geographical distance does not exert a strong influence on gut microbiota composition. Previous research across a range of vertebrate species suggests that gut microbiota similarity declines with increasing geographic distance, implying that physical distance can be a barrier to microbial dispersal [91–93]. Conversely, studies on avian species have found either no or weak associations between gut microbiota composition and geographic distance [21, 93, 94]. This difference may be attributed to the inherent mobility of many bird species, which allows them to traverse large distances [21, 93, 94]. However, due to the relatively small size of our study area (approximately 8 km$^2$, with the largest distance between individuals being around 20 km), we cannot rule out the possibility of differences in gut microbiota composition among the three plover species over larger distances.

We also found a significant influence of genetic relatedness on microbiota composition, with individuals from the same family exhibiting greater similarity compared to those from different families. This aligns with existing knowledge about the roles of parental care, the nest environment and interactions among nestlings in shaping the early microbiota of altricial species [6]. While numerous studies have found strong correlations between the nesting environment and gut microbiota in altricial species [95–98] these interactions appear to be less pronounced in precocial species [99]. For example, research on Arctic shorebirds found that chicks primarily acquire gut microbiota after hatching, with a stable community established within three days, mainly sourced from the environment [100]. Although the three Madagascar plover species studied here commence feeding independently upon hatching, they still receive parental brooding and protection, with the parents guiding the chicks to foraging areas [33]. Thus, while the gut microbiota of plovers may be primarily influenced by horizontal acquisition from the environment, vertical transmission between parents and chicks might also still occur.

## Conclusion

We investigated the influence of host species on gut microbiota composition across three sympatric plover species. Our findings revealed no discernible compositional differences among the species, although notable differences in microbial diversity were observed. The endemic *A. thoracicus* exhibited higher intraspecific compositional similarity and lower gut microbiota diversity. These differences appear to be primarily driven by variation in early developmental stages, suggesting that species-specific differences in the gut microbiota may emerge early in life but are subsequently homogenised by long-term exposure to a shared habitat and diet. Additionally, the precocial and ground-dwelling nature of plovers likely facilitates the horizontal transmission of commensal gut bacteria. Patterns of gut microbiota diversity may reflect broader ecological trends including population size, island dispersal and genetic diversity. The observed similarities in composition, coupled with reduced microbial and genetic diversity in *A. thoracicus*, may impede this species’ ability to adapt to environmental changes, further emphasising the vulnerability of this island endemic.

## Supplementary information

Additional file 1. Appendix A. Supplementary tables and figures.

Additional file 2. Appendix B. Quality control workflow and results.

Additional file 3. Appendix C. Sequence data processing.

Additional file 4. Appendix D. Beta diversity statistical analysis.

Additional file 5. Appendix E. Alpha diversity statistical analysis.

Additional file 6. Appendix F. Differential abundance analysis.

## Data Availability

All 16S rRNA raw reads have been submitted to the European Nucleotide Archive repository, Project ID: PRJEB76685.The scripts and metadata to reproduce all analyses can be accessed via the GitHub repository: https://github.com/hugoeira/Gut-microbiota-Madagascar-plovers.

## References

[1] Rosenberg E. Microbiomes: Current Knowledge and Unanswered Questions. Cham: Springer Nature Switzerland AG; 2021.

[2] Rosenberg E, Zilber-Rosenberg I. The hologenome concept of evolution after 10 years. Microbiome. 2018;6:1–14. 29695294 10.1186/s40168-018-0457-9 PMC5922317

[3] Těšickỳ M, Schmiedová L, Krajzingrová T, Samblas MG, Bauerová P, Kreisinger J, et al. Nearly (?) sterile avian egg in a passerine bird. FEMS Microbiol Ecol. 2024;100(1):fiad164. 10.1093/femsec/fiad164 PMC10791042 38115624

[4] van Dongen WF, White J, Brandl HB, Moodley Y, Merkling T, Leclaire S, et al. Age-related differences in the cloacal microbiota of a wild bird species. BMC Ecol. 2013;13:1–12. 23531085 10.1186/1472-6785-13-11 PMC3668179

[5] Grond K, Sandercock BK, Jumpponen A, Zeglin LH. The avian gut microbiota: community, physiology and function in wild birds. J Avian Biol. 2018;49(11): e01788.

[6] Campos-Cerda F, Bohannan BJ. The nidobiome: a framework for understanding microbiome assembly in neonates. Trends Ecol Evol. 2020;35(7):573–82. 32360079 10.1016/j.tree.2020.03.007

[7] Sun F, Chen J, Liu K, Tang M, Yang Y. The avian gut microbiota: diversity, influencing factors, and future directions. Front Microbiol. 2022;13. 10.3389/fmicb.2022.934272 PMC9389168 35992664

[8] Waite DW, Taylor MW. Characterizing the avian gut microbiota: membership, driving influences, and potential function. Front Microbiol. 2014;5:91622. 10.3389/fmicb.2014.00223 PMC4032936 24904538

[9] Rothschild D, Weissbrod O, Barkan E, Kurilshikov A, Korem T, Zeevi D, et al. Environment dominates over host genetics in shaping human gut microbiota. Nature. 2018;555(7695):210–5. 29489753 10.1038/nature25973

[10] Youngblut ND, Reischer GH, Walters W, Schuster N, Walzer C, Stalder G, et al. Host diet and evolutionary history explain different aspects of gut microbiome diversity among vertebrate clades. Nat Commun. 2019;10(1):1–15. 31097702 10.1038/s41467-019-10191-3 PMC6522487

[11] Grond K, Louyakis AS, Hird SM. Functional and compositional changes in the fecal microbiome of a shorebird during migratory stopover. MSystems. 2023;8(2):e01128-22. 36786579 10.1128/msystems.01128-22 PMC10134852

[12] Ley RE, Hamady M, Lozupone C, Turnbaugh PJ, Ramey RR, Bircher JS, et al. Evolution of mammals and their gut microbes. Science. 2008;320(5883):1647–51. 18497261 10.1126/science.1155725 PMC2649005

[13] Goodrich JK, Waters JL, Poole AC, Sutter JL, Koren O, Blekhman R, et al. Human genetics shape the gut microbiome. Cell. 2014;159(4):789–99. 25417156 10.1016/j.cell.2014.09.053 PMC4255478

[14] Muegge BD, Kuczynski J, Knights D, Clemente JC, González A, Fontana L, et al. Diet drives convergence in gut microbiome functions across mammalian phylogeny and within humans. Science. 2011;332(6032):970–4. 21596990 10.1126/science.1198719 PMC3303602

[15] Spor A, Koren O, Ley R. Unravelling the effects of the environment and host genotype on the gut microbiome. Nat Rev Microbiol. 2011;9(4):279–90. 21407244 10.1038/nrmicro2540

[16] Brooks AW, Kohl KD, Brucker RM, van Opstal EJ, Bordenstein SR. Phylosymbiosis: relationships and functional effects of microbial communities across host evolutionary history. PLoS Biol. 2016;14(11): e2000225. 27861590 10.1371/journal.pbio.2000225 PMC5115861

[17] Song SJ, Sanders JG, Delsuc F, Metcalf J, Amato K, Taylor MW, et al. Comparative analyses of vertebrate gut microbiomes reveal convergence between birds and bats. MBio. 2020;11(1):10–1128. 10.1128/mBio.02901-19 PMC6946802 31911491

[18] Bodawatta KH, Koane B, Maiah G, Sam K, Poulsen M, Jønsson KA. Species-specific but not phylosymbiotic gut microbiomes of New Guinean passerine birds are shaped by diet and flight-associated gut modifications. Proc R Soc B. 1949;2021(288):20210446. 10.1098/rspb.2021.0446 PMC8059580 33878920

[19] Loo WT, García-Loor J, Dudaniec RY, Kleindorfer S, Cavanaugh CM. Host phylogeny, diet, and habitat differentiate the gut microbiomes of Darwin’s finches on Santa Cruz Island. Sci Rep. 2019;9(1):18781. 31827126 10.1038/s41598-019-54869-6 PMC6906294

[20] San Juan PA, Castro I, Dhami MK. Captivity reduces diversity and shifts composition of the Brown Kiwi microbiome. Anim Microbiome. 2021;3:1–8. 34238378 10.1186/s42523-021-00109-0 PMC8268595

[21] Sottas C, Schmiedová L, Kreisinger J, Albrecht T, Reif J, Osiejuk TS, et al. Gut microbiota in two recently diverged passerine species: evaluating the effects of species identity, habitat use and geographic distance. BMC Ecol Evol. 2021;21:1–14. 33691625 10.1186/s12862-021-01773-1 PMC7948333

[22] Bray JR, Curtis JT. An ordination of the upland forest communities of southern Wisconsin. Ecol Monogr. 1957;27(4):326–49.

[23] Kristensen TN, Schönherz AA, Rohde PD, Sørensen JG, Loeschcke V. Strong experimental support for the hologenome hypothesis revealed from Drosophila melanogaster selection lines. bioRxiv. 2021:2021–09. 10.1101/2021.09.09.459587.

[24] Spielman D, Brook BW, Frankham R. Most species are not driven to extinction before genetic factors impact them. Proc Natl Acad Sci. 2004;101(42):15261–4. 15477597 10.1073/pnas.0403809101 PMC524053

[25] Markert JA, Champlin DM, Gutjahr-Gobell R, Grear JS, Kuhn A, McGreevy TJ, et al. Population genetic diversity and fitness in multiple environments. BMC Evol Biol. 2010;10:1–13. 20609254 10.1186/1471-2148-10-205 PMC2927917

[26] Hoffmann AA, Sgrò CM, Kristensen TN. Revisiting adaptive potential, population size, and conservation. Trends Ecol Evol. 2017;32(7):506–17. 28476215 10.1016/j.tree.2017.03.012

[27] Ørsted M, Hoffmann AA, Sverrisdóttir E, Nielsen KL, Kristensen TN. Genomic variation predicts adaptive evolutionary responses better than population bottleneck history. PLoS Genet. 2019;15(6): e1008205. 31188830 10.1371/journal.pgen.1008205 PMC6590832

[28] Trevelline BK, Fontaine SS, Hartup BK, Kohl KD. Conservation biology needs a microbial renaissance: a call for the consideration of host-associated microbiota in wildlife management practices. Proc R Soc B. 1895;2019(286):20182448. 10.1098/rspb.2018.2448 PMC6364583 30963956

[29] Ørsted M, Yashiro E, Hoffmann AA, Kristensen TN. Population bottlenecks constrain host microbiome diversity and genetic variation impeding fitness. PLoS Genet. 2022;18(5): e1010206. 35604942 10.1371/journal.pgen.1010206 PMC9166449

[30] Greenspan SE, Peloso P, Fuentes-González JA, Bletz M, Lyra ML, Machado IF, et al. Low microbiome diversity in threatened amphibians from two biodiversity hotspots. Anim Microbiome. 2022;4(1):69. 36582011 10.1186/s42523-022-00220-w PMC9801548

[31] West AG, Waite DW, Deines P, Bourne DG, Digby A, McKenzie VJ, et al. The microbiome in threatened species conservation. Biol Conserv. 2019;229:85–98.

[32] Wasimuddin, Malik H, Ratovonamana YR, Rakotondranary SJ, Ganzhorn JU, Sommer S. Anthropogenic disturbance impacts gut microbiome homeostasis in a Malagasy primate. Front Microbiol. 2022;13:911275. 10.3389/fmicb.2022.911275 PMC9253676 35801106

[33] Zefania S, Székely T. Charadrius spp. In: Safford R, Hawkins F, editors. The Birds of Africa: Volume VIII: The Malagasy Region: Madagascar, Seychelles, Comoros, Mascarenes. Bloomsbury; 2013.

[34] Székely T, Kosztolányi A, Küpper C. Practical guide for investigating breeding ecology of Kentish plover Charadrius alexandrinus. Version 3., Unpublished Report, University of Bath. 2008. https://www.pennuti.net/wp-content/uploads/2010/08/KP_Field_Guide_v3.pdf.

[35] Dos Remedios N, Küpper C, Székely T, Zefania S, Burns F, Bolton M, et al. Genetic structure among Charadrius plovers on the African mainland and islands of Madagascar and St Helena. Ibis. 2020;162(1):104–18.

[36] Černỳ D, Natale R. Comprehensive taxon sampling and vetted fossils help clarify the time tree of shorebirds (Aves, Charadriiformes). Mol Phylogenet Evol. 2022;177: 107620. 36038056 10.1016/j.ympev.2022.107620

[37] Delany S, Scott D, Helmink A, Dodman T, Flink S, Stroud D, et al. An atlas of wader populations in Africa and Western Eurasia. Wetlands International; 2009.

[38] Long P, Zefania S, Ffrench-Constant R, Székely T. Estimating the population size of an endangered shorebird, the Madagascar plover, using a habitat suitability model. Anim Conserv. 2008;11(2):118–27.

[39] Zefania S, Ffrench-Constant R, Long PR, Székely T. Breeding distribution and ecology of the threatened Madagascar plover Charadrius thoracicus. Ostrich-J Afr Ornithol. 2008;79(1):43–51.

[40] BirdLife International. Charadrius thoracicus. The IUCN Red List of Threatened Species 2023. 10.2305/IUCN.UK.2023-1.RLTS.T22693780A231072418.en. Accessed 7 May 2024

[41] Eberhart-Phillips LJ, Hoffman JI, Brede EG, Zefania S, Kamrad MJ, Székely T, et al. Contrasting genetic diversity and population structure among three sympatric Madagascan shorebirds: parallels with rarity, endemism, and dispersal. Ecol Evol. 2015;5(5):997–1010. 25798218 10.1002/ece3.1393 PMC4364815

[42] Parra JE, Beltrán M, Zefania S, Dos Remedios N, Székely T. Experimental assessment of mating opportunities in three shorebird species. Anim Behav. 2014;90:83–90.

[43] Hall LK, Cavitt JF. Comparative study of trapping methods for ground-nesting shorebirds. Waterbirds. 2012;35(2):342–6.

[44] Zefania S, Emilienne R, Faria PJ, Bruford MW, Long PR, Székely T. Cryptic sexual size dimorphism in Malagasy plovers Charadrius spp. Ostrich. 2010;81(3):173–8.

[45] Fridolfsson AK, Ellegren H. A simple and universal method for molecular sexing of non-ratite birds. J Avian Biol. 1999;30:116–21.

[46] Knutie SA, Gotanda KM. A non-invasive method to collect fecal samples from wild birds for microbiome studies. Microb Ecol. 2018;76:851–5. 29623358 10.1007/s00248-018-1182-4

[47] Klindworth A, Pruesse E, Schweer T, Peplies J, Quast C, Horn M, et al. Evaluation of general 16S ribosomal RNA gene PCR primers for classical and next-generation sequencing-based diversity studies. Nucleic Acids Res. 2013;41(1):e1–e1. 22933715 10.1093/nar/gks808 PMC3592464

[48] Bolyen E, Rideout JR, Dillon MR, Bokulich NA, Abnet CC, Al-Ghalith GA, et al. Reproducible, interactive, scalable and extensible microbiome data science using QIIME 2. Nat Biotechnol. 2019;37(8):852–7. 31341288 10.1038/s41587-019-0209-9 PMC7015180

[49] Callahan BJ, McMurdie PJ, Rosen MJ, Han AW, Johnson AJA, Holmes SP. DADA2: High-resolution sample inference from Illumina amplicon data. Nat Methods. 2016;13(7):581–3. 27214047 10.1038/nmeth.3869 PMC4927377

[50] Quast C, Pruesse E, Yilmaz P, Gerken J, Schweer T, Yarza P, et al. The SILVA ribosomal RNA gene database project: improved data processing and web-based tools. Nucleic Acids Res. 2012;41(D1):D590-6. 23193283 10.1093/nar/gks1219 PMC3531112

[51] Robeson MS, O’Rourke DR, Kaehler BD, Ziemski M, Dillon MR, Foster JT, et al. RESCRIPt: Reproducible sequence taxonomy reference database management. PLoS Comput Biol. 2021;17(11): e1009581. 34748542 10.1371/journal.pcbi.1009581 PMC8601625

[52] R Core Team. R: A Language and Environment for Statistical Computing. Vienna Austria; 2013. https://www.R-project.org/. Accessed May 2024.

[53] Bisanz JE. qiime2R: Importing QIIME2 artifacts and associated data into R sessions. 2018. V0.99. https://github.com/jbisanz/qiime2R. Accessed May 2024.

[54] Davis NM, Proctor DM, Holmes SP, Relman DA, Callahan BJ. Simple statistical identification and removal of contaminant sequences in marker-gene and metagenomics data. Microbiome. 2018;6:1–14. 30558668 10.1186/s40168-018-0605-2 PMC6298009

[55] McMurdie PJ, Holmes S. phyloseq: An R package for reproducible interactive analysis and graphics of microbiome census data. PLoS ONE. 2013;8(4):e61217. http://dx.plos.org/10.1371/journal.pone.0061217. Accessed May 2024. 10.1371/journal.pone.0061217 PMC3632530 23630581

[56] Katoh K, Misawa K, Kuma Ki, Miyata T. MAFFT: a novel method for rapid multiple sequence alignment based on fast Fourier transform. Nucleic Acids Res. 2002;30(14):3059–66. 10.1093/nar/gkf436 PMC135756 12136088

[57] Price MN, Dehal PS, Arkin AP. FastTree 2-approximately maximum-likelihood trees for large alignments. PLoS ONE. 2010;5(3): e9490. 20224823 10.1371/journal.pone.0009490 PMC2835736

[58] Wickham H. ggplot2: Elegant Graphics for Data Analysis. Springer-Verlag New York; 2016. https://ggplot2.tidyverse.org. Accessed May 2024.

[59] Russel J. MicEco: Various functions for microbial community data. 2024. R package version 0.9.19. https://github.com/Russel88/MicEco.

[60] Paulson JN, Stine OC, Bravo HC, Pop M. Differential abundance analysis for microbial marker-gene surveys. Nat Methods. 2013;10(12):1200–2. 24076764 10.1038/nmeth.2658 PMC4010126

[61] Paulson JN, Pop M, Bravo HC. metagenomeSeq: Statistical analysis for sparse high-throughput sequencing. Bioconductor Packag. 2013;1:191.

[62] Lozupone CA, Hamady M, Kelley ST, Knight R. Quantitative and qualitative *β *diversity measures lead to different insights into factors that structure microbial communities. Appl Environ Microbiol. 2007;73(5):1576–85. 10.1128/AEM.01996-06 PMC1828774 17220268

[63] Oksanen J, Simpson GL, Blanchet FG, Kindt R, Legendre P, Minchin PR, et al. vegan: Community Ecology Package. 2022. https://CRAN.R-project.org/package=vegan. R package version 2.6-4. Accessed May 2024.

[64] Raulo A, Allen BE, Troitsky T, Husby A, Firth JA, Coulson T, et al. Social networks strongly predict the gut microbiota of wild mice. ISME J. 2021;15(9):2601–13. 33731838 10.1038/s41396-021-00949-3 PMC8397773

[65] Bürkner PC. brms: An R package for Bayesian multilevel models using Stan. J Stat Softw. 2017;80:1–28.

[66] Bürkner PC. Advanced Bayesian multilevel modeling with the R package brms. 2017. arXiv preprint arXiv:1705.11123.

[67] Rohlf FJ, Sokal RR. Biometry: the principles and practice of statistics in biological research. Systematic Zoology. 1970;19(4):391–3.

[68] Shannon CE. A mathematical theory of communication. Bell Syst Tech J. 1948;27(3):379–423.

[69] Faith DP, Baker AM. Phylogenetic diversity (PD) and biodiversity conservation: some bioinformatics challenges. Evol Bioinforma. 2006;2:117693430600200000. PMC2674678 19455206

[70] Bates D, Mächler M, Bolker B, Walker S. Fitting Linear Mixed-Effects Models Using lme4. J Stat Softw. 2015;67(1):1–48. 10.18637/jss.v067.i01.

[71] Loy A, Steele S, Korobova J. lmeresampler: Bootstrap Methods for Nested Linear Mixed-Effects Models. 2023. https://CRAN.R-project.org/package=lmeresampler. R package version 0.2.4. Accessed May 2024.

[72] Kuznetsova A, Brockhoff PB, Christensen RHB, et al. lmerTest package: tests in linear mixed effects models. J Stat Softw. 2017;82(13):1–26.

[73] Bartoń K. MuMIn: Multi-Model Inference. 2023. https://CRAN.R-project.org/package=MuMIn. R package version 1.47.5. Accessed May 2024.

[74] Lüdecke D, Ben-Shachar MS, Patil I, Waggoner P, Makowski D. performance: An R Package for Assessment, Comparison and Testing of Statistical Models. J Open Source Softw. 2021;6(60):3139. 10.21105/joss.03139.

[75] Mallick H, Rahnavard A, McIver L. MaAsLin 2: multivariable association in population-scale meta-omics studies. R/Bioconductor Package. 2020. 10.1371/journal.pcbi.1009442 PMC8714082 34784344

[76] Holm S. A simple sequentially rejective multiple test procedure. Scand J Stat. 1979;6(2):65–70.

[77] Groussin M, Mazel F, Sanders JG, Smillie CS, Lavergne S, Thuiller W, et al. Unraveling the processes shaping mammalian gut microbiomes over evolutionary time. Nat Commun. 2017;8(1):14319. 28230052 10.1038/ncomms14319 PMC5331214

[78] Grosser S, Sauer J, Paijmans AJ, Caspers BA, Forcada J, Wolf JB, et al. Fur seal microbiota are shaped by the social and physical environment, show mother-offspring similarities and are associated with host genetic quality. Mol Ecol. 2019;28(9):2406–22. 30849214 10.1111/mec.15070

[79] Székely T. Why study plovers? The significance of non-model organisms in avian ecology, behaviour and evolution. J Ornithol. 2019;160(3):923–33.

[80] Lu Z, Li S, Wang M, Wang C, Meng D, Liu J. Comparative analysis of the gut microbiota of three sympatric terrestrial wild bird species overwintering in farmland habitats. Front Microbiol. 2022;13: 905668. 35928156 10.3389/fmicb.2022.905668 PMC9343720

[81] Michel AJ, Ward LM, Goffredi SK, Dawson KS, Baldassarre DT, Brenner A, et al. The gut of the finch: uniqueness of the gut microbiome of the Galápagos vampire finch. Microbiome. 2018;6:1–14. 30231937 10.1186/s40168-018-0555-8 PMC6146768

[82] Perofsky AC, Lewis RJ, Meyers LA. Terrestriality and bacterial transfer: a comparative study of gut microbiomes in sympatric Malagasy mammals. ISME J. 2019;13(1):50–63. 30108305 10.1038/s41396-018-0251-5 PMC6299109

[83] Suzuki TA. Links between natural variation in the microbiome and host fitness in wild mammals. Integr Comp Biol. 2017;57(4):756–69. 28992216 10.1093/icb/icx104

[84] Gould AL, Zhang V, Lamberti L, Jones EW, Obadia B, Korasidis N, et al. Microbiome interactions shape host fitness. Proc Natl Acad Sci. 2018;115(51):E11951-60. 30510004 10.1073/pnas.1809349115 PMC6304949

[85] Willi Y, Kristensen TN, Sgrò CM, Weeks AR, Ørsted M, Hoffmann AA. Conservation genetics as a management tool: The five best-supported paradigms to assist the management of threatened species. Proc Natl Acad Sci. 2022;119(1). 10.1073/pnas.2105076119 PMC8740573 34930821

[86] Heiman ML, Greenway FL. A healthy gastrointestinal microbiome is dependent on dietary diversity. Mol Metab. 2016;5(5):317–20. 10.1016/j.molmet.2016.02.005 PMC4837298 27110483

[87] Bahrndorff S, Alemu T, Alemneh T, Lund Nielsen J. The microbiome of animals: implications for conservation biology. Int J Genomics. 2016;1:5304028. 10.1155/2016/5304028 PMC4852354 27195280

[88] Peixoto RS, Voolstra CR, Sweet M, Duarte CM, Carvalho S, Villela H, et al. Harnessing the microbiome to prevent global biodiversity loss. Nat Microbiol. 2022;7(11):1726–35. 35864220 10.1038/s41564-022-01173-1

[89] Aizpurua O, Nyholm L, Morris E, Chaverri G, Herrera Montalvo LG, Flores-Martinez JJ, et al. The role of the gut microbiota in the dietary niche expansion of fishing bats. Anim Microbiome. 2021;3:1–14. 34711286 10.1186/s42523-021-00137-w PMC8555116

[90] Jones W, Eberhart-Hertel LJ, Freckleton RP, Hoffman JI, Krüger O, Sandercock BK, et al. Exceptionally high apparent adult survival in three tropical species of plovers in Madagascar. J Avian Biol. 2022;2022(1).

[91] Linnenbrink M, Wang J, Hardouin EA, Künzel S, Metzler D, Baines JF. The role of biogeography in shaping diversity of the intestinal microbiota in house mice. Mol Ecol. 2013;22(7):1904–16. 23398547 10.1111/mec.12206

[92] Suzuki TA, Worobey M. Geographical variation of human gut microbial composition. Biol Lett. 2014;10(2):20131037. 24522631 10.1098/rsbl.2013.1037 PMC3949373

[93] Hird SM, Carstens BC, Cardiff SW, Dittmann DL, Brumfield RT. Sampling locality is more detectable than taxonomy or ecology in the gut microbiota of the brood-parasitic Brown-headed Cowbird (Molothrus ater). PeerJ. 2014;2: e321. 24711971 10.7717/peerj.321 PMC3970801

[94] Kropáčková L, Těšickỳ M, Albrecht T, Kubovčiak J, Čížková D, Tomášek O, et al. Codiversification of gastrointestinal microbiota and phylogeny in passerines is not explained by ecological divergence. Mol Ecol. 2017;26(19):5292–304. 28401612 10.1111/mec.14144

[95] Chen CY, Chen CK, Chen YY, Fang A, Shaw GTW, Hung CM, et al. Maternal gut microbes shape the early-life assembly of gut microbiota in passerine chicks via nests. Microbiome. 2020;8:1–13. 32917256 10.1186/s40168-020-00896-9 PMC7488855

[96] Somers SE, Davidson GL, Johnson CN, Reichert MS, Crane JM, Ross RP, Stanton C, Quinn JL. Individual variation in the avian gut microbiota: The influence of host state and environmental heterogeneity. Mol Ecol. 2023;32(12):3322–39. 10.1111/mec.16919 36906957

[97] Diez‐Méndez D, Bodawatta KH, Freiberga I, Klečková I, Jønsson KA, Poulsen M, Sam K. Indirect maternal effects via nest microbiome composition drive gut colonization in altricial chicks. Mol Ecol. 2023;32(13):3657–71. 10.1111/mec.16959 37096441

[98] Pereira H, Chakarov N, Hoffman JI, Rinaud T, Ottensmann M, Gladow KP, et al. Early-life factors shaping the gut microbiota of Common buzzard nestlings. Anim Microbiome. 2024;6(1):27. 38745254 10.1186/s42523-024-00313-8 PMC11092241

[99] Mota-Rojas D, Marcet-Rius M, Domínguez-Oliva A, Buenhombre J, Daza-Cardona EA, Lezama-García K, et al. Parental behavior and newborn attachment in birds: life history traits and endocrine responses. Front Psychol. 2023;14:1183554. 37599744 10.3389/fpsyg.2023.1183554 PMC10434784

[100] Grond K, Lanctot RB, Jumpponen A, Sandercock BK. Recruitment and establishment of the gut microbiome in arctic shorebirds. FEMS Microbiol Ecol. 2017;93(12):fix142. 10.1093/femsec/fix142 29069418

