# Supplementary material for: The gut microbiota of three avian species living in sympatry
Source: BMC Ecol Evol. 2024 Nov 21;24:144. doi: 10.1186/s12862-024-02329-9 (PMC11580620; doi:10.1186/s12862-024-02329-9)
Supplement: Supplementary file 1 — Additional file 1. Appendix A. Supplementary tables and figures. [file 12862_2024_2329_MOESM1_ESM.pdf]

Supplementary tables and figures

Supplementary tables and figures
- Figure S1. Rarefaction plots
- Figure S2. PERMANOVA and Bayesian regression models depicting compositional differences between species (A to D). LMM results of microbiota diversity between the three plover species (E-F)
- Figure S3. Effect size estimates (points) and corresponding 95% credible intervals (horizontal lines) derived from Bayesian regression (brms) models with pairwise Bray-Curtis dissimilarities and Weighted UniFrac distances as response variables - subset by age
- Figure S4. Effect size estimates (points) and corresponding 95% credible intervals (horizontal lines) derived from Bayesian regression (brms) models with pairwise Bray-Curtis dissimilarities and Weighted UniFrac distances as response variables - combined dataset
- Figure S5. Regression plots depicting spacial distance between individuals and microbiota Bray-Curtis dissimilarities as obtained by a Mantel correlation test
- Figure S6. Differential abundant taxa - combined dataset
- Figure S7. Effect of species on microbiota alpha diversity (N° of observed ASVs) as obtained from a LMM.
- Table S1. Summary of final ASV table
  - Tabel S1.1. Summary of final reads per sample
- Table S2. Summary of raw ASV table
  - Tabel S2.1. Summary of raw reads per sample
  - Table S2.2. Summary of ASV table after removing contaminants ("decontam")
  - Table S2.3. Summary of ASV table after filtering steps
  - Table S2.4. Summary of rarefied ASV table
- Table S3. Relative abundances (%) of the most abundant taxonomic Phyla
- Table S4. Relative abundances (%) of the most abundant taxonomic families
- Table S5. Shannon diversity index LMM fit
  - Table S5.1. Bootstrap results - Shannon diversity
- Table S6. Faith phylogenetic diversity LMM fit
  - Table S6.1. Bootstrap results - Faith PD
- Table S7. N° of observed ASVs LMM fit
  - Table S7.1. Bootstrap results - N° of observed ASVs
- Table S8. Shannon diversity index LMM fit - Adutls only
  - Table S8.1. Bootstrap results - Adults only Shannon diversity
- Table S9. Faith phylogenetic diversity LMM fit - Adults only
  - Table S9.1. Bootstrap results - Adults only Faith PD
- Table S10. N° of observed ASVs LMM fit - Adults only
  - Table S10.1. Bootstrap results - Adults only n° of observed ASVs
- Table S11. Shannon diversity index LMM fit - Juveniles only
  - Table S11.1. Bootstrap results - Juveniles only Shannon diversity
- Table S12. Faith phylogenetic diversity LMM fit - Juveniles only
  - Table S12.1. Bootstrap results - Juveniles only Faith PD
- Table S13. N° of observed ASVs LMM fit - Juveniles only
  - Table S13.1. Bootstrap results - Juveniles only n° of ASVs
- Table S14. PERMANOVA outcomes for both Bray-Curtis and Weighted UniFrac
  - Table 14.1. Permutation test for homogeneity of multivariate dispersions
- Table S15. PERMANOVA outcomes for both Bray-Curtis and Weighted UniFrac, focusing on models that exclusively include adults
  - Table 15.1. Permutation test for homogeneity of multivariate dispersions - Adults
- Table S16. PERMANOVA outcomes for both Bray-Curtis and Weighted UniFrac, focusing on models that exclusively include juveniles
  - Table 16.1. Permutation test for homogeneity of multivariate dispersions - Juveniles
- Table S17. Bayesian model results for Bray-Curtis dissimilarities
- Table S18. Bayesian model results for Weighted UniFrac distances
- Table S19. Bayesian model results for Bray-Curtis dissimilarities - Adults
- Table S20. Bayesian model results for Weighted UniFrac distances - Adults
- Table S21. Bayesian model results for Bray-Curtis dissimilarities - Juveniles
- Table S22. Bayesian model results for Weighted UniFrac distances - Juveniles

Figure S1. Rarefaction plots

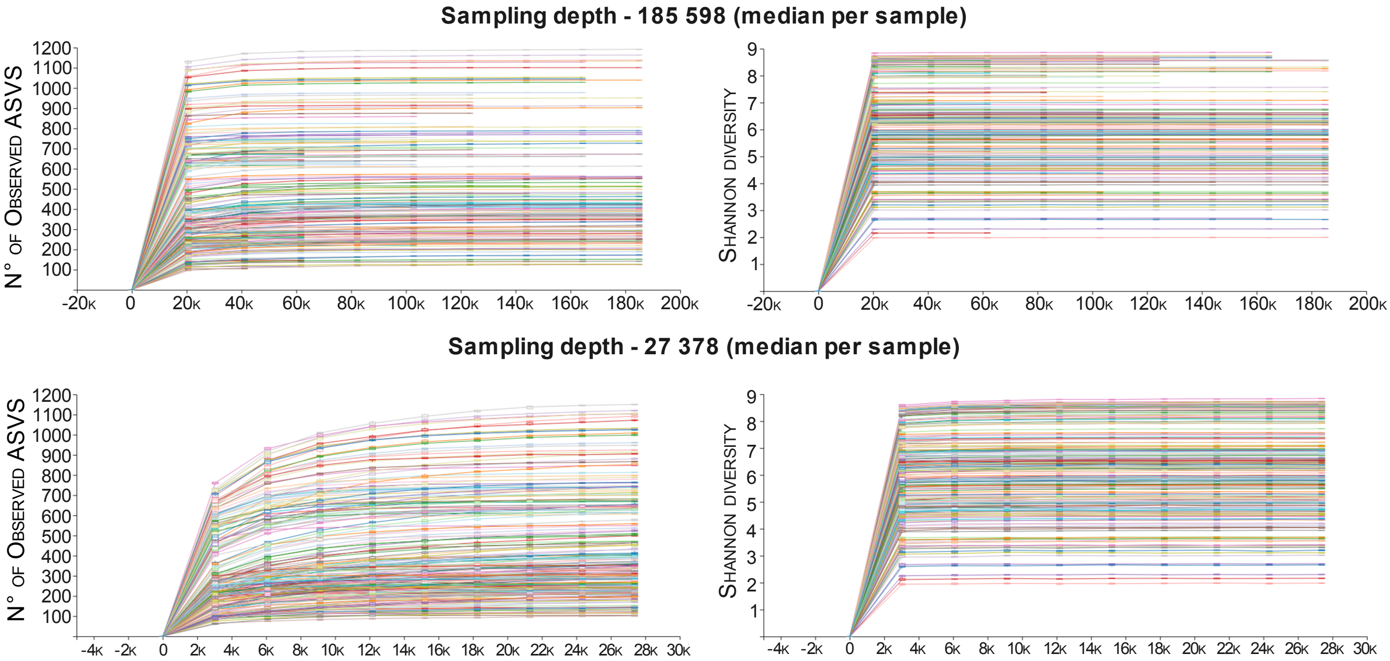

Rarefaction curves were generated for two alpha diversity metrics—number of observed ASVs and Shannon diversity—at two distinct sampling depths: the median sampling depth (185,598) and the sampling depth corresponding to the sample with the lowest number of reads (27,378).

Figure S2. PERMANOVA and Bayesian regression models depicting compositional differences between species (A to D). LMM results of microbiota diversity between the three plover species (E-F)

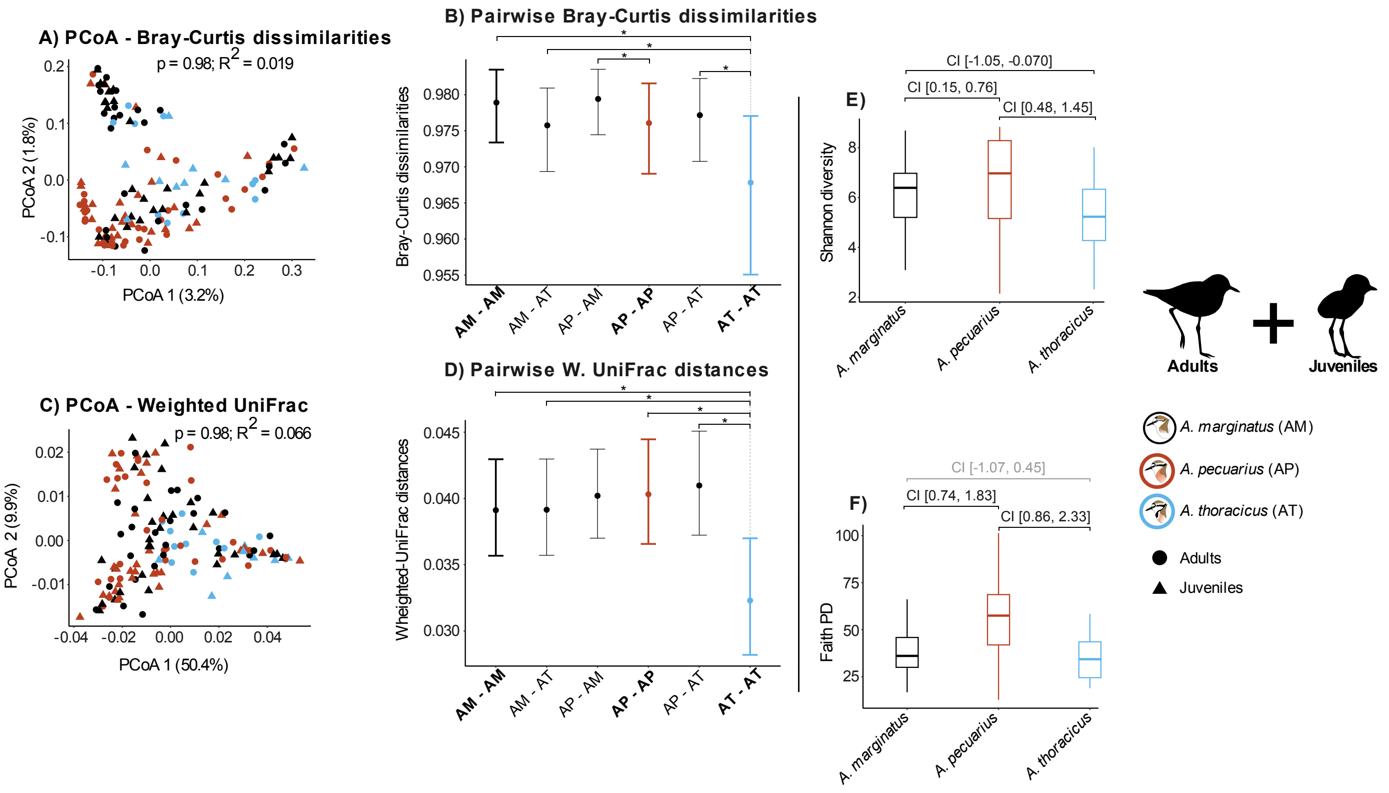

Figure S3. Effect size estimates (points) and corresponding 95% credible intervals (horizontal lines) derived from Bayesian regression (brms) models with pairwise Bray-Curtis dissimilarities and Weighted UniFrac distances as response variables - subset by age

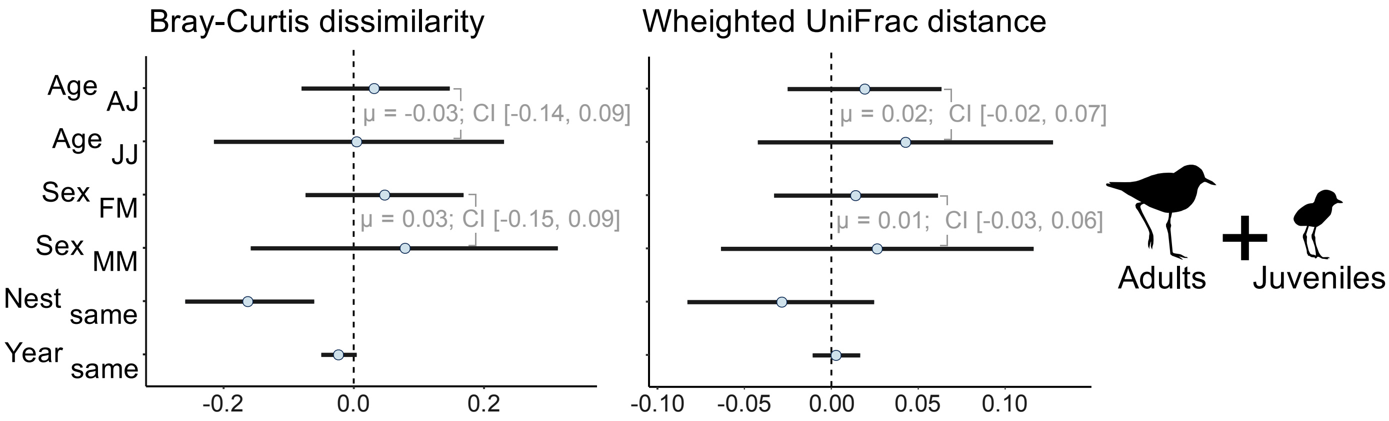

Figure S4. Effect size estimates (points) and corresponding 95% credible intervals (horizontal lines) derived from Bayesian regression (brms) models with pairwise Bray-Curtis dissimilarities and Weighted UniFrac distances as response variables - combined dataset

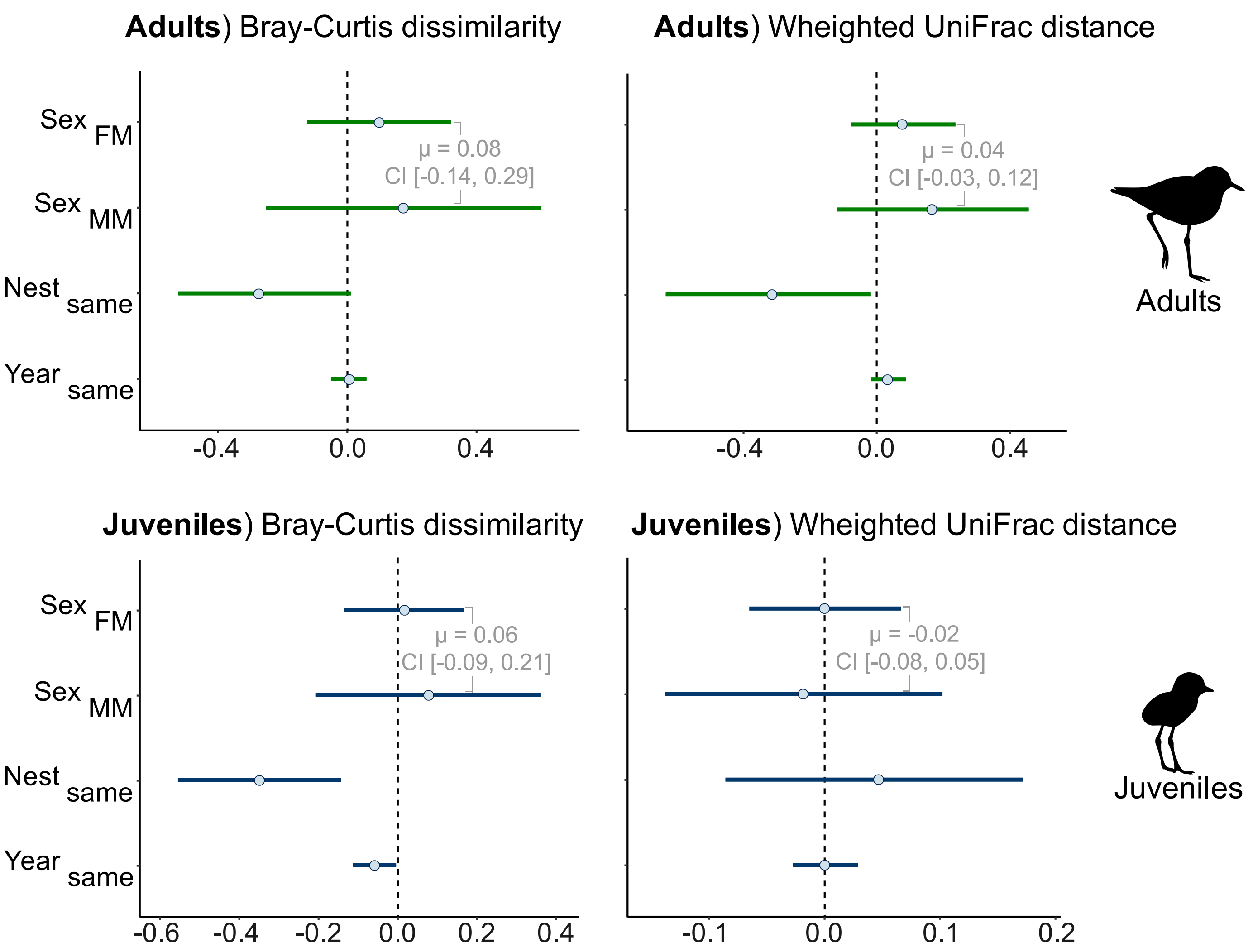

Figure S5. Regression plots depicting spacial distance between individuals and microbiota Bray-Curtis dissimilarities as obtained by a Mantel correlation test

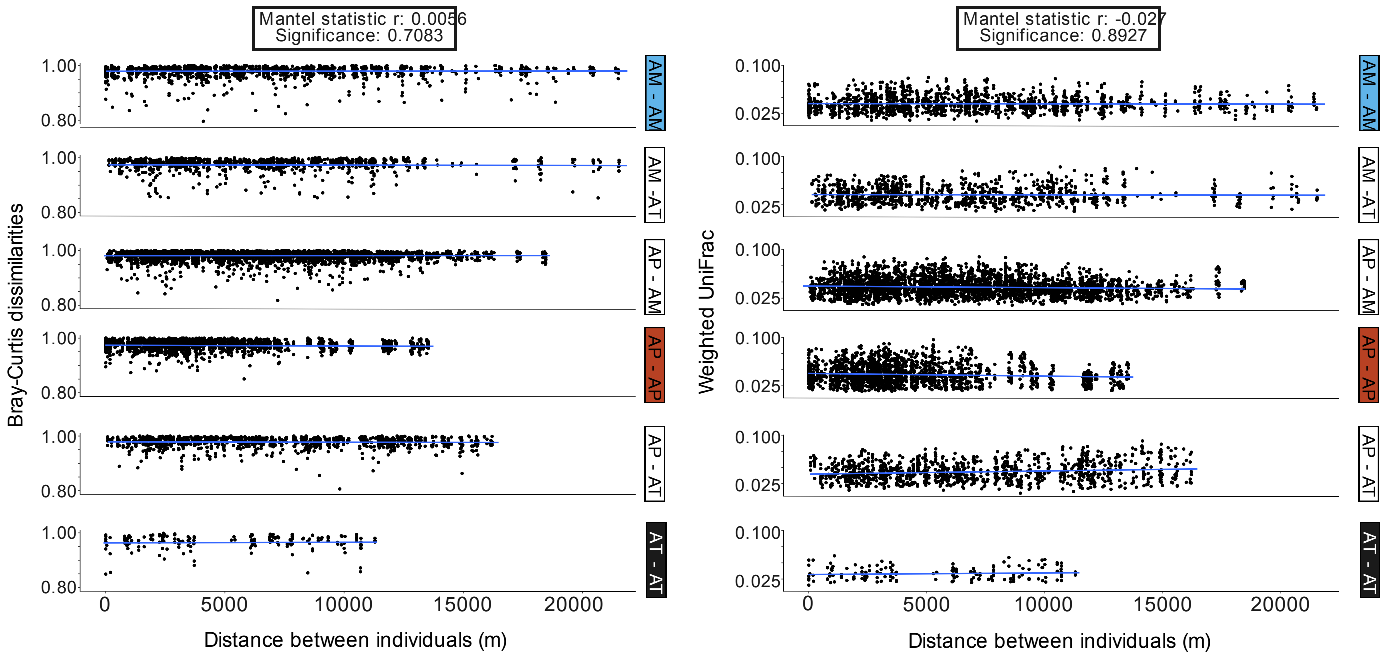

Figure S6. Differential abundant taxa - combined dataset

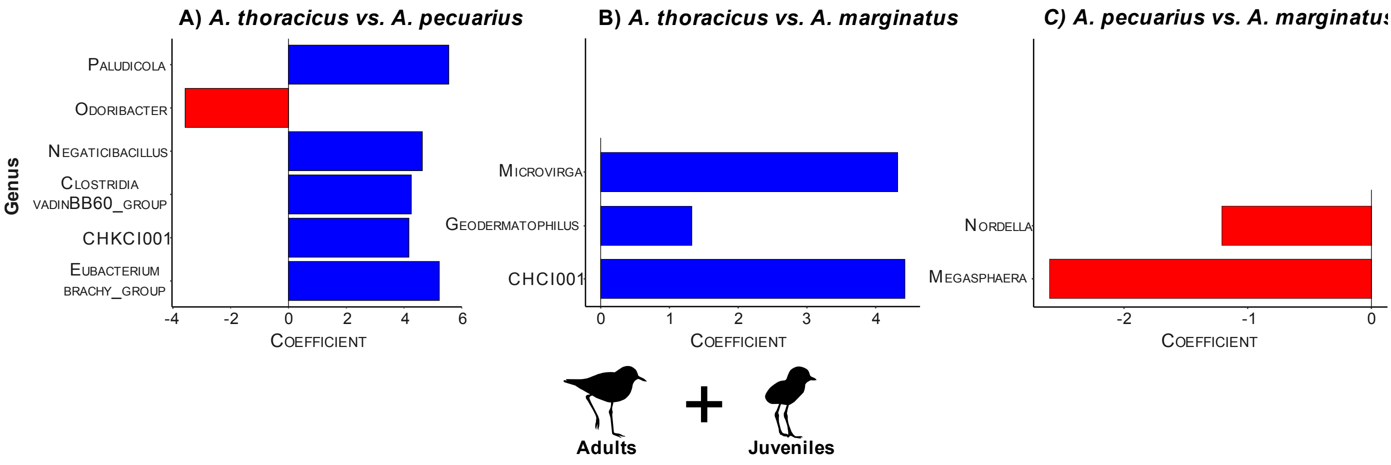

Figure S7. Effect of species on microbiota alpha diversity (N° of observed ASVs) as obtained from a LMM.

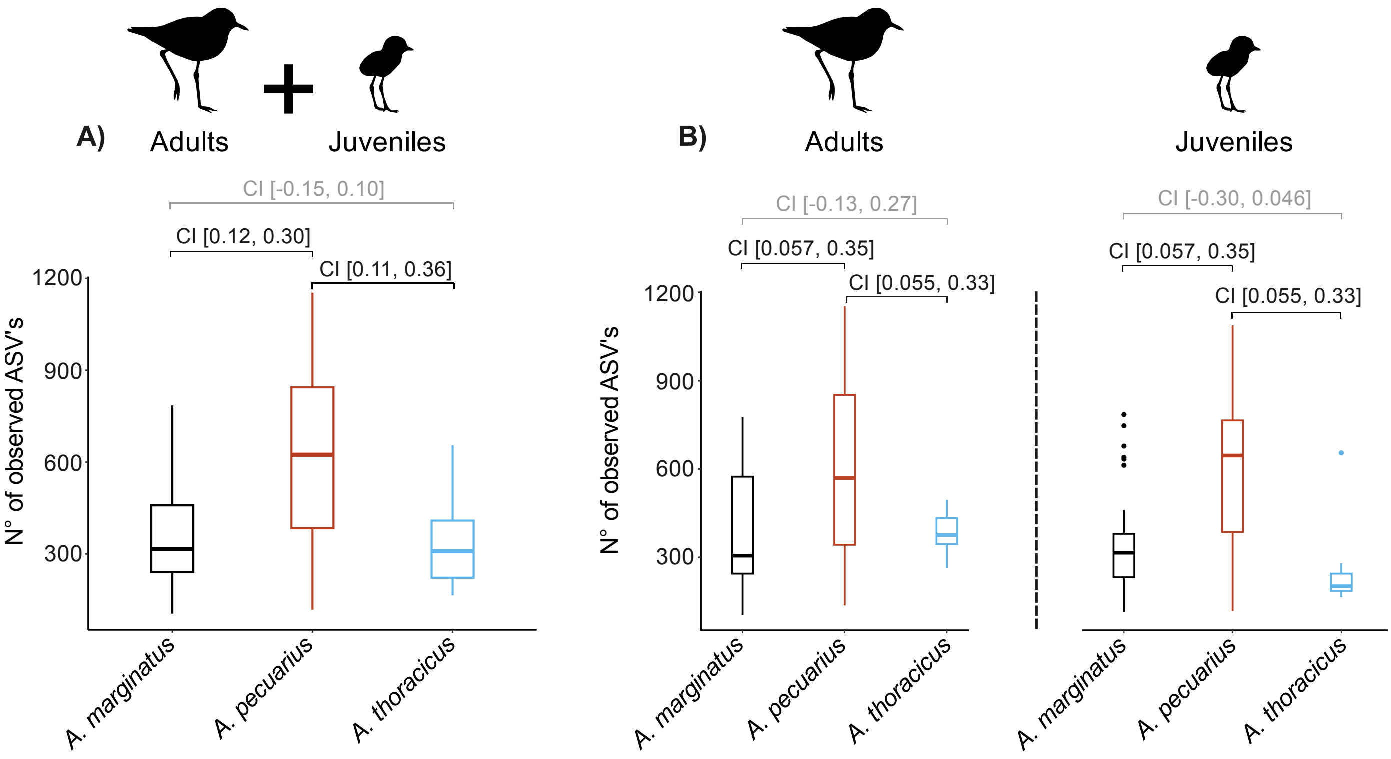

Table S1. Summary of final ASV table

	Sample
**Number of samples**	136
**Number of features (ASVs)**	28,278
**Total frequency**	30,186,364

Tabel S1.1. Summary of final reads per sample

	Frequency
**Minimum frequency**	27,378.0
**1st quartile**	85,801.25
**Median frequency**	166,170.0
**3rd quartile**	330,019.75
**Maximum frequency**	662,093.0
**Mean frequency**	221,958.56

Table S2. Summary of raw ASV table

	Sample
**Number of samples**	209*
**Number of features (ASVs)**	516,604
**Total frequency**	74,293,947

Tabel S2.1. Summary of raw reads per sample

	Frequency
**Minimum frequency**	79,221.0
**1st quartile**	210,082.0
**Median frequency**	347,637.0
**3rd quartile**	469,765.0
**Maximum frequency**	878,615.0
**Mean frequency**	355,473.43

Table S2.2. Summary of ASV table after removing contaminants ("decontam")

	Sample
**Number of samples**	209
**Number of features (ASVs)**	510,662
**Total frequency**	71,104,135

Table S2.3. Summary of ASV table after filtering steps

	Sample
**Number of samples**	201*
**Number of features (ASVs)**	29,935
**Total frequency**	46,603,613

Filtering steps included: taxonomic filtering, removal of singletons, exclusion of samples with fewer than 500 reads, filtering of contaminants identified in the positive control and removal of positive and negative controls.

Table S2.4. Summary of rarefied ASV table

	Sample
**Number of samples**	201*
**Number of features (ASVs)**	29,811
**Total frequency**	5,502,978

*From the initial data-set, individuals meeting the following criteria were excluded:

- Those with more than one sampling point (a total of 24 individuals; reserved for future studies);
- Individuals for whom sex determination was unsuccessful (molecular sexing failed);
- Only nests with multiple individuals were retained.

As a result, this study included a final number of 136 individuals.

Table S3. Relative abundances (%) of the most abundant taxonomic Phyla

Species	Phylum	Mean_abundance	SD	Core taxa
A. pecuarius	Firmicutes	42.89	18.34	Yes
A. pecuarius	Proteobacteria	19.80	12.76	Yes
A. pecuarius	Bacteroidota	12.80	8.42	Yes
A. pecuarius	Fusobacteriota	8.75	12.24	Yes
A. pecuarius	Actinobacteriota	6.71	7.00	Yes
A. pecuarius	Acidobacteriota	1.35	2.12	
A. pecuarius	Cyanobacteria	1.29	4.48	
A. pecuarius	Desulfobacterota	1.11	0.95	Yes
A. pecuarius	Gemmatimonadota	1.08	1.68	
A. pecuarius	Patescibacteria	0.70	1.06	
A. pecuarius	Deferribacterota	0.64	1.28	
A. pecuarius	Campylobacterota	0.59	1.12	Yes
A. marginatus	Firmicutes	45.10	17.58	Yes

Species	Phylum	Mean_abundance	SD	Core taxa
A. marginatus	Proteobacteria	19.58	15.73	Yes
A. marginatus	Bacteroidota	13.82	9.64	Yes
A. marginatus	Fusobacteriota	9.43	10.81	Yes
A. marginatus	Actinobacteriota	4.74	5.03	Yes
A. marginatus	Campylobacterota	1.81	5.44	
A. marginatus	Desulfobacterota	1.49	1.57	Yes
A. thoracicus	Firmicutes	53.11	20.73	Yes
A. thoracicus	Proteobacteria	13.13	17.28	Yes
A. thoracicus	Fusobacteriota	12.63	13.48	Yes
A. thoracicus	Bacteroidota	12.39	11.93	Yes
A. thoracicus	Actinobacteriota	3.74	7.13	Yes

Table S4. Relative abundances (%) of the most abundant taxonomic families

Species	Family	Mean_abundance	SD	Core taxa
A. pecuarius	Catellicoccaceae	9.05	15.21	
A. pecuarius	Fusobacteriaceae	8.74	12.28	Yes
A. pecuarius	Lachnospiraceae	8.61	6.94	Yes
A. pecuarius	Bacteroidaceae	5.40	7.14	Yes
A. pecuarius	Lactobacillaceae	4.24	3.97	Yes
A. pecuarius	Enterobacteriaceae	3.69	4.21	Yes
A. pecuarius	Ruminococcaceae	3.44	3.94	Yes
A. pecuarius	Oscillospiraceae	2.39	4.17	Yes
A. pecuarius	Peptostreptococcaceae	2.38	4.34	Yes
A. pecuarius	Enterococcaceae	2.30	8.68	Yes
A. pecuarius	Muribaculaceae	2.16	2.34	Yes
A. pecuarius	Pseudomonadaceae	1.88	2.63	
A. pecuarius	Prevotellaceae	1.86	1.54	Yes
A. pecuarius	Butyricicoccaceae	1.83	3.92	Yes
A. pecuarius	Bifidobacteriaceae	1.80	2.97	
A. pecuarius	Clostridiaceae	1.74	3.13	Yes
A. pecuarius	Erysipelotrichaceae	1.29	2.03	Yes
A. pecuarius	Moraxellaceae	1.18	2.86	
A. pecuarius	Rikenellaceae	1.15	2.39	Yes

Species	Family	Mean_abundance	SD	Core taxa
A. marginatus	Catellicoccaceae	11.74	18.95	
A. marginatus	Fusobacteriaceae	9.34	10.83	Yes
A. marginatus	Bacteroidaceae	6.36	7.75	Yes
A. marginatus	Lachnospiraceae	5.88	4.53	Yes
A. marginatus	Peptostreptococcaceae	4.67	8.01	Yes
A. marginatus	Lactobacillaceae	3.67	3.33	Yes
A. marginatus	Ruminococcaceae	3.35	3.59	Yes
A. marginatus	Enterobacteriaceae	3.15	4.41	Yes
A. marginatus	Prevotellaceae	2.47	2.81	Yes
A. marginatus	Moraxellaceae	2.30	4.91	
A. marginatus	Oscillospiraceae	2.15	2.72	Yes
A. marginatus	Enterococcaceae	1.87	5.23	
A. marginatus	Vibrionaceae	1.74	6.16	
A. marginatus	Rhodobacteraceae	1.70	2.56	
A. marginatus	Muribaculaceae	1.67	1.61	Yes
A. marginatus	Clostridiaceae	1.66	2.43	Yes
A. marginatus	Erysipelotrichaceae	1.62	1.73	Yes
A. marginatus	Aeromonadaceae	1.49	2.64	
A. marginatus	Xanthomonadaceae	1.31	2.09	
A. marginatus	Desulfovibrionaceae	1.30	1.59	Yes
A. marginatus	Butyricicoccaceae	1.27	2.54	Yes
A. marginatus	Bacillaceae	1.20	4.96	
A. marginatus	Helicobacteraceae	1.05	3.79	
A. marginatus	Rikenellaceae	1.00	0.86	Yes
A. thoracicus	Catellicoccaceae	14.53	25.70	
A. thoracicus	Fusobacteriaceae	12.63	13.49	Yes
A. thoracicus	Lachnospiraceae	9.72	8.38	Yes
A. thoracicus	Bacteroidaceae	7.64	8.88	Yes
A. thoracicus	Ruminococcaceae	5.47	5.91	Yes
A. thoracicus	Peptostreptococcaceae	4.45	8.30	
A. thoracicus	Butyricicoccaceae	4.17	6.22	
A. thoracicus	Moraxellaceae	3.02	7.85	
A. thoracicus	Oscillospiraceae	2.62	2.72	Yes
A. thoracicus	Enterobacteriaceae	2.40	5.70	Yes
A. thoracicus	Erysipelotrichaceae	2.01	2.63	Yes
A. thoracicus	Beijerinckiaceae	1.99	11.91	
A. thoracicus	Enterococcaceae	1.64	3.19	
A. thoracicus	Family_XI	1.48	3.86	

Species	Family	Mean_abundance	SD	Core taxa
A. thoracicus	Clostridiaceae	1.40	3.23	
A. thoracicus	Desulfovibrionaceae	1.33	1.86	
A. thoracicus	Shewanellaceae	1.23	6.65	
A. thoracicus	Deferribacteraceae	1.22	2.73	
A. thoracicus	Prevotellaceae	1.21	2.01	
A. thoracicus	Tannerellaceae	1.02	1.92	
A. thoracicus	Clostridia_vadinBB60_group	0.93	1.90	
A. thoracicus	Corynebacteriaceae	0.90	3.72	
A. thoracicus	Lactobacillaceae	0.90	1.51	Yes

Table S5. Shannon diversity index LMM fit

**Random effects:**					
**Groups**	**Name**	**Variance**	**Std.Dev.**		
**nest**	(Intercept)	0	0		
**Residual**		0.9199	0.9591		
**Fixed effects:**					
	**Estimate**	**Std. Error**	**df**	**t value**	**Pr(>|t|)**
**(Intercept)**	-0.03034	0.20902	130	-0.145	0.8848
**species Apecuarius**	0.38855	0.18482	130	2.102	0.0375*
**species Athoracicus**	-0.57568	0.25871	130	-2.225	0.0278*
**sexM**	0.02584	0.17282	130	0.15	0.8814
**ageJ**	-0.18973	0.16535	130	-1.147	0.2533
**year2022**	0.0662	0.1885	130	0.351	0.726
	**R2 (marginal)**	**R2 (conditional)**			
	0.1103745	0.1103745			

Table S5.1. Bootstrap results - Shannon diversity

**Number of resamples**	10000		
**Bootstrap type**	parametric		
Shannon			
	**Estimate**	**Lower 95%**	**Upper 95%**

(Intercept)	-0.0303	-0.449	0.379
**A. pecuarius - A. marginatus**	**0.389**	**0.0145**	**0.756***
**A. thoracicus - A.marginatus**	**-0.576**	**-1.05**	**-0.0697***
**A. pecuarius - A. thoracicus**	**0.964**	**0.484**	**1.45***
Male - Female	0.0258	-0.318	0.363
Juveniles - Adults	-0.19	-0.507	0.134
2022 - 2021	0.0662	-0.305	0.435

Table S6. Faith phylogenetic diversity LMM fit

**Random effects:**					
**Groups**	**Name**	**Variance**	**Std.Dev.**		
**nest**	(Intercept)	0.1208	0.3475		
**Residual**		1.7532	1.3241		
**Fixed effects:**					
	**Estimate**	**Std. Error**	**df**	**t value**	**Pr(>|t|)**
**(Intercept)**	6.0099	0.3065	77.0815	19.608	<2.00E-16*
**species Apecuarius**	1.2851	0.2782	39.0447	4.62	4.11E-05*
**species Athoracicus**	-0.3103	0.3883	41.4713	-0.799	0.429
**sexM**	0.1761	0.2445	125.4179	0.72	0.473
**ageJ**	-0.1549	0.2303	95.1482	-0.673	0.503
**year2022**	0.2778	0.2833	40.2536	0.981	0.333
	**R2 (marginal)**	**R2 (conditional)**			
	0.2059659	0.2571445			

Table S6.1. Bootstrap results - Faith PD

**Bootstrap type**	parametric		
**Number of resamples**	10000		
**Faith PD**			
	**Estimate**	**Lower 95%**	**Upper 95%**
(Intercept)	6.01	5.41	6.61
**A. pecuarius - A. marginatus**	**1.29**	**0.735**	**1.83***
A. thoracicus - A.marginatus	-0.31	-1.07	0.454

**A. pecuarius - A. thoracicus**	**1.6**	**0.858**	**2.33***
Male - Female	0.176	-0.306	0.659
Juveniles - Adults	-0.155	-0.611	0.303
2022 - 2021	0.278	-0.282	0.833

Table S7. N° of observed ASVs LMM fit

**Random effects:**					
**Groups**	**Name**	**Variance**	**Std.Dev.**		
**nest**	(Intercept)	0.001851	0.04303		
**Residual**		0.051841	0.22769		
**Fixed effects:**					
	**Estimate**	**Std. Error**	**df**	**t value**	**Pr(>|t|)**
**(Intercept)**	2.48965	0.05127	76.66176	48.563	<2.00E-16*
**species Apecuarius**	0.21	0.04598	38.1175	4.567	5.06E-05*
**species Athoracicus**	-0.02443	0.06427	40.72999	-0.38	0.706
**sexM**	0.02054	0.04159	126.81443	0.494	0.622
**ageJ**	-0.02182	0.03945	96.61883	-0.553	0.581
**year2022**	0.07048	0.04687	39.30007	1.504	0.141
	**R2 (marginal)**	**R2 (conditional)**			
	0.1810203	0.2092595			

Table S7.1. Bootstrap results - N° of observed ASVs

**Bootstrap type**	parametric		
**Number of resamples**	10000		
**N° of ASVs**			
	**Estimate**	**Lower 95%**	**Upper 95%**
(Intercept)	2.49	2.39	2.59
**A. pecuarius - A. marginatus**	**0.21**	**0.12**	**0.301***
A. thoracicus - A.marginatus	-0.0244	-0.148	0.101
**A. pecuarius - A. thoracicus**	**0.234**	**0.111**	**0.358***
Male - Female	0.0205	-0.0607	0.102
Juveniles - Adults	-0.0218	-0.0988	0.0555

2022 - 2021	0.0705	-0.0215	0.163

Table S8. Shannon diversity index LMM fit - Adutls only

**Random effects:**					
**Groups**	**Name**	**Variance**	**Std.Dev.**		
**nest**	**(Intercept)**	0.4035	0.6352		
**Residual**		0.52	0.7211		
**Fixed effects:**					
	**Estimate**	**Std. Error**	**df**	**t value**	**Pr(>|t|)**
**(Intercept)**	0.03557	0.27437	52.80645	0.13	0.897
**species Apecuarius**	0.17417	0.29863	45.79982	0.583	0.563
**species Athoracicus**	-0.62381	0.3982	40.06428	-1.567	0.125
**sexM**	-0.0966	0.2284	31.90288	-0.423	0.675
**year2022**	0.36891	0.29154	41.38485	1.265	0.213
	**R2 (marginal)**	**R2 (conditional)**			
	0.09955472	0.492967			

Table S8.1. Bootstrap results - Adults only Shannon diversity

**Bootstrap type**	parametric		
**Number of resamples**	10000		
**Shannon**			
	**Estimate**	**Lower 95%**	**Upper 95%**
(Intercept)	0.0356	-0.509	0.583
A. pecuarius - A. marginatus	0.174	-0.416	0.765
A. thoracicus - A.marginatus	-0.624	-1.4	0.151
**A. pecuarius - A. thoracicus**	**0.798**	**0.0324**	**1.55***
Male - Female	-0.0966	-0.554	0.361
2022 - 2021	0.369	-0.203	0.945

Table S9. Faith phylogenetic diversity LMM fit - Adults only

**Random effects:**					
**Groups**	**Name**	**Variance**	**Std.Dev.**		
**nest**	**(Intercept)**	0.5465	0.7392		
**Residual**		0.4435	0.666		
**Fixed effects:**					
	**Estimate**	**Std. Error**	**df**	**t value**	**Pr(>|t|)**
**(Intercept)**	-0.49156	0.28456	51.67143	-1.727	0.09006
**species Apecuarius**	0.91201	0.3127	44.33645	2.917	0.00553*
**species Athoracicus**	0.05964	0.42002	38.88301	0.142	0.88782
**sexM**	0.04951	0.22031	24.63836	0.225	0.82405
**year2022**	0.2641	0.307	40.05682	0.86	0.39476
	**R2 (marginal)**	**R2 (conditional)**			
	0.1663308	0.62651			

Table S9.1. Bootstrap results - Adults only Faith PD

**Bootstrap type**	**parametric**		
**Number of resamples**	10000		
Faith PD			
	**Estimate**	**Lower 95%**	**Upper 95%**
(Intercept)	-0.492	-1.06	0.0641
**A. pecuarius - A. marginatus**	**0.912**	**0.299**	**1.53***
A. thoracicus - A.marginatus	0.0596	-0.754	0.884
**A. pecuarius - A. thoracicus**	**0.852**	**0.0525**	**1.66***
Male - Female	0.0495	-0.396	0.497
2022 - 2021	0.264	-0.34	0.873

Table S10. N° of observed ASVs LMM fit - Adults only

**Random effects:**					
**Groups**	**Name**	**Variance**	**Std.Dev.**		
**nest**	**(Intercept)**	0.4798	0.6927		
**Residual**		0.4757	0.6897		
**Fixed effects:**					

	**Estimate**	**Std. Error**	**df**	**t value**	**Pr(>|t|)**
**(Intercept)**	2.46158	0.06986	49.56185	35.238	**<2e-16*****
**species Apecuarius**	0.20454	0.07585	40.48504	2.697	**0.0102***
**species Athoracicus**	0.06808	0.10094	33.68745	0.674	0.5046
**sexM**	0.01386	0.05914	26.80535	0.234	0.8165
**year2022**	0.11758	0.07393	35.22181	1.59	0.1207
	**R2 (marginal)**	**R2(conditional)**			
	0.142139	0.4899323			

Table S10.1. Bootstrap results - Adults only n° of observed ASVs

**Bootstrap type**	parametric		
**Number of resamples**	10000		
**N° of ASVs**			
	**Estimate**	**Lower 95%**	**Upper 95%**
(Intercept)	2.46	2.32	2.6
**A. pecuarius - A. marginatus**	**0.205**	**0.0567**	**0.354***
A. thoracicus - A.marginatus	0.0681	-0.131	0.268
A. pecuarius - A. thoracicus	0.136	-0.055	0.33
Male - Female	0.0139	-0.103	0.133
2022 - 2021	0.118	-0.0287	0.262

Table S11. Shannon diversity index LMM fit - Juveniles only

**Random effects:**					
**Groups**	**Name**	**Variance**	**Std.Dev.**		
**nest**	**(Intercept)**	0.006963	0.08344		
**Residual**		0.908452	0.95313		
**Fixed effects:**					
	**Estimate**	**Std. Error**	**df**	**t value**	**Pr(>|t|)**
**(Intercept)**	-0.3325	0.2583	32.5243	-1.287	0.2072
**species Apecuarius**	0.5951	0.2523	26.8008	2.359	0.0259
**species Athoracicus**	-0.596	0.3748	24.5127	-1.59	0.1246

**sexM**	0.1721	0.2331	65.8517	0.738	0.463
**year2022**	-0.1546	0.2683	27.5157	-0.576	0.5691
	**R2 (marginal)**	**R2 conditional)**			
	0.1719504	0.1782489			

Table S11.1. Bootstrap results - Juveniles only Shannon diversity

**Bootstrap type**	parametric		
Number of resamples	10000		
Shannon			
	**Estimate**	**Lower 95%**	**Upper 95%**
(Intercept)	-0.332	-0.838	0.179
**A. pecuarius - A. marginatus**	**0.595**	**0.0961**	**1.09***
A. thoracicus - A.marginatus	-0.596	-1.34	0.145
**A. pecuarius - A. thoracicus**	**1.19**	**0.474**	**1.91***
Male - Female	0.172	-0.288	0.627
2022 - 2021	-0.155	-0.685	0.374

Table S12. Faith phylogenetic diversity LMM fit - Juveniles only

**Random effects:**					
**Groups**	**Name**	**Variance**	**Std.Dev.**		
**nest**	**(Intercept)**	0	0		
**Residual**		1.668	1.291		
**Fixed effects:**					
	**Estimate**	**Std. Error**	**df**	**t value**	**Pr(>|t|)**
**(Intercept)**	5.896	0.348	66	16.942	<2.00E-16
**species Apecuarius**	1.3298	0.3397	66	3.914	0.000217
**species Athoracicus**	-0.846	0.5046	66	-1.677	0.098355
**sexM**	0.2281	0.3147	66	0.725	0.471161
**year2022**	0.1761	0.3613	66	0.487	0.627682
	**R2 (marginal)**	**R2 conditional)**			

	0.2844682	0.2844682			

Table S12.1. Bootstrap results - Juveniles only Faith PD

**Bootstrap type**	parametric		
**Number of resamples**	10000		
**Faith PD**			
	**Estimate**	**Lower 95%**	**Upper 95%**
(Intercept)	5.9	5.2	6.6
**A. pecuarius - A. marginatus**	**1.33**	**0.653**	**2***
A. thoracicus - A.marginatus	-0.846	-1.84	0.138
**A. pecuarius - A. thoracicus**	**2.18**	**1.21**	**3.14***
Male - Female	0.228	-0.4	0.853
2022 - 2021	0.176	-0.545	0.888

Table S13. N° of observed ASVs LMM fit - Juveniles only

**Random effects:**					
**Groups**	**Name**	**Variance**	**Std.Dev.**		
**nest**	**(Intercept)**	0	0		
**Residual**		0.04958	0.2227		
**Fixed effects:**					
	**Estimate**	**Std. Error**	**df**	**t value**	**Pr(>|t|)**
**(Intercept)**	2.48407	0.06	66	41.399	<2.00E-16***
**species Apecuarius**	0.21979	0.05858	66	3.752	0.000372***
**species Athoracicus**	-0.12625	0.087	66	-1.451	0.151445
**sexM**	0.02501	0.05426	66	0.461	0.646358
**year2022**	0.03192	0.0623	66	0.512	0.610115
	**R2 (marginal)**	**R2 (conditional)**			
	0.256622	0.256622			

Table S13.1. Bootstrap results - Juveniles only n° of ASVs

**Bootstrap type**	parametric		
**Number of resamples**	10000		
**N° of ASVs**			
	**Estimate**	**Lower 95%**	**Upper 95%**
(Intercept)	2.48	2.37	2.6
**A. pecuarius - A. marginatus**	**0.22**	**0.105**	**0.335***
A. thoracicus - A.marginatus	-0.126	-0.296	0.0456
**A. pecuarius - A. thoracicus**	**0.346**	**0.18**	**0.511***
Male - Female	0.025	-0.0823	0.132
2022 - 2021	0.0319	-0.0906	0.155

Table S14. PERMANOVA outcomes for both Bray-Curtis and Weighted UniFrac

**Response:**	Bray-Curtis dissimilarity				
**Blocks:**	with(metadata, nest)				
**Permutation:**	free				
**Number of permutations:**	10000				
	**Df**	**SumOfSqs**	**R2**	**F**	**Pr(>F)**
**species**	2	1.245	0.01932	1.3129	0.9756
**sex**	1	0.476	0.00739	1.0041	0.35756
**age**	1	0.511	0.00793	1.0779	0.05739
**year**	1	0.532	0.00825	1.1214	0.60224
**Residual**	130	61.652	0.95662		
**Total**	135	64.448	1		
**Response:**	Weighted UniFrac distances				
**Blocks:**	with(metadata, nest)				
**Permutation:**	free				
**Number of permutations:**	10000				
	**Df**	**SumOfSqs**	**R2**	**F**	**Pr(>F)**
**species**	2	0.008724	0.06643	4.7304	0.9808

**sex**	1	0.001159	0.00882	1.2565	0.177
**age**	1	0.000553	0.00421	0.5992	0.6562
**year**	1	0.000726	0.00553	0.7872	0.0109*
**Residual**	130	0.119879	0.91275		
**Total**	135	0.131337	1		

Table 14.1. Permutation test for homogeneity of multivariate dispersions

**Response:**	Bray-Curtis dissimilarity					
**Blocks:**	with(metadata, nest)					
**Permutation**	free					
**Number of permutations:**	10000					
	**Df**	**SumSq**	**MeanSq**	**F**	**N.Perm**	**Pr(>F)**
**Groups**	2	0.0060822	0.00304109	22.908	10000	1.00E-04***
**Residuals**	133	0.0176563	0.00013275			
**Pairwise comparisons:**						
	**A.marginatus**	**A.pecuarius**	**A. thoracicus**			
**A.marginatus**		1	1			
**A.pecuarius**	0.774292		1			
**A. thoracicus**	**1.40E-06*** **	**1.15E-08*** **				
**Response:**	Weighted UniFrac distances					
**Blocks:**	with(metadata, nest)					
**Permutation**	free					
**Number of permutations:**	10000					
	**Df**	**SumSq**	**MeanSq**	**F**	**N.Perm**	**Pr(>F)**
**Groups**	2	0.0006511	0.00032553	3.524	10000	1.00-04
**Residuals**	133	0.012286	0.00009238			
**Pairwise comparisons:**						
	**A.marginatus**	**A.pecuarius**	**A. thoracicus**			
**A.marginatus**		1	1			
**A.pecuarius**	0.451733		1			
**A. thoracicus**	**0.016428***	**0.015003***				

Note : Observed p-value below diagonal, permuted p-value above diagonal

Table S15. PERMANOVA outcomes for both Bray-Curtis and Weighted UniFrac, focusing on models that exclusively include adults

**Response:**	Bray-Curtis dissimilarity				
**Blocks:**	with(metadata_adults, nest)				
**Permutation:**	free				
**Number of permutations:**	10000				
	**Df**	**SumOfSqs**	**R2**	**F**	**Pr(>F)**
**species**	2	1.0701	0.03511	1.1298	0.241
**sex**	1	0.4757	0.01561	1.0045	0.2587
**year**	1	0.4964	0.01629	1.0482	0.9199
**Residual**	60	28.4128	0.93218		
**Total**	64	30.4799	1		
**Response:**	Weighted UniFrac distances				
**Blocks:**	with(metadata_adults, nest)				
**Permutation:**	free				
**Number of permutations:**	10000				
	**Df**	**SumOfSqs**	**R2**	**F**	**Pr(>F)**
**species**	2	0.002542	0.04294	1.3692	0.523
**sex**	1	0.00057	0.00963	0.6143	0.8151
**year**	1	0.0004	0.00676	0.431	0.7189
**Residual**	60	0.055704	0.94081		
**Total**	64	0.059209	1		

Table 15.1. Permutation test for homogeneity of multivariate dispersions - Adults

**Response:**	Bray-Curtis dissimilarity					
**Blocks:**	with(metadata, nest)					
**Permutation**	free					
**Number of permutations:**	10000					
	**Df**	**SumSq**	**MeanSq**	**F**	**N.Perm**	**Pr(>F)**
**Groups**	2	0.0071217	0.0035608	20.062	10000	1.00E-04**

**Residuals**	62	0.0110043	0.0001775			
**Pairwise comparisons:**						
	**A.marginatus**	**A. pecuarius**	**A.thoracicus**			
**A.marginatus**		1	1			
**A. pecuarius**	6.89E-01		1			
**A.thoracicus**	**7.51E-06*** **	**5.49E-06*** **				
**Response:**	Weighted UniFrac distances					
**Blocks:**	with(metadata, nest)					
**Permutation**	free					
**Number of permutations:**	10000					
	**Df**	**SumSq**	**MeanSq**	**F**	**N.Perm**	**Pr(>F)**
**Groups**	2	0.0009942	4.97E-04	6.0929	10000	1.00E-04**
**Residuals**	62	0.0050584	8.16E-05			
**Pairwise comparisons:**						
	**A.marginatus**	**A. pecuarius**	**A.thoracicus**			
**A.marginatus**		1	1			
**A. pecuarius**	0.2953976		1			
**A.thoracicus**	**0.0027114** **	**2.65E-03*** **				

Note : Observed p-value below diagonal, permuted p-value above diagonal

---

Table S16. PERMANOVA outcomes for both Bray-Curtis and Weighted UniFrac, focusing on models that exclusively include juveniles

**Response:**	*Bray-Curtis dissimilarity*				
**Blocks:**	*with(metadata_juv, nest)*				
**Permutation:**	*free*				
**Number of permutations:**	*10000*				
	**Df**	**SumOfSqs**	**R2**	**F**	**Pr(>F)**
**species**	2	1.178	0.03521	1.2421	0.8648
**sex**	1	0.455	0.0136	0.9597	0.8061
**year**	1	0.527	0.01576	1.1118	0.5111

**Residual**	66	31.294	0.93538		
**Total**	70	33.455	1		
**Response:**	*Weighted UniFrac distances*				
**Blocks:**	*with(metadata_juv, nest)*				
**Permutation:**	*free*				
**Number of permutations:**	*10000*				
	**Df**	**SumOfSqs**	**R2**	**F**	**Pr(>F)**
**species**	2	0.008872	0.12399	4.9143	0.2495
**sex**	1	0.001929	0.02696	2.137	0.2422
**year**	1	0.00094	0.01313	1.041	0.2217
**Residual**	66	0.059574	0.8326		
**Total**	70	0.071553	1		

Table 16.1. Permutation test for homogeneity of multivariate dispersions - Juveniles

**Response:**	*Bray-Curtis dissimilarity*					
**Blocks:**	*with(metadata, nest)*					
**Permutation**	*free*					
**Number of permutations:**	*10000*					
	**Df**	**SumSq**	**MeanSq**	**F**	**N.Perm**	**Pr(>F)**
**Groups**	2	0.0112517	0.0056258	39.535	10000	1.00E-04***
**Residuals**	68	0.0096766	0.0001423			
**Pairwise comparisons:**						
	A.marginatus	A. pecuarius	A.thoracicus			
**A.marginatus**		1	1			
**A. pecuarius**	9.18E-01		1			
**A.thoracicus**	7.66E-08***	4.27E-11***				
**Response:**	*Weighted UniFrac distances*					
**Blocks:**	*with(metadata, nest)*					
**Permutation**	*free*					
**Number of permutations:**	*10000*					
	**Df**	**SumSq**	**MeanSq**	**F**	**N.Perm**	**Pr(>F)**
**Groups**	2	0.0002147	0.00010733	0.9303	10000	1.00E-04***

**Residuals**	68	0.0078451	0.00011537			
**Pairwise comparisons:**						
	**A.marginatus**	**A. pecuarius**	**A.thoracicus**			
**A.marginatus**		1	1			
**A. pecuarius**	0.86979		1			
**A.thoracicus**	0.13201	0.25954				

Note : Observed p-value below diagonal, permuted p-value above diagonal

Table S17. Bayesian model results for Bray-Curtis dissimilarities

**Group-Level Effects:**							
**mmIDAIDB (Number of levels 65)**							
	**Estimate**	**Est.Error**	**l-95% CI**	**u-95% CI**	**Rhat**	**Bulk_ESS**	**Tail_ESS**
**sd(Intercept)**	0.65	0.04	0.58	0.74	1	5804	12883
**Population-Level Effects**							
	**Estimate**	**Est.Error**	**l-95% CI**	**u-95% CI**	**Rhat**	**Bulk_ESS**	**Tail_ESS**
**Intercept**	3.83	0.12	3.59	4.08	1	3193	7151
**AM-AM vs. AM-AT**	-0.14	0.09	-0.32	0.03	1	3647	7968
**AM-AM vs. AM-AP**	0.02	0.06	-0.1	0.15	1	3076	7058
**AM-AM vs. AP-AP**	-0.13	0.12	-0.37	0.11	1	2920	6426
**AM-AM vs. AP-AT**	-0.08	0.13	-0.33	0.16	1	3091	6593
**AM-AM vs. AT-AT**	-0.44	0.18	-0.79	-0.08	1	3608	8145
**A-A vs. A-J (age)**	0.03	0.06	-0.08	0.15	1	3120	6230
**A-A vs. J-J (age)**	0.01	0.11	-0.21	0.23	1	2990	5912
**F-F vs F-M (sex)**	0.05	0.06	-0.07	0.17	1	2926	7306
**F-F vs. M-M (sex)**	0.08	0.12	-0.16	0.31	1	2777	6856
**Nest (same nest)**	-0.16	0.05	-0.26	-0.06	1	69067	71755
**Year (same year)**	-0.02	0.01	-0.05	0	1	59919	68978
**Hypothesis testing: remaining comparisons**	**Estimate**	**Est.Error**	**l-95% CI**	**u-95% CI**			
**AP-AP vs. AP-AT**	-0.05	0.09	-0.22	0.13			
**AP-AP vs. AP-AM**	-0.16	0.06	-0.28	-0.03			
**AT-AT vs. AT-AP**	-0.35	0.1	-0.54	-0.17			
**AT-AT vs. AT-AM**	-0.29	0.1	-0.48	-0.1			
**AT-AT vs. AP-AP**	-0.31	0.18	-0.65	0.04			

J-J vs. AJ (age)	-0.03	0.06	-0.14	0.09			
M-M vs. F-M (sex)	0.03	0.06	-0.09	0.15			

AM (*Anarhynchus marginatus*); **AT** (*Anarhynchus thoracicus*); **AP** (*Anarhynchus pecuarius*); **F** (female); **M** (male)

AM-AM vs. AM-AT: Dissimilarities within *A. marginatus* (AM-AM) compared to between pairs of individuals from *A. marginatus* and *A. thoracicus* (AM-AT) (testing within vs. between species pairwise differences)
AM-AM vs. AM-AP: Dissimilarities within *A. marginatus* (AM-AM) compared to between pairs of individuals from *A. marginatus* and *A. pecuarius* (AM-AP) (testing within vs. between species pairwise differences)
AP-AP vs. AP-AT: Dissimilarities within *A. pecuarius* (AP-AP) compared to between pairs of individuals from *A. pecuarius* and *A. thoraciucs* (AP-AT) (testing within vs. between species pairwise differences)
AP-AP vs. AP-AM: Dissimilarities within *A. pecuarius* (AP-AP) compared to between pairs of individuals from *A. pecuarius* and *A. marginatus* (AP-AM) (testing within vs. between species pairwise differences)
AT-AT vs. AT-AP: Dissimilarities within *A. thoracicus* (AT-AT) compared to between pairs of individuals from *A. thoracicus* and *A. pecuarius* (AT-AP) (testing within vs. between species pairwise differences)
AT-AT vs. AT-AM: Dissimilarities within *A. thoracicus* (AT-AT) compared to between pairs of individuals from *A. thoracicus* and *A. marginatus* (AT-AM) (testing within vs. between species pairwise differences)
AM-AM vs. AP-AP: Dissimilarities within *A. marginatus* (AM-AM) compared to within *A. pecuarius* (AP-AP) (testing within species pairwise differences)
AM-AM vs. AT-AT: Dissimilarities within *A. marginatus* (AM-AM) compared to within *A. thoracicus* (AT-AT) (testing within species pairwise differences)
AT-AT vs. AP-AP: Dissimilarities within *A. thoracicus* (AT-AT) compared to within *A. pecuarius* (AP-AP) (testing within species pairwise differences)
F-F vs F-M (sex): Dissimilarity within females compared dissimilarities between sexes
F-F vs. M-M (sex): Dissimilarity within females compared to dissimilarities within males
A-A vs A-J (age): Dissimilarity within adults compared to dissimilarities between the two age groups
A-A vs. J-J (sex): Dissimilarity within adults compared to dissimilarities within juveniles
Nest-sharing: pairwise comparisons between individuals of same/different nests
Habitat-similarity: pairwise comparisons between individuals of same/different habitat

Table S18. Bayesian model results for Weighted UniFrac distances

**Group-Level Effects:**							
**mmIDAIDB (Number of levels 65)**							
	**Estimate**	**Est.Error**	**l-95% CI**	**u-95% CI**	**Rhat**	**Bulk_ESS**	**Tail_ESS**
**sd(Intercept)**	0.25	0.02	0.22	0.28	1	11509	22842
**Population-Level Effects**							
	**Estimate**	**Est.Error**	**l-95% CI**	**u-95% CI**	**Rhat**	**Bulk_ESS**	**Tail_ESS**
**Intercept**	-3.2	0.05	-3.3	-3.1	1	6336	13291
**AM-AM vs. AM-AT**	0	0.04	-0.07	0.07	1	7667	16356
**AM-AM vs. AM-AP**	0.03	0.03	-0.02	0.08	1	6431	14432
**AM-AM vs. AP-AP**	0.03	0.05	-0.06	0.13	1	6048	12994
**AM-AM vs. AP-AT**	0.05	0.05	-0.05	0.14	1	6236	13557
**AM-AM vs. AT-AT**	-0.2	0.07	-0.34	-0.06	1	7763	16665
**A-A vs. A-J (age)**	0.02	0.02	-0.03	0.06	1	6540	15047

**A-A vs. J-J (age)**	0.04	0.04	-0.04	0.13	1	6148	13441
**F-F vs F-M (sex)**	0.01	0.02	-0.03	0.06	1	6925	15745
**F-F vs. M-M (sex)**	0.03	0.05	-0.06	0.12	1	6415	14226
**Nest (same nest)**	-0.03	0.03	-0.08	0.02	1	126055	75122
**Year (same year)**	0	0.01	-0.01	0.02	1	103749	81249
**Hypothesis testing: remaining comaprisons**	**Estimate**	**Est.Error**	**l-95% CI**	**u-95% CI**			
**AP-AP vs. AP-AT**	-0.02	0.03	-0.08	0.05			
**AP-AP vs. AP-AM**	0	0.02	-0.05	0.05			
**AT-AT vs. AT-AP**	-0.25	0.04	-0.33	-0.17			
**AT-AT vs. AT-AM**	-0.2	0.04	-0.28	-0.12			
**AT-AT vs. AP-AP**	-0.2	0.04	-0.28	-0.12			
J-J vs. AJ (age)	0.02	0.02	-0.02	0.07			
M-M vs. F-M (sex)	0.01	0.02	-0.03	0.06			

AM (*Anarhynchus marginatus*); **AT** (*Anarhynchus thoracicus*); **AP** (*Anarhynchus pecuarius*); **F** (female); **M** (male)

AM-AM vs. AM-AT: W. UniFrac distances within *A. marginatus* (AM-AM) compared to between pairs of individuals from *A. marginatus* and *A. thoracicus* (AM-AT) (testing within vs. between species pairwise differences)
AM-AM vs. AM-AP: W. UniFrac distances within *A. marginatus* (AM-AM) compared to between pairs of individuals from *A. marginatus* and *A. pecuarius* (AM-AP) (testing within vs. between species pairwise differences)
AP-AP vs. AP-AT: W. UniFrac distances within *A. pecuarius* (AP-AP) compared to between pairs of individuals from *A. pecuarius* and *A. thoraciucs* (AP-AT) (testing within vs. between species pairwise differences)
AP-AP vs. AP-AM: W. UniFrac distances within *A. pecuarius* (AP-AP) compared to between pairs of individuals from *A. pecuarius* and *A. marginatus* (AP-AM) (testing within vs. between species pairwise differences)
AT-AT vs. AT-AP: W. UniFrac distances within *A. thoracicus* (AT-AT) compared to between pairs of individuals from *A. thoracicus* and *A. pecuarius* (AT-AP) (testing within vs. between species pairwise differences)
AT-AT vs. AT-AM: W. UniFrac distances within *A. thoracicus* (AT-AT) compared to between pairs of individuals from *A. thoracicus* and *A. marginatus* (AT-AM) (testing within vs. between species pairwise differences)
AM-AM vs. AP-AP: W. UniFrac distances within *A. marginatus* (AM-AM) compared to within *A. pecuarius* (AP-AP) (testing within species pairwise differences)
AM-AM vs. AT-AT: W. UniFrac distances within *A. marginatus* (AM-AM) compared to within *A. thoracicus* (AT-AT) (testing within species pairwise differences)
AT-AT vs. AP-AP: W. UniFrac distances within *A. thoracicus* (AT-AT) compared to within *A. pecuarius* (AP-AP) (testing within species pairwise differences)
F-F vs F-M (sex): W. UniFrac distances within females compared dissimilarities between sexes
F-F vs. M-M (sex): W. UniFrac distances within females compared to distances within males
A-A vs A-J (age): W. UniFrac distances within adults compared to distances between the two age groups
A-A vs. J-J (sex): W. UniFrac distances within adults compared to distances within juveniles
Nest-sharing: pairwise comparisons between individuals of same/different nests
Habitat-similarity: pairwise comparisons between individuals of same/different habitat

Table S19. Bayesian model results for Bray-Curtis dissimilarities - Adults

**Group-Level Effects:**							
**mmIDAIDB (Number of levels 65)**							
	**Estimate**	**Est.Error**	**l-95% CI**	**u-95% CI**	**Rhat**	**Bulk_ESS**	**Tail_ESS**
**sd(Intercept)**	0.79	0.08	0.65	0.95	1	12168	25963
**Population-Level Effects**							
	**Estimate**	**Est.Error**	**l-95% CI**	**u-95% CI**	**Rhat**	**Bulk_ESS**	**Tail_ESS**
**Intercept**	3.81	0.19	3.43	4.18	1	9107	16882
**AM-AM vs. AM-AT**	-0.21	0.15	-0.51	0.09	1	9465	19605
**AM-AM vs. AM-AP**	0	0.12	-0.24	0.23	1	8119	17795
**AM-AM vs. AP-AP**	-0.18	0.23	-0.63	0.27	1	7759	15888
**AM-AM vs. AP-AT**	-0.28	0.22	-0.71	0.16	1	7864	16291
**AM-AM vs. AT-AT**	-0.47	0.3	-1.07	0.13	1	9287	19256
**F-F vs F-M (sex)**	0.1	0.11	-0.12	0.32	1	9664	19758
**F-F vs. M-M (sex)**	0.17	0.22	-0.25	0.6	1	9116	17498
**Nest-sharing:**	-0.27	0.14	-0.52	0.01	1	92670	67649
**Year-similarity**	0	0.03	-0.05	0.06	1	89250	76748
**Hypothesis testing: remaining comparisons**	**Estimate**	**Est.Error**	**l-95% CI**	**u-95% CI**			
**AP-AP vs. AP-AT**	0.1	0.15	-0.2	0.4			
**AP-AP vs. AP-AM**	-0.17	0.12	-0.41	0.06			
**AT-AT vs. AT-AP**	-0.2	0.17	-0.52	0.13			
**AT-AT vs. AT-AM**	-0.26	0.17	-0.59	0.07			
**AT-AT vs. AP-AP**	-0.3	0.3	-0.89	0.3			
M-M vs. F-M (sex)	0.08	0.11	-0.14	0.29			

AM (*Anarhynchus marginatus*); **AT** (*Anarhynchus thoracicus*); **AP** (*Anarhynchus pecuarius*); **F** (female); **M** (male)

AM-AM vs. AM-AT: Dissimilarities within *A. marginatus* (AM-AM) compared to between pairs of individuals from *A. marginatus* and *A. thoracicus* (AM-AT) (testing within vs. between species pairwise differences)
AM-AM vs. AM-AP: Dissimilarities within *A. marginatus* (AM-AM) compared to between pairs of individuals from *A. marginatus* and *A. pecuarius* (AM-AP) (testing within vs. between species pairwise differences)
AP-AP vs. AP-AT: Dissimilarities within *A. pecuarius* (AP-AP) compared to between pairs of individuals from *A. pecuarius* and *A. thoraciucs* (AP-AT) (testing within vs. between species pairwise differences)
AP-AP vs. AP-AM: Dissimilarities within *A. pecuarius* (AP-AP) compared to between pairs of individuals from *A. pecuarius* and *A. marginatus* (AP-AM) (testing within vs. between species pairwise differences)
AT-AT vs. AT-AP: Dissimilarities within *A. thoracicus* (AT-AT) compared to between pairs of individuals from *A. thoracicus* and *A. pecuarius* (AT-AP) (testing within vs. between species pairwise differences)
AT-AT vs. AT-AM: Dissimilarities within *A. thoracicus* (AT-AT) compared to between pairs of individuals from *A. thoracicus* and *A. marginatus* (AT-AM) (testing within vs. between species pairwise differences)
AM-AM vs. AP-AP: Dissimilarities within *A. marginatus* (AM-AM) compared to within *A. pecuarius* (AP-AP) (testing within species pairwise differences)
AM-AM vs. AT-AT: Dissimilarities within *A. marginatus* (AM-AM) compared to within *A. thoracicus* (AT-AT) (testing within species pairwise differences)
AT-AT vs. AP-AP: Dissimilarities within *A. thoracicus* (AT-AT) compared to within *A. pecuarius* (AP-AP) (testing within species pairwise differences)

F-F vs F-M (sex): Dissimilarity within females compared dissimilarities between sexes
F-F vs. M-M (sex): Dissimilarity within females compared to dissimilarities within males
Nest-sharing: pairwise comparisons between individuals of same/different nests
Habitat-similarity: pairwise comparisons between individuals of same/different habitat

Table S20. Bayesian model results for Weighted UniFrac distances - Adults

**Group-Level Effects:**							
**mmIDAIDB (Number of levels 65)**							
	**Estimate**	**Est.Error**	**l-95% CI**	**u-95% CI**	**Rhat**	**Bulk_ESS**	**Tail_ESS**
**sd(Intercept)**	0.26	0.03	0.21	0.31	1	19262	29629
**Population-Level Effects**							
	**Estimate**	**Est.Error**	**l-95% CI**	**u-95% CI**	**Rhat**	**Bulk_ESS**	**Tail_ESS**
**Intercept**	-3.22	0.06	-3.34	-3.09	1	16100	29541
**AM-AM vs. AM-AT**	-0.11	0.05	-0.21	0	1	17995	34755
**AM-AM vs. AM-AP**	0.03	0.04	-0.05	0.11	1	14985	30504
**AM-AM vs. AP-AP**	0.04	0.08	-0.11	0.19	1	13756	26529
**AM-AM vs. AP-AT**	-0.07	0.07	-0.22	0.07	1	14244	27361
**AM-AM vs. AT-AT**	-0.33	0.11	-0.54	-0.11	1	18451	35741
**F-F vs F-M (sex)**	0.04	0.04	-0.04	0.12	1	17739	33346
**F-F vs. M-M (sex)**	0.08	0.07	-0.06	0.23	1	15643	28947
**Nest (same nest)**	-0.16	0.08	-0.32	-0.01	1	138942	69779
**Year (same year)**	0.02	0.01	-0.01	0.04	1	121391	77677
**Hypothesis testing: remaining comparisons**	**Estimate**	**Est.Error**	**l-95% CI**	**u-95% CI**			
**AP-AP vs. AP-AT**	0.11	0.05	0.01	0.21			
**AP-AP vs. AP-AM**	0.01	0.04	-0.07	0.09			
**AT-AT vs. AT-AP**	-0.25	0.07	-0.39	-0.12			
**AT-AT vs. AT-AM**	-0.22	0.07	-0.36	-0.08			
**AT-AT vs. AP-AP**	-0.37	0.11	-0.58	-0.15			
M-M vs. F-M (sex)	0.04	0.04	-0.03	0.12			

AM (*Anarhynchus marginatus*); **AT** (*Anarhynchus thoracicus*); **AP** (*Anarhynchus pecuarius*); **F** (female); **M** (male)

AM-AM vs. AM-AT: W. UniFrac distances within *A. marginatus* (AM-AM) compared to between pairs of individuals from *A. marginatus* and *A. thoracicus* (AM-AT) (testing within vs. between species pairwise differences)
AM-AM vs. AM-AP: W. UniFrac distances within *A. marginatus* (AM-AM) compared to between pairs of individuals from *A. marginatus* and *A. pecuarius* (AM-AP) (testing within vs. between species pairwise differences)
AP-AP vs. AP-AT: W. UniFrac distances within *A. pecuarius* (AP-AP) compared to between pairs of individuals from *A. pecuarius* and *A. thoraciucs* (AP-AT) (testing within vs. between species pairwise differences)
AP-AP vs. AP-AM: W. UniFrac distances within *A. pecuarius* (AP-AP) compared to between pairs of individuals from *A. pecuarius* and *A. marginatus* (AP-AM) (testing within vs. between species pairwise differences)

AT-AT vs. AT-AP: W. UniFrac distances within *A. thoracicus* (AT-AT) compared to between pairs of individuals from *A. thoracicus* and *A. pecuarius* (AT-AP) (testing within vs. between species pairwise differences)
AT-AT vs. AT-AM: W. UniFrac distances within *A. thoracicus* (AT-AT) compared to between pairs of individuals from *A. thoracicus* and *A. marginatus* (AT-AM) (testing within vs. between species pairwise differences)
AM-AM vs. AP-AP: W. UniFrac distances within *A. marginatus* (AM-AM) compared to within *A. pecuarius* (AP-AP) (testing within species pairwise differences)
AM-AM vs. AT-AT: W. UniFrac distances within *A. marginatus* (AM-AM) compared to within *A. thoracicus* (AT-AT) (testing within species pairwise differences)
AT-AT vs. AP-AP: W. UniFrac distances within *A. thoracicus* (AT-AT) compared to within *A. pecuarius* (AP-AP) (testing within species pairwise differences)
F-F vs F-M (sex): W. UniFrac distances within females compared dissimilarities between sexes
F-F vs. M-M (sex): W. UniFrac distances within females compared to distanCes within males
Nest-sharing: pairwise comparisons between individuals of same/different nests
Habitat-similarity: pairwise comparisons between individuals of same/different habitat

Table S21. Bayesian model results for Bray-Curtis dissimilarities - Juveniles

**Group-Level Effects:**							
**mmIDAIDB (Number of levels 65)**							
	**Estimate**	**Est.Error**	**l-95% CI**	**u-95% CI**	**Rhat**	**Bulk_ESS**	**Tail_ESS**
**sd(Intercept)**	0.58	0.06	0.48	0.7	1	16925	27788
**Population-Level Effects**							
	**Estimate**	**Est.Error**	**l-95% CI**	**u-95% CI**	**Rhat**	**Bulk_ESS**	**Tail_ESS**
**Intercept**	3.85	0.14	3.58	4.12	1	12657	23127
**AM-AM vs. AM-AT**	-0.07	0.12	-0.3	0.16	1	15219	30332
**AM-AM vs. AM-AP**	0.05	0.08	-0.11	0.21	1	12867	26033
**AM-AM vs. AP-AP**	-0.14	0.15	-0.44	0.16	1	11750	22000
**AM-AM vs. AP-AT**	0.09	0.16	-0.22	0.4	1	12723	25325
**AM-AM vs. AT-AT**	-0.52	0.23	-0.98	-0.06	1	14926	29408
**F-F vs F-M (sex)**	0.02	0.08	-0.14	0.17	1	12916	25905
**F-F vs. M-M (sex)**	0.08	0.14	-0.21	0.36	1	11663	22120
**Nest (same nest)**	-0.36	0.11	-0.56	-0.14	1	118520	73174
**Year (same year)**	-0.06	0.03	-0.11	0	1	102711	73782
**Hypothesis testing: remaining comaprisons**	**Estimate**	**Est.Error**	**l-95% CI**	**u-95% CI**			
**AP-AP vs. AP-AT**	-0.23	0.12	-0.46	0			
**AP-AP vs. AP-AM**	-0.19	0.08	-0.35	-0.03			
**AT-AT vs. AT-AP**	-0.61	0.14	-0.88	-0.34			
**AT-AT vs. AT-AM**	-0.45	0.14	-0.72	-0.18			

**AT-AT vs. AP-AP**	-0.38	0.23	-0.84	0.08			
M-M vs. F-M (sex)	0.06	0.07	-0.09	0.21			

AM (*Anarhynchus marginatus*); **AT** (*Anarhynchus thoracicus*); **AP** (*Anarhynchus pecuarius*); **F** (female); **M** (male)

AM-AM vs. AM-AT: Dissimilarities within *A. marginatus* (AM-AM) compared to between pairs of individuals from *A. marginatus* and *A. thoracicus* (AM-AT) (testing within vs. between species pairwise differences)
AM-AM vs. AM-AP: Dissimilarities within *A. marginatus* (AM-AM) compared to between pairs of individuals from *A. marginatus* and *A. pecuarius* (AM-AP) (testing within vs. between species pairwise differences)
AP-AP vs. AP-AT: Dissimilarities within *A. pecuarius* (AP-AP) compared to between pairs of individuals from *A. pecuarius* and *A. thoraciucs* (AP-AT) (testing within vs. between species pairwise differences)
AP-AP vs. AP-AM: Dissimilarities within *A. pecuarius* (AP-AP) compared to between pairs of individuals from *A. pecuarius* and *A. marginatus* (AP-AM) (testing within vs. between species pairwise differences)
AT-AT vs. AT-AP: Dissimilarities within *A. thoracicus* (AT-AT) compared to between pairs of individuals from *A. thoracicus* and *A. pecuarius* (AT-AP) (testing within vs. between species pairwise differences)
AT-AT vs. AT-AM: Dissimilarities within *A. thoracicus* (AT-AT) compared to between pairs of individuals from *A. thoracicus* and *A. marginatus* (AT-AM) (testing within vs. between species pairwise differences)
AM-AM vs. AP-AP: Dissimilarities within *A. marginatus* (AM-AM) compared to within *A. pecuarius* (AP-AP) (testing within species pairwise differences)
AM-AM vs. AT-AT: Dissimilarities within *A. marginatus* (AM-AM) compared to within *A. thoracicus* (AT-AT) (testing within species pairwise differences)
AT-AT vs. AP-AP: Dissimilarities within *A. thoracicus* (AT-AT) compared to within *A. pecuarius* (AP-AP) (testing within species pairwise differences)
F-F vs F-M (sex): Dissimilarity within females compared dissimilarities between sexes
F-F vs. M-M (sex): Dissimilarity within females compared to dissimilarities within males
Nest-sharing: pairwise comparisons between individuals of same/different nests
Habitat-similarity: pairwise comparisons between individuals of same/different habitat

Table S22. Bayesian model results for Weighted UniFrac distances - Juveniles

**Group-Level Effects:**							
**mmIDAIDB (Number of levels 65)**							
	**Estimate**	**Est.Error**	**l-95% CI**	**u-95% CI**	**Rhat**	**Bulk_ESS**	**Tail_ESS**
**sd(Intercept)**	0.24	0.02	0.2	0.29	1	17303	26969
**Population-Level Effects**							
	**Estimate**	**Est.Error**	**l-95% CI**	**u-95% CI**	**Rhat**	**Bulk_ESS**	**Tail_ESS**
**Intercept**	-3.14	0.06	-3.26	-3.03	1	12699	24230
**AM-AM vs. AM-AT**	0.09	0.05	-0.01	0.19	1	16002	28635
**AM-AM vs. AM-AP**	0.01	0.03	-0.06	0.08	1	12692	26109
**AM-AM vs. AP-AP**	-0.01	0.06	-0.14	0.11	1	11432	21829
**AM-AM vs. AP-AT**	0.17	0.07	0.04	0.3	1	12161	23809
**AM-AM vs. AT-AT**	-0.18	0.11	-0.4	0.03	1	17179	32857
**F-F vs F-M (sex)**	0	0.03	-0.07	0.07	1	14509	27307

**F-F vs. M-M (sex)**	-0.02	0.06	-0.14	0.1	1	12694	23426
**Nest (same nest)**	0.05	0.07	-0.09	0.17	1	94018	69910
**Year (same year)**	0	0.01	-0.03	0.03	1	74578	71076
**Hypothesis testing: remaining comparisons**	**Estimate**	**Est.Error**	**l-95% CI**	**u-95% CI**			
**AP-AP vs. AP-AT**	-0.18	0.05	-0.28	-0.08			
**AP-AP vs. AP-AM**	-0.02	0.03	-0.09	0.05			
**AT-AT vs. AT-AP**	-0.35	0.07	-0.5	-0.21			
**AT-AT vs. AT-AM**	-0.28	0.07	-0.42	-0.13			
**AT-AT vs. AP-AP**	-0.17	0.11	-0.38	0.04			
M-M vs. F-M (sex)	-0.02	0.03	-0.08	0.05			

AM (*Anarhynchus marginatus*); **AT** (*Anarhynchus thoracicus*); **AP** (*Anarhynchus pecuarius*); **F** (female); **M** (male)

AM-AM vs. AM-AT: W. UniFrac distances within *A. marginatus* (AM-AM) compared to between pairs of individuals from *A. marginatus* and *A. thoracicus* (AM-AT) (testing within vs. between species pairwise differences)
AM-AM vs. AM-AP: W. UniFrac distances within *A. marginatus* (AM-AM) compared to between pairs of individuals from *A. marginatus* and *A. pecuarius* (AM-AP) (testing within vs. between species pairwise differences)
AP-AP vs. AP-AT: W. UniFrac distances within *A. pecuarius* (AP-AP) compared to between pairs of individuals from *A. pecuarius* and *A. thoraciucs* (AP-AT) (testing within vs. between species pairwise differences)
AP-AP vs. AP-AM: W. UniFrac distances within *A. pecuarius* (AP-AP) compared to between pairs of individuals from *A. pecuarius* and *A. marginatus* (AP-AM) (testing within vs. between species pairwise differences)
AT-AT vs. AT-AP: W. UniFrac distances within *A. thoracicus* (AT-AT) compared to between pairs of individuals from *A. thoracicus* and *A. pecuarius* (AT-AP) (testing within vs. between species pairwise differences)
AT-AT vs. AT-AM: W. UniFrac distances within *A. thoracicus* (AT-AT) compared to between pairs of individuals from *A. thoracicus* and *A. marginatus* (AT-AM) (testing within vs. between species pairwise differences)
AM-AM vs. AP-AP: W. UniFrac distances within *A. marginatus* (AM-AM) compared to within *A. pecuarius* (AP-AP) (testing within species pairwise differences)
AM-AM vs. AT-AT: W. UniFrac distances within *A. marginatus* (AM-AM) compared to within *A. thoracicus* (AT-AT) (testing within species pairwise differences)
AT-AT vs. AP-AP: W. UniFrac distances within *A. thoracicus* (AT-AT) compared to within *A. pecuarius* (AP-AP) (testing within species pairwise differences)
F-F vs F-M (sex): W. UniFrac distances within females compared dissimilarities between sexes
F-F vs. M-M (sex): W. UniFrac distances within females compared to distances within males
Nest-sharing: pairwise comparisons between individuals of same/different nests
Habitat-similarity: pairwise comparisons between individuals of same/different habitat
