# Supplementary material for: The gut microbiota of three avian species living in sympatry
Source: BMC Ecol Evol. 2024 Nov 21;24:144. doi: 10.1186/s12862-024-02329-9 (PMC11580620; doi:10.1186/s12862-024-02329-9)
Supplement: Supplementary file 2 — Additional file 2. Appendix B. Quality control workflow and results. [file 12862_2024_2329_MOESM2_ESM.pdf]

Quality control - community standards workflow

Table of Contents

Quality control - community standards workflow

- Table of Contents
- 1. Activate Qiime2
- 2. Import expected taxonomic composition of mock samples
- 2. Get the observed taxonomic composition of mock samples
  - 2.1 Import results after filtering steps
  - 2.2 Inspect the taxonomic composition of the community standards (taxa bar plots only for positive controls)
- 3. Agglomerate taxa at species level
  - 3.1 Convert sequence counts into relative abundances
- 4. Compare observed and expected taxonomic composition of mock samples
- 5. Feature evaluation results
  - 5.1 Per-level accuracy
  - 5.2 Linear Regression between observed and expected abundances
  - 5.2 False positives: misclassifications
  - 5.3 False positives: underclassifications
  - 5.4 False negatives

1. Activate Qiime2

```
# Activate base env
. ~/.bashrc

# Activate qiime2
conda activate qiime2-amplicon-2023.
```

2. Import expected taxonomic composition of mock samples

Composition of comunity standard available here: https://www.zymoresearch.de/collections/zymobiomics-microbial-community-standards/products/zymobiomics-microbial-community-standard

For the purpose of this analysis, eukaryote taxa were excluded from the community standards' composition.

```
biom convert -i mock_expected.tsv -o mock-expected.biom --table-type="OTU table" --to-hdf5

qiime tools import --input-path mock-expected.biom --type 'FeatureTable[RelativeFrequency]' --
input-format BIOMV210Format --output-path mock-expected.qza
```

2. Get the observed taxonomic composition of mock samples

2.1 Import results after filtering steps

```
qiime feature-table filter-samples --i-table filtered-table.qza --m-metadata-file
plover_metadata.tsv --p-where "sample_type='positive'" --p-no-exclude-ids --o-filtered-table
mock-observed.qza

qiime feature-table summarize --i-table mock-observed.qza --o-visualization mock-observed.qzv -
-m-sample-metadata-file plover_metadata.tsv
```

2.2 Inspect the taxonomic composition of the community standards (taxa bar plots only for positive controls)

```
qiime taxa barplot --i-table mock-observed.qza --i-taxonomy taxonomy.qza --m-metadata-file
plover_metadata.tsv --o-visualization mock-observed-bar-plot.qzv
```

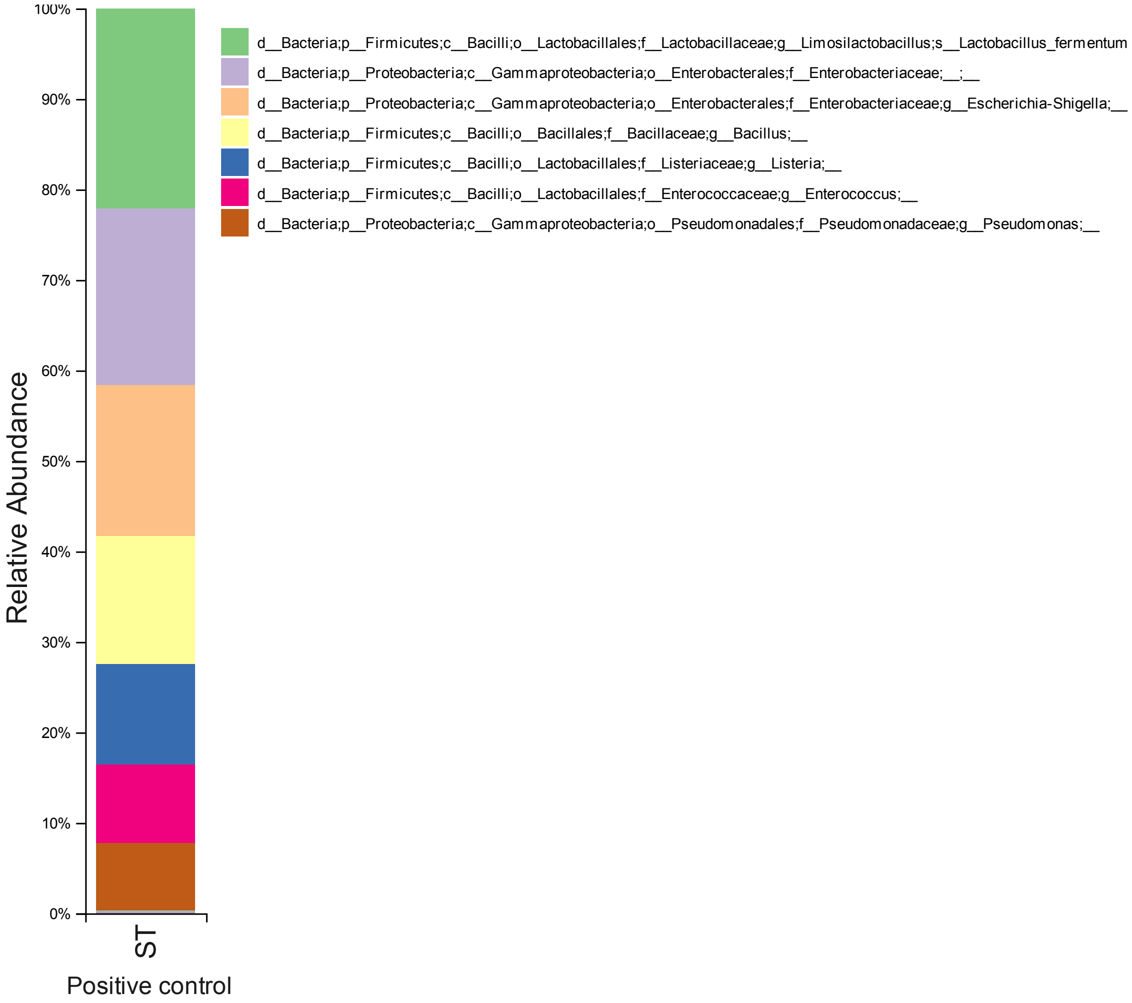

3. Agglomerate taxa at species level

```
qiime taxa collapse --i-table mock-observed.qza --i-taxonomy taxonomy.qza --p-level 7 --o-
collapsed-table mock-observed-l7.qza
```

3.1 Convert sequence counts into relative abundances

```
qiime feature-table relative-frequency --i-table mock-observed-l7.qza --o-relative-frequency-table mock-observed-l7-rel.qza
```

4. Compare observed and expected taxonomic composition of mock samples

```
qiime quality-control evaluate-composition --i-expected-features mock-expected.qza --i-observed-features mock-observed-l7-rel.qza --o-visualization mock-comparison.qzv
```

5. Feature evaluation results

Taxon accuracy rate (**TAR**) and taxon detection rate (**TDR**) are used for qualitative compositional analyses of mock communities.

At a given taxonomic level, a classification is a:

True positive (TP), if that taxon is both observed and expected.

False positive (FP), if that taxon is observed but not expected.

False negative (FN), if a taxon is expected but not observed.

These are used to calculate TAR and TDR as:

TAR = TP/(TP + FP) Fraction of observed taxa that were expected at level L.

TDR = TP/(TP + FN) Fraction of expected taxa that are observed at level L.

5.1 Per-level accuracy

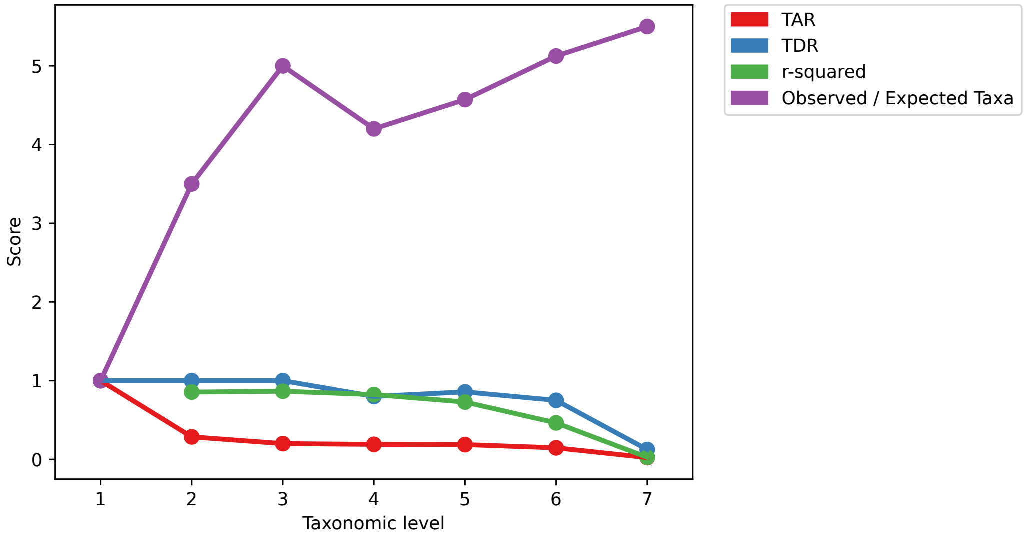

	sample	level	Observed Taxa	Observed / Expected Taxa	TAR	TDR	Slope	Intercept	r-value	P value	Std Err	Bray-Curtis	Jaccard	r-squared
0	ST	1	1	1	1	1						5.55E-17	0	
1	ST	2	7	3.5	0.285714286	1	0.800729693	0.028467187	0.92475196	0.00286389	0.147370532	0.190373726	0.714285714	0.855166187
2	ST	3	10	5	0.2	1	0.81475674	0.018524326	0.930498875	9.38E-05	0.113396019	0.191820455	0.8	0.865828157
3	ST	4	21	4.2	0.19047619	0.8	1.008687218	-0.000394874	0.907348208	5.71E-09	0.104498352	0.191890412	0.818181818	0.82328077

	sample	level	Observed Taxa	Observed / Expected Taxa	TAR	TDR	Slope	Intercept	r-value	P value	Std Err	Bray-Curtis	Jaccard	r-squared
4	ST	5	32	4.571428571	0.1875	0.857142857	1.040264799	-0.001220145	0.853466821	2.77E-10	0.11408717	0.229514106	0.818181818	0.728405615
5	ST	6	41	5.125	0.146341463	0.75	0.735334808	0.006155004	0.680917402	5.04E-07	0.123516083	0.333514106	0.860465116	0.463648508
6	ST	7	44	5.5	0.022727273	0.125	0.174548718	0.016185319	0.161979864	0.256120341	0.151909216	0.816	0.980392157	0.026237476

5.2 Linear Regression between observed and expected abundances

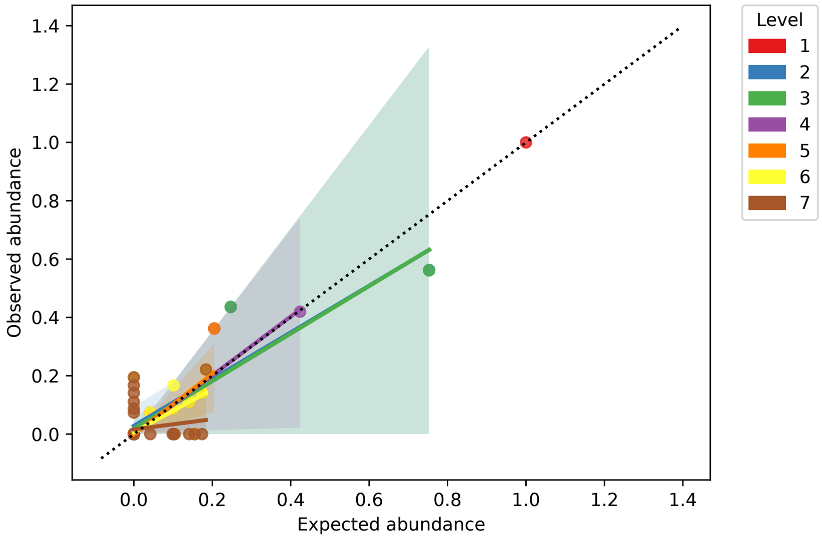

5.2 False positives: misclassifications

Misclassifications were then removed from all samples. All misclassified taxa detected at really low abundances

Taxon	ST
dBacteria;pBacteroidota;cBacteroidia;oBacteroidales;fBacteroidaceae;gBacteroides;__	0.00021547
dBacteria;pBacteroidota;cBacteroidia;oBacteroidales;fBacteroidaceae;gBacteroides;s__Bacteroides_acidifaciens	2.24E-05
dBacteria;pBacteroidota;cBacteroidia;oBacteroidales;fMuribaculaceae;gMuribaculaceae;s__uncultured_Bacteroidales	8.12E-05
dBacteria;pBacteroidota;cBacteroidia;oBacteroidales;fMuribaculaceae;gMuribaculaceae;s__uncultured_bacterium	5.88E-05
dBacteria;pBacteroidota;cBacteroidia;oBacteroidales;fMuribaculaceae;gMuribaculaceae;s__uncultured_organism	2.24E-05
dBacteria;pBacteroidota;cBacteroidia;oBacteroidales;fPrevotellaceae;gPrevotellaceae_Ga6A1_group;s__uncultured_bacterium	5.32E-05
dBacteria;pBacteroidota;cBacteroidia;oBacteroidales;fRikenellaceae;gRikenella;s__uncultured_bacterium	9.23E-05
dBacteria;pBacteroidota;cBacteroidia;oCytophagales;fHymenobacteraceae;gPontibacter;__	5.60E-06
dBacteria;pCyanobacteria;cCyanobacteriia;oCyanobacteriales;fColeofasciculaceae;gMicrocoleus_SAG_1449-1a;s__uncultured_cyanobacterium	1.68E-05
dBacteria;pCyanobacteria;cCyanobacteriia;oCyanobacteriales;fPhormidiaceae;gArthrospira_PCC-7345;s__uncultured_cyanobacterium	3.92E-05
dBacteria;pDeferribacterota;cDeferribacteres;oDeferribacterales;fDeferribacteraceae;gMucispirillum;s__bacterium_Lincoln	5.88E-05
dBacteria;pFirmicutes;cBacilli;oErysipelotrichales;fErysipelotrichaceae;;__	3.64E-05
dBacteria;pFirmicutes;cBacilli;oErysipelotrichales;fErysipelotrichaceae;gZOR0006;s__uncultured_bacterium	3.36E-05
dBacteria;pFirmicutes;cBacilli;oLactobacillales;fCatellicoccaceae;gCatellicoccus;s__uncultured_bacterium	0.000218269
dBacteria;pFirmicutes;cBacilli;oLactobacillales;fLactobacillaceae;gLactobacillus;s__Lactobacillus_iners	3.08E-05
dBacteria;pFirmicutes;cBacilli;oLactobacillales;fStreptococcaceae;gLactococcus;s__Lactococcus_lactis	1.40E-05
dBacteria;pFirmicutes;cClostridia;oLachnospirales;fLachnospiraceae;;__	0.000165101
dBacteria;pFirmicutes;cClostridia;oLachnospirales;fLachnospiraceae;gAgathobacter;__	0.000125924
dBacteria;pFirmicutes;cClostridia;oLachnospirales;fLachnospiraceae;gTuzzerella;s__uncultured_bacterium	0.000100739
dBacteria;pFirmicutes;cClostridia;oLachnospirales;fLachnospiraceae;gTyzzerella;s_*[Clostridium]*colinum	0.000167899
dBacteria;pFirmicutes;cClostridia;oOscillospirales;fButyricicoccaceae;gButyricicoccus;__	0.000145512
dBacteria;pFirmicutes;cClostridia;oOscillospirales;fOscillospiraceae;gColidextribacter;__	0.000111933
dBacteria;pFirmicutes;cClostridia;oOscillospirales;fRuminococcaceae;gFaecalibacterium;__	0.000237857

Taxon	ST
dBacteria;pFirmicutes;cClostridia;oOscillospirales;fRuminococcaceae;gPaludicola;s__uncultured_bacterium	7.00E-05
dBacteria;pFirmicutes;cClostridia;oOscillospirales;fRuminococcaceae;gSubdoligranulum;__	4.48E-05
dBacteria;pFirmicutes;cClostridia;oPeptostreptococcales-Tissierellales;fPeptostreptococcaceae;gPeptoclostridium;__	7.84E-05
dBacteria;pFirmicutes;cNegativicutes;oAcidaminococcales;fAcidaminococcaceae;gPhascolarctobacterium;__	1.68E-05
dBacteria;pFirmicutes;cNegativicutes;oVeillonellales-Selenomonadales;fSelenomonadaceae;gMegamonas;s__Megamonas_funiformis	0.000167899
dBacteria;pFirmicutes;cNegativicutes;oVeillonellales-Selenomonadales;fVeillonellaceae;gMegasphaera;s__Megasphaera_elsdenii	1.40E-05
dBacteria;pFusobacteriota;cFusobacteriia;oFusobacteriales;fFusobacteriaceae;gFusobacterium;s__Fusobacterium_mortiferum	0.000509293
dBacteria;pProteobacteria;cAlphaproteobacteria;oRhizobiales;fXanthobacteraceae;;__	5.32E-05
dBacteria;pProteobacteria;cAlphaproteobacteria;oRhodobacterales;fRhodobacteraceae;gParacoccus;__	0.000125924
dBacteria;pProteobacteria;cGammaproteobacteria;oBurkholderiales;fAlcaligenaceae;gAchromobacter;__	2.52E-05
dBacteria;pProteobacteria;cGammaproteobacteria;oCardiobacteriales;fWohlfahrtiimonadaceae;gKoukoulia;s__Koukoulia_aurantiaca	1.40E-05
dBacteria;pProteobacteria;cGammaproteobacteria;oEnterobacterales;fEnterobacteriaceae;gCitrobacter;__	0.000310613
dBacteria;pProteobacteria;cGammaproteobacteria;oXanthomonadales;fRhodanobacteraceae;gChujaibacter;__	5.60E-06
dBacteria;pVerrucomicrobiota;cVerrucomicrobiae;oVerrucomicrobiales;fAkkermansiaceae;gAkkermansia;s__uncultured_bacterium	0.000237857

5.3 False positives: underclassifications

Taxon	ST
dBacteria;pFirmicutes;cBacilli;oBacillales;fBacillaceae;gBacillus;__	0.141354048
dBacteria;pFirmicutes;cBacilli;oLactobacillales;fEnterococcaceae;gEnterococcus;__	0.087273826
dBacteria;pFirmicutes;cBacilli;oLactobacillales;fListeriaceae;gListeria;__	0.110858019
dBacteria;pProteobacteria;cGammaproteobacteria;oEnterobacterales;fEnterobacteriaceae;;__	0.194743087
dBacteria;pProteobacteria;cGammaproteobacteria;oEnterobacterales;fEnterobacteriaceae;gEscherichia-Shigella;__	0.166983809
dBacteria;pProteobacteria;cGammaproteobacteria;oPseudomonadales;fPseudomonadaceae;gPseudomonas;__	0.073699204

5.4 False negatives

The taxonomy classifier can accurately assign taxonomy only up to the genus level. Since, all community standards are a priory identified at species level, this leads to the classification of every feature as a false negative.

Taxon	ST
dBacteria;pFirmicutes;cBacilli;oBacillales;fBacillaceae;gBacillus;s__Bacillus_subtilis	0.174
dBacteria;pFirmicutes;cBacilli;oLactobacillales;fEnterococcaceae;gEnterococcus;s__Enterococcus_faecalis	0.099
dBacteria;pFirmicutes;cBacilli;oLactobacillales;fListeriaceae;gListeria;s__Listeria_monocytogenes	0.141
dBacteria;pFirmicutes;cBacilli;oStaphylococcales;fStaphylococcaceae;gStaphylococcus;s__Staphylococcus_aureus	0.155
dBacteria;pProteobacteria;cGammaproteobacteria;oEnterobacterales;fEnterobacteriaceae;gEscherichia-Shigella;s__Escherichia_coli	0.101
dBacteria;pProteobacteria;cGammaproteobacteria;oEnterobacterales;fEnterobacteriaceae;gSalmonella;s__Salmonella_enterica	0.104
dBacteria;pProteobacteria;cGammaproteobacteria;oPseudomonadales;fPseudomonadaceae;gPseudomonas;s__Pseudomonas_aeruginosa	0.042
