# Supplementary material for: The gut microbiota of three avian species living in sympatry
Source: BMC Ecol Evol. 2024 Nov 21;24:144. doi: 10.1186/s12862-024-02329-9 (PMC11580620; doi:10.1186/s12862-024-02329-9)
Supplement: Supplementary file 3 — Additional file 3. Appendix C. Sequence data processing. [file 12862_2024_2329_MOESM3_ESM.pdf]

Sequence data processing

Table of Contents

Sequence data processing

- Table of Contents
- 1. Activate Qiime2
- 2. Import sequences
- 3. Visualize quality plots
- 4. Run dada2
  - 4.1. DADA2 results
- 5. Taxonomy assignment
  - 5.1. Taxonomy visualisation
- 6. Exit qiime2
- 7. In R run decontam
  - 7.1. Read in the data
  - 7.2. Run decontam
  - 7.3. Export feature table as biom file
- 8. Import biom table from R to qiime2
- 9. Remove negative controls from dataset
- 10. Taxonomy based filtering
- 11. Filter unique features
- 12. Filter samples with less than 500 reads
- 13. Filter based on abundance (0.001%) and prevalence (1% of samples)
- 14. Extract community standard (in order to perform quality control steps)
- 15. Remove false positives detected in the community standard
- 16. Import final table to qiime2
- 17. Filter representative sequences
- 18. Building a phylogenetic tree
- 19. Rarefaction curves
- 20. Taxa bar plots
  - 20.1. Plot core phylum (present in 95% of samples)
  - 20.2. Plot core families (present in 95% of samples)
- 21. Calculate alpha diversity metrics and rarefy the data-set
- 22. Filter samples with less than 27378 reads (unrarefied table for beta analysis)

---

1. Activate Qiime2

```
# Activate base env
. ~/.bashrc

# Activate qiime2
conda activate qiime2-amplicon-2023.9
```

2. Import sequences

```
qiime tools import --type 'SampleData[PairedEndSequencesWithQuality]'  --input-path seqs/ --
input-format CasavaOneEightLanelessPerSampleDirFmt --output-path demux-paired-end.qza
```

3. Visualize quality plots

```
qiime demux summarize --i-data demux-paired-end.qza --o-visualization demux-quality-plots.qzv
```

4. Run dada2

```
qiime dada2 denoise-paired --i-demultiplexed-seqs demux-paired-end.qza --p-trim-left-f 20 --p-
trim-left-r 20 --p-trunc-len-f 245 --p-trunc-len-r 242  --p-trunc-q 2 --o-table table.qza --o-
representative-sequences rep-seqs.qza --o-denoising-stats denoising-stats.qza
```

4.1. DADA2 results

```
qiime metadata tabulate --m-input-file denoising-stats.qza --o-visualization denoising-
stats.qzv
qiime feature-table tabulate-seqs --i-data rep-seqs.qza --o-visualization rep-seqs.qzv
qiime feature-table summarize --i-table table.qza --o-visualization table.qzv --m-sample-
metadata-file plover_metadata.tsv
```

5. Taxonomy assignment

```
qiime feature-classifier classify-sklearn --i-classifier silva-138.1-ssu-nr99-338F-806R-
classifier.qza --i-reads rep-seqs.qza --o-classification taxonomy.qza
```

5.1. Taxonomy visualisation

```
qiime metadata tabulate --m-input-file taxonomy.qza --o-visualization taxonomy.qzv
```

6. Exit qiime2

```
conda deactivate
```

7. In R run decontam

7.1. Read in the data

```
# Load Libraries
library(decontam)
library(qiime2R)
library(phyloseq)
library(biomformat)

# Make a phyloseq object
ps <- qza_to_phyloseq(features = "table.qza", taxonomy = "taxonomy.qza", metadata =
"buzzard_meta.tsv")

# Choose which samples are the negative controls
sample_data(ps)$is.neg <- sample_data(ps)$type == "negative"
```

7.2. Run decontam

```
# Identify contaminants based on prevalence method (treshold 0.1 is the standard)
contamdf.prev <- isContaminant(ps, method="prevalence", neg="is.neg", threshold=0.1)

table(contamdf.prev$contaminant)
head(which(contamdf.prev$contaminant))

# Remove contaminants from the phyloseq object
ps.nocontam <- prune_taxa(!contamdf.prev$contaminant,ps)
```

7.3. Export feature table as biom file

```
# Extract asv table from the phyloseq object
table_nocontam <- as(otu_table(ps.nocontam),"matrix",)

#'t' to transform if taxa_are_rows=FALSE table_nocontam<-
t(as(otu_table(ps.nocontam),"matrix",))#if taxa_are_rows=TRUE

# Make a biom table
table_nocontam_biom <- make_biom(data=table_nocontam)
write_biom(table_nocontam_biom,"table-nocontam.biom")
```

8. Import biom table from R to qiime2

```
conda activate qiime2-amplicon-2023.9

qiime tools import --input-path table-nocontam.biom --type 'FeatureTable[Frequency]' --input-
format BIOMV100Format --output-path table-nocontam.qza
```

9. Remove negative controls from dataset

```
qiime feature-table filter-samples --i-table table-nocontam.qza --m-metadata-file
buzzard_meta.tsv --p-where "type='negative'" --p-exclude-ids --o-filtered-table table-
nocontam.qza
```

10. Taxonomy based filtering

Filter out mitochondrial, chloroplast, unassigned, Vertebrata, Eukaryote and taxa not assigned to phylum

```
qiime taxa filter-table --i-table table-nocontam1.qza --i-taxonomy taxonomy.qza --p-exclude
mitochondria,chloroplast,Unassigned,Vertebrata,Eukaryota --p-include p_ --o-filtered-table
table-taxa-filter.qza

qiime feature-table summarize --i-table table-taxa-filter.qza --o-visualization table-taxa-
filter.qzv --m-sample-metadata-file plover_metadata.tsv
```

11. Filter unique features

```
qiime feature-table filter-features --i-table table-taxa-filter.qza --p-min-samples 2 --o-
filtered-table table-taxa-filter-no_singles.qza

qiime feature-table summarize --i-table table-taxa-filter-no_singles.qza --o-visualization
table-taxa-filter-no_singles.qzv --m-sample-metadata-file plover_metadata.tsv
```

12. Filter samples with less than 500 reads

```
qiime feature-table filter-samples --i-table table-taxa-filter-no_singles.qza --p-min-frequency
200 --o-filtered-table filtered-table.qza

qiime feature-table summarize --i-table filtered-table.qza  --o-visualization filtered-
table.qzv  --m-sample-metadata-file plover_metadata.tsv
```

13. Filter based on abundance (0.001%) and prevalence (1% of samples)

```
qiime feature-table filter-features-conditionally --i-table filtered-table.qza --p-abundance
0.0001 --p-prevalence 0.01 --o-filtered-table filtered-table.qza

qiime feature-table summarize --i-table filtered-table.qza --o-visualization filtered-table.qzv
 --m-sample-metadata-file plover_metadata.tsv
```

14. Extract community standard (in order to perform quality control steps)

```
qiime feature-table filter-samples --i-table filtered-table.qza --m-metadata-file
plover_metadata.tsv --p-where "sample_type='positive'" --p-no-exclude-ids --o-filtered-table
mock-observed.qza

qiime feature-table summarize --i-table mock-observed.qza --o-visualization mock-observed.qzv -
-m-sample-metadata-file plover_metadata.tsv
```

15. Remove false positives detected in the community standard

```
conda deactivate
conda activate R
R

library(qiime2R)
library(biomformat)
library(phyloseq)

setwdir <- "/grp/animalbehaviour/microbiome/plover-microbiome/"

ps <- qza_to_phyloseq(
  features="filtered-table.qza",
  taxonomy="taxonomy.qza",
  metadata = "plover_meta.tsv")


asv_to_remove <- c("9818f614002708e821a05e68ccfbb517", "f035c7827e0482a588b2650e9746999f",
"67fa2a1488f0af032b9276a8f80d05b4","43060bb7da9dd9dd8eb40ba4e445ed0e",
"c4d91c401d1c7906b9633e7436ff772d","083ae18ecc79279b466b007a62eb5224",
"d45c458113515f9da4778594caeae976", "f4f5d2c3489bb2be8cd937184fe22529",
"48931159c2f033e72ac9c20cf5bfca69", "4dbc8fb77c6fd2f38f808e6f330691a8",
"ea4e937d37ae4b78a72665978e1db94a", "deac3048ce5f1a2b70560a7858928fb0",
"db0b2a3fae9b8c108bce0d5392a61bc8", "d9e6dbb4d649656589ab8f3f5d385c84",
"f3e10dd6d8305b9f8e3e3cb438ce7773", "15d65d260f28470c2e311bd390be03bd",
"27d20a8aef120bea19246cfe1df61d0e", "0d988dd52b5c9d14de6a12a9f2f0f7d3",
"8fcdc6aa7cfd5aa5a6acebf77ca3c6d7", "b6d21346b741668306df08cda71ce804",
"f4976f71ce9d6d1528f1364bff92f824", "987f412e81957298ba3c8b17c134119a",
"c3c1655520785fe770affcabc22f9494", "03c83d862e8dfcbe16d1c1c068c3b217",
"a77f6092e226a1b69ae6f6a3e359418e", "764222cc215e748dff09fde3c3727e23",
"2e9f1c3013022ced2c0c9567d59fc064", "1bb4bc14144a82e73e78d1f4ea6a5c6a",
"5470cd4d4e30aa3deb956b490461b77b", "ffe7086915026fc6cf8f955029c6863a",
"876c7eabbfe5a0307a41ceca4119dc56", "bebd987cda4aea47992bc99e5074e987",
"9644303b7d8f582d594ed172d1bab168", "09f37541af41dd12b0a775db8fb92fae",
"f4860ce6ad68f0b4d013b85797d8b1a2", "248842cedda56f3febccea46c2b03499",
"39ecf3f32a5cb052bdb3ed64942037a6", "fb199352250651eefa45307ea2bc3bab",
"410da2592aa9ffc2d4ce769e9ae20f9c", "c2819eafded969efa72d2ef9b58446d4",
"4dfd2bf546d1388173eda78277ed8abc")

ps_filtered <- prune_taxa(!(taxa_names(ps) %in% asv_to_remove), ps)

asv_table <- otu_table(ps_filtered, taxa_are_rows = TRUE)

biom_table<- make_biom(asv_table)

write_biom(biom_table, "-filtered-table-final.biom")
```

16. Import final table to qiime2

```
conda deactivate
conda activate qiime2-amplicon-2023.9

qiime tools import --input-path filtered-table-final.biom --type 'FeatureTable[Frequency]' --
input-format BIOMV100Format --output-path filter-table-final.qza

qiime feature-table summarize --i-table filtered-table-final.qza --o-visualization filtered-
table-final.qzv --m-sample-metadata-file plover_metadata.tsv
```

17. Filter representative sequences

```
qiime feature-table filter-seqs --i-data rep-seqs.qza --i-table filtered-table-final.qza --o-
filtered-data filter-seqs.qza

qiime feature-table tabulate-seqs --i-data filter-seqs.qza --o-visualization filter-seqs.qzv
```

18. Building a phylogenetic tree

```
qiime phylogeny align-to-tree-mafft-fasttree --i-sequences filter-seqs.qza  --o-alignment
aligned-seqs.qza --o-masked-alignment masked-aligned-seqs.qza --o-tree unrooted-tree.qza  --o-
rooted-tree rooted-tree.qza
```

19. Rarefaction curves

```
qiime diversity alpha-rarefaction --i-table filtered-table-final.qza --i-phylogeny rooted-
tree.qza --p-max-depth 35323 --m-metadata-file plover_meta.tsv  --o-visualization alpha-
rarefaction-35323.qzv

qiime diversity alpha-rarefaction --i-table filtered-table-final.qza --i-phylogeny rooted-
tree.qza --p-max-depth 27378 --m-metadata-file plover_meta.tsv  --o-visualization alpha-
rarefaction-min.qzv

qiime diversity alpha-rarefaction --i-table filtered-table-final.qza --i-phylogeny rooted-
tree.qza --p-max-depth 185000 --m-metadata-file plover_meta.tsv  --o-visualization alpha-
rarefaction-mean.qzv
```

20. Taxa bar plots

```
qiime taxa barplot --i-table filtered-table-final.qza --i-taxonomy taxonomy.qza --m-metadata-
file plover_metadata.tsv --o-visualization taxa-bar-plots.qzv
```

20.1. Plot core phylum (present in 95% of samples)

```
qiime taxa collapse --i-table filtered-table-final.qza --i-taxonomy taxonomy.qza --o-collapsed-table phylum-table.qza  --p-level 2

qiime feature-table summarize --i-table phylum-table.qza --o-visualization phylum-table.qzv --m-sample-metadata-file plover_metadata.tsv

qiime feature-table core-features --i-table phylum-table.qza --o-visualization phylum-core-table.qzv
```

From the visualization file download the tsv relative to features present in 95% of the samples (or any other % that I want)

```
qiime metadata tabulate --m-input-file core-features-0.950.tsv --o-visualization core-features-0.950.qzv

qiime feature-table filter-features --i-table filtered-table.qza --o-filtered-table table-phylum-core-0.950.qza --m-metadata-file core-features-0.950.tsv

qiime taxa barplot --i-table table-phylum-core-0.950.qza --i-taxonomy taxonomy.qza --m-metadata-file buzzard_meta.tsv --o-visualization core-phylum-bar-plots.qzv
```

Feature ID	2%	9%	25%	50%	75%	91%	98%
dBacteria;pFirmicutes	11826	20893	38024	73685	188749	301047	430012
dBacteria;pProteobacteria	2712	5966	12203	21781	37156	68097	154347
dBacteria;pBacteroidota	1498	3911	7490	13795	32225	102504	204807
dBacteria;pActinobacteriota	137	992	2524	6103	11222	20180	35389
dBacteria;pFusobacteriota	277	713	1548	4434	38014	105732	203633
dBacteria;pDesulfobacterota	21	210	517	1133	3507	7372	16564
dBacteria;pCampylobacterota	0	58	204	503	869	3245	23081
dBacteria;pDeferribacterota	0	13	76	232	2117	6615	22141

20.2. Plot core families (present in 95% of samples)

```
qiime taxa collapse --i-table filtered-table-final.qza --i-taxonomy taxonomy.qza --o-collapsed-table family-table.qza  --p-level 5

qiime feature-table summarize --i-table -table.qza --o-visualization family-table.qzv --m-sample-metadata-file plover_metadata.tsv

qiime feature-table core-features --i-table family-table.qza --o-visualization family-core-table.qzv
```

From the visualization file download the tsv relative to features present in 95% of the samples (or any other % that I want)

```
qiime metadata tabulate --m-input-file core-features-0.950.tsv --o-visualization core-features-0.950.qzv

qiime feature-table filter-features --i-table filtered-table.qza --o-filtered-table table-family-core-0.950.qza --m-metadata-file core-features-0.950.tsv

qiime taxa barplot --i-table table-family-core-0.950.qza --i-taxonomy taxonomy.qza --m-metadata-file plover_metadata.tsv --o-visualization core-family-bar-plots.qzv
```

Feature ID	2%	9%	25%	50%	75%	91%	98%
dBacteria;pFirmicutes;cClostridia;oLachnospirales;f__Lachnospiraceae	1500	2456	4532	8925	25495	61735	136426
dBacteria;pFusobacteriota;cFusobacteriia;oFusobacteriales;f__Fusobacteriaceae	243	676	1502	4289	38014	105732	203633
dBacteria;pFirmicutes;cClostridia;oOscillospirales;f__Ruminococcaceae	388	843	1625	3162	13067	33729	82366
dBacteria;pBacteroidota;cBacteroidia;oBacteroidales;f__Bacteroidaceae	364	725	1604	3041	18737	75633	168487
dBacteria;pFirmicutes;cBacilli;oLactobacillales;f__Lactobacillaceae	90	212	998	3012	5885	10961	18873
dBacteria;pProteobacteria;cGammaproteobacteria;oEnterobacterales;f__Enterobacteriaceae	252	450	987	2388	5752	12901	67767
dBacteria;pFirmicutes;cClostridia;oPeptostreptococcales-Tissierellales;f__Peptostreptococcaceae	151	329	782	2031	9284	34501	66368
dBacteria;pBacteroidota;cBacteroidia;oBacteroidales;f__Prevotellaceae	55	250	730	1832	3378	11123	35825
dBacteria;pFirmicutes;cClostridia;oOscillospirales;f__Oscillospiraceae	326	483	785	1821	5898	22351	52490
dBacteria;pFirmicutes;cBacilli;oErysipelotrichales;f__Erysipelotrichaceae	106	282	541	1215	4780	15465	36425
dBacteria;pBacteroidota;cBacteroidia;oBacteroidales;f__Muribaculaceae	4	29	287	1167	2581	4593	10016
dBacteria;pFirmicutes;cClostridia;oClostridiales;f__Clostridiaceae	7	91	295	957	1985	6164	40916
dBacteria;pDesulfobacterota;cDesulfovibrionia;oDesulfovibrionales;f__Desulfovibrionaceae	21	147	369	810	1813	7327	16564
dBacteria;pBacteroidota;cBacteroidia;oBacteroidales;f__Rikenellaceae	12	130	284	747	1724	8681	17202
dBacteria;pProteobacteria;cGammaproteobacteria;oPseudomonadales;f__Moraxellaceae	0	21	134	699	1581	3762	36899
dBacteria;pFirmicutes;cClostridia;oOscillospirales;f__Butyricicoccaceae	0	73	176	547	4905	23486	67456
dBacteria;pBacteroidota;cBacteroidia;oBacteroidales;f__Tannerellaceae	0	50	215	491	1145	8762	21542
dBacteria;pFirmicutes;cBacilli;oLactobacillales;f__Streptococcaceae	0	29	124	473	1380	2222	3558
dBacteria;pDeferribacterota;cDeferribacteres;oDeferribacterales;f__Deferribacteraceae	0	13	76	232	2117	6615	22141
dBacteria;pFirmicutes;cNegativicutes;oVeillonellales-Selenomonadales;f__Selenomonadaceae	0	49	114	219	506	1080	1652

21. Calculate alpha diversity metrics and rarefy the data-set

```
# Calculates observed features, shannon diversity, Faith PD and retrieves rarefied table
qiime diversity core-metrics-phylogenetic --i-phylogeny rooted-tree.qza --i-table filtered-table-final.qza --p-sampling-depth 27378 --m-metadata-file plover_meta.tsv --output-dir alpha-metrics-results # rarefy to the sample with least reads

#Add alpha diversity metrics to the metadata
qiime metadata tabulate --m-input-file plover_metadata.tsv --m-input-file shannon_vector.qza --m-input-file observed_features_vector.qza --m-input-file faith_pd_vector.qza --o-visualization plover_meta_alpha.qzv
```

22. Filter samples with less than 27378 reads (unrarefied table for beta analysis)

```
qiime feature-table filter-samples --i-table filtered-table-final.qza  --p-min-frequency 27378
--o-filtered-table beta-table.qza

qiime feature-table summarize --i-table beta-table.qza --o-visualization beta-table.qzv --m-
sample-metadata-file buzzard_meta_alpha.tsv
```
