# Supplementary material for: The gut microbiota of three avian species living in sympatry
Source: BMC Ecol Evol. 2024 Nov 21;24:144. doi: 10.1186/s12862-024-02329-9 (PMC11580620; doi:10.1186/s12862-024-02329-9)
Supplement: Supplementary file 4 — Additional file 4. Appendix D. Beta diversity statistical analysis. [file 12862_2024_2329_MOESM4_ESM.pdf]

Beta Diversity Statistical Analysis

Beta Diversity Statistical Analysis
- A) PERMANOVA
  - 1. Read in the data
  - 2. CSS tranformation
  - 3. Perform PERMANOVA on Bray-Curtis dissimilarities and Weighted-UniFrac distances
  - 4. Perform PERMANOVA on Bray-Curtis dissimilarities and Weighted-UniFrac distances - Adult individuals only
  - 5. Perform PERMANOVA on Bray-Curtis dissimilarities and Weighted-UniFrac distances - Juveniles individuals only
- B) Model pairwise comparisons
  - 1. Read in the data
  - 3. Bray-Curtis and Weighted UniFrac Models Using brms
    - 3.1 Model
    - 3.2 Compare distribution of response variable to distributions of predicted response variable values
    - 3.3 Model diagnostics
    - 3.4 Plot model results for species comparison
    - 3.5. Plot model results for remaining predictors
  - 4. Bray-Curtis and Weighted UniFrac Models Using brms: Adult Individuals
    - 4.1 Model
    - 4.2 Compare distribution of response variable to distributions of predicted response variable values
    - 4.3 Model diagnostics
    - 4.4 Plot model results for species comparisson
    - 4.5. Plot model results for remaining predictors
  - 5. Bray-Curtis and Weighted UniFrac Models Using brms: Juvenile Individuals
    - 5.1 Model
    - 5.2 Compare distribution of response variable to distributions of predicted response variable values
    - 5.3 Model diagnostics
    - 5.4 Plot model results for species comparison
    - 5.5. Plot model results for remaining predictors
- C) Spacial distance and compositional differences

A) PERMANOVA

1. Read in the data

```
# Load libraries
library(qiime2R)
library(lubridate)
library(openxlsx)
library(phyloseq)
library(tidyverse)
library(microbiome)
library(metagenomeSeq)
library(vegan)
library(ggplot2)
library(tidyr)
```

```r
# Create phyloseq object
ps <- qza_to_phyloseq(
  features="table-final.qza",
  tree="rooted-tree.qza",
  taxonomy = "taxonomy.qza",
  metadata = "plover_metadata.tsv")

# Remove individuals with more than one sampling point
duplicates <- duplicated(sample_data(ps)$ring_number)
ps <- prune_samples(!duplicates, ps)

#Filter ps object, remove samples with no sex assignment, remove samples from nests with only one individual (improve sample set balance for permanova)

# remove samples with no sex
ps <- subset_samples(ps, !is.na(sample_data(ps)$sex) & sample_data(ps)$sex != "")#remove samples with no sex assignement

#remove samples from nests with only one individual
duplicate_nest <- duplicated(sample_data(ps)$nest) | duplicated(sample_data(ps)$nest, fromLast = TRUE)
ps <- subset_samples(ps, duplicate_nest)

summarize_phyloseq(ps) # summary of ps object

# Find samples with zero counts
zero_counts_samples <- sample_sums(otu_table(ps)) == 0
# Remove samples with zero counts
ps <- prune_samples(!zero_counts_samples, ps)
ps <- prune_taxa(taxa_sums(otu_table(ps)) > 1, ps) # remove singletons

saveRDS(ps, "phyloseq-plover,rds")
```

2. CSS tranformation

```r
# Convert the phyloseq object to a metagenomeSeq object (MRexperiment)
meta.obj <- phyloseq_to_metagenomeSeq(ps)

# Normalise counts
meta.obj <- cumNorm(meta.obj, p = cumNormStatFast(meta.obj))

# Convert CSS data into data.frame-formatted OTU table (log transformed data)
asv_table_css <- MRcounts(meta.obj, norm = TRUE, log = TRUE)

# Make a new phyloseq object with with the new CSS transformed ASV table
asv_table_css <- otu_table(asv_table_css, taxa_are_rows = TRUE)

taxonomy <- tax_table(ps)
taxonomy <- tax_table(taxonomy)

metadata <- sample_data(ps)

tree <- phy_tree(ps)
tree <- phy_tree(tree)

ps_css <- phyloseq(asv_table_css, taxonomy, metadata, tree)
```

```
saveRDS(ps_css, "ps_css.rds")
```

3. Perform PERMANOVA on Bray-Curtis dissimilarities and Weighted-UniFrac distances

```
# Model beta diversity

# compute beta diversity metrics
dist_bc <- distance(ps_css, method = "bray")
dist_wu <- distance(ps_css, method = "wunifrac")

#set permutation scheme (nest ID as a random effect)
perm <- how(nperm = 10000)
set.seed(1234)
setBlocks(perm) <- with(metadata, nest)


# PERMANOVA - Bray-curtis
> bc_perm <- adonis2(dist_bc ~ species + sex + age + year, data = metadata, permutations=perm1,
by= "margin")
> bc_perm

Permutation test for adonis under reduced model
Marginal effects of terms
Blocks:  with(metadata, nest)
Permutation: free
Number of permutations: 10000

adonis2(formula = dist_bc ~ species + sex + age + year, data = metadata, permutations = perm1,
by = "margin")
          Df SumOfSqs      R2      F  Pr(>F)
species    2    1.245 0.01932 1.3129 0.97560
sex        1    0.476 0.00739 1.0041 0.35756
age        1    0.511 0.00793 1.0779 0.05739 .
year       1    0.532 0.00825 1.1214 0.60224
Residual 130   61.652 0.95662
Total    135   64.448 1.00000
---
Signif. codes:  0 '***' 0.001 '**' 0.01 '*' 0.05 '.' 0.1 ' ' 1

## Analysis of homogeneity of group dispersions (variances) - Bray-Curtis
bc_betadisp <-betadisper(dist_bc, metadata$species)
> permutest(bc_betadisp_species, pairwise = TRUE, permutations = perm1)

Permutation test for homogeneity of multivariate dispersions
Blocks:  with(metadata, nest)
Permutation: free
Number of permutations: 10000

Response: Distances
           Df    Sum Sq    Mean Sq      F N.Perm    Pr(>F)
Groups      2 0.0060822 0.00304109 22.908  10000 9.999e-05 ***
Residuals 133 0.0176563 0.00013275
---
```

```
Signif. codes:  0 ‘***’ 0.001 ‘**’ 0.01 ‘*’ 0.05 ‘.’ 0.1 ‘ ’ 1

Pairwise comparisons:
(Observed p-value below diagonal, permuted p-value above diagonal)
            Cmarginatus Cpecuarius Cthoracicus
Cmarginatus                  1           1
Cpecuarius   0.774292                    1
Cthoracicus  1.3961e-06 1.1487e-08


# PERMANOVA - Wheighted UniFrac
> wu_perm <- adonis2(dist_wu ~ species + sex + age + year, data = metadata, permutations=perm1,
by= "margin")
> wu_perm

Permutation test for adonis under reduced model
Marginal effects of terms
Blocks:  with(metadata, nest)
Permutation: free
Number of permutations: 10000

adonis2(formula = dist_wu ~ species + sex + age + year, data = metadata, permutations = perm1,
by = "margin")
          Df SumOfSqs      R2      F Pr(>F)
species    2 0.008724 0.06643 4.7304 0.9808
sex        1 0.001159 0.00882 1.2565 0.1770
age        1 0.000553 0.00421 0.5992 0.6562
year       1 0.000726 0.00553 0.7872 0.0109 *
Residual 130 0.119879 0.91275
Total    135 0.131337 1.00000
---
Signif. codes:  0 ‘***’ 0.001 ‘**’ 0.01 ‘*’ 0.05 ‘.’ 0.1 ‘ ’ 1

## Analysis of homogeneity of group dispersions (variances) - Weighted UniFrac
wu_betadisp <-betadisper(dist_wu, metadata$species)
> permutest(wu_betadisp, pairwise = TRUE, permutations = 9999)

Permutation test for homogeneity of multivariate dispersions
Blocks:  with(metadata, nest)
Permutation: free
Number of permutations: 10000

Response: Distances
           Df    Sum Sq    Mean Sq     F N.Perm    Pr(>F)
Groups      2 0.0006511 0.00032553 3.524  10000 9.999e-05 ***
Residuals 133 0.0122860 0.00009238
---
Signif. codes:  0 ‘***’ 0.001 ‘**’ 0.01 ‘*’ 0.05 ‘.’ 0.1 ‘ ’ 1

Pairwise comparisons:
(Observed p-value below diagonal, permuted p-value above diagonal)
            Cmarginatus Cpecuarius Cthoracicus
Cmarginatus               1.000000           1
Cpecuarius     0.451733                      1
Cthoracicus    0.016428   0.015003


# Plot Bray-Curtis
data_pcoa_bray_css  <- ordinate(physeq = ps_css, method = "PCoA", distance = "bray") # compute
pcoa
```

```
species_colors <- c("black", "#B84123", "#60B4E9")
bc_species_age <- plot_ordination(physeq = ps_css, ordination = data_pcoa_bray_css, color =
"species",shape = "age", title = "BC PCOA species")
bc_species_age + geom_point(size=5)+scale_color_manual(values = species_colors) +
theme_classic()+
  theme(axis.text.x = element_text(angle = 45, hjust = 1),
        legend.position = "right") +
  theme(axis.text.x = element_text(angle = 45, hjust = 1, size = 14, family = "Arial"),
        axis.text.y = element_text(size = 14, family = "Arial"),
        axis.title.x = element_text(size = 14, family = "Arial"),
        axis.title.y = element_text(size = 14, family = "Arial"),
        legend.position = "right",
        legend.text = element_text(size = 14, family = "Arial"),
        legend.title = element_text(size = 14, family = "Arial"),
        plot.title = element_text(size = 20, family = "Arial"))

# Plot Wheighted UniFrac
data_pcoa_wu_css  <- ordinate(physeq = ps_css, method = "PCoA", distance = "wunifrac") #
compute pcoa

wu_species_age <- plot_ordination(physeq = ps_css, ordination = data_pcoa_wu_css, color =
"species",shape = "age", title = "WU PCOA species")
wu_species_age + geom_point(size=5)+scale_color_manual(values = species_colors) +
theme_classic()+
  theme(axis.text.x = element_text(angle = 45, hjust = 1),
        legend.position = "right") +
  theme(axis.text.x = element_text(angle = 45, hjust = 1, size = 14, family = "Arial"),
        axis.text.y = element_text(size = 14, family = "Arial"),
        axis.title.x = element_text(size = 14, family = "Arial"),
        axis.title.y = element_text(size = 14, family = "Arial"),
        legend.position = "right",
        legend.text = element_text(size = 14, family = "Arial"),
        legend.title = element_text(size = 14, family = "Arial"),
        plot.title = element_text(size = 20, family = "Arial"))
```

4. Perform PERMANOVA on Bray-Curtis dissimilarities and Weighted-UniFrac distances - Adult individuals only

```
# Model beta diversity by age - adults

# subset phyloseq object
ps_adults <- subset_samples(ps_css, age == "A")
metadata_adults <- data.frame(sample_data(ps_adults))

# compute beta diversity metrics
dist_bc_adults <- distance(ps_adults, method = "bray")
dist_wu_adults <- distance(ps_adults, method = "wunifrac")

#set permutation scheme (nest ID as a random effect)
perm_adults <- how(nperm = 10000)
set.seed(1234)
setBlocks(perm_adults) <- with(metadata_adults, nest)
```

```
# PERMANOVA - Bray-curtis
> bc_perm_adults <- adonis2(dist_bc_adults ~ species + sex + year, data = metadata_adults,
permutations=perm_adults, by= "margin")
> bc_perm_adults

Permutation test for adonis under reduced model
Marginal effects of terms
Blocks:  with(metadata_adults, nest)
Permutation: free
Number of permutations: 10000

adonis2(formula = dist_bc_adults ~ species + sex + year, data = metadata_adults, permutations =
perm_adults, by = "margin")
         Df SumOfSqs      R2      F Pr(>F)
species   2   1.0701 0.03511 1.1298 0.2410
sex       1   0.4757 0.01561 1.0045 0.2587
year      1   0.4964 0.01629 1.0482 0.9199
Residual 60  28.4128 0.93218
Total    64  30.4799 1.00000

## Analysis of homogeneity of group dispersions (variances) - Bray-Curtis
bc_adults_betadisp <-betadisper(dist_bc_adults, metadata_adults$species)
> permutest(bc_adults_betadisp, pairwise = TRUE, permutations = perm_adults)

Permutation test for homogeneity of multivariate dispersions
Blocks:  with(metadata_adults, nest)
Permutation: free
Number of permutations: 10000

Response: Distances
          Df    Sum Sq   Mean Sq      F N.Perm    Pr(>F)
Groups     2 0.0071217 0.0035608 20.062  10000 9.999e-05 ***
Residuals 62 0.0110043 0.0001775
---
Signif. codes:  0 '***' 0.001 '**' 0.01 '*' 0.05 '.' 0.1 ' ' 1

Pairwise comparisons:
(Observed p-value below diagonal, permuted p-value above diagonal)
            Cmarginatus Cpecuarius Cthoracicus
Cmarginatus                      1           1
Cpecuarius   6.8855e-01                      1
Cthoracicus  7.5076e-06 5.4942e-06           1


# PERMANOVA - Wheighted UniFrac
> wu_perm_adults <- adonis2(dist_wu_adults ~ species + sex + year, data = metadata_adults,
permutations=perm_adults, by= "margin")
> wu_perm_adults

Permutation test for adonis under reduced model
Marginal effects of terms
Blocks:  with(metadata_adults, nest)
Permutation: free
Number of permutations: 10000

adonis2(formula = dist_wu_adults ~ species + sex + year, data = metadata_adults, permutations =
perm_adults, by = "margin")
         Df SumOfSqs      R2      F Pr(>F)
species   2 0.002542 0.04294 1.3692 0.5230
sex       1 0.000570 0.00963 0.6143 0.8151
```

```
year      1 0.000400 0.00676 0.4310 0.7189
Residual 60 0.055704 0.94081
Total    64 0.059209 1.00000

## Analysis of homogeneity of group dispersions (variances) - Weighted UniFrac
wu_adults_betadisp <-betadisper(dist_wu_adults, metadata_adults$species)
> permutest(wu_adults_betadisp, pairwise = TRUE, permutations = perm_adults)

Permutation test for homogeneity of multivariate dispersions
Blocks:  with(metadata_adults, nest)
Permutation: free
Number of permutations: 10000

Response: Distances
          Df    Sum Sq   Mean Sq      F N.Perm    Pr(>F)
Groups     2 0.0009942 4.971e-04 6.0929  10000 9.999e-05 ***
Residuals 62 0.0050584 8.159e-05
---
Signif. codes:  0 ‘***’ 0.001 ‘**’ 0.01 ‘*’ 0.05 ‘.’ 0.1 ‘ ’ 1

Pairwise comparisons:
(Observed p-value below diagonal, permuted p-value above diagonal)
            Cmarginatus Cpecuarius Cthoracicus
Cmarginatus              1.0000000           1
Cpecuarius    0.2953976                      1
Cthoracicus   0.0027114  0.0026519


# Plot Bray-Curtis
species_colors <- c("black", "#B84123", "#60B4E9")

pcoa_bc_adults  <- ordinate(physeq = ps_adults, method = "PCoA", distance = "bray") # compute
pcoa

bc_species_adults <- plot_ordination(physeq = ps_adults, ordination = pcoa_bc_adults, color =
"species",shape = "sex", title = "BC PCOA species")
bc_species_adults + geom_point(size=5)+scale_color_manual(values = species_colors) +
theme_classic()+
  theme(axis.text.x = element_text(angle = 45, hjust = 1),
        legend.position = "right") +
  theme(axis.text.x = element_text(angle = 45, hjust = 1, size = 14, family = "Arial"),
        axis.text.y = element_text(size = 14, family = "Arial"),
        axis.title.x = element_text(size = 14, family = "Arial"),
        axis.title.y = element_text(size = 14, family = "Arial"),
        legend.position = "right",
        legend.text = element_text(size = 14, family = "Arial"),
        legend.title = element_text(size = 14, family = "Arial"),
        plot.title = element_text(size = 20, family = "Arial"))

# Plot Wheighted UniFrac
pcoa_wu_adults  <- ordinate(physeq = ps_adults, method = "PCoA", distance = "wunifrac") #
compute pcoa

wu_species_adults <- plot_ordination(physeq = ps_adults, ordination = pcoa_wu_adults, color =
"species",shape = "sex", title = "WU PCOA species")
wu_species_adults + geom_point(size=5)+scale_color_manual(values = species_colors) +
theme_classic()+
  theme(axis.text.x = element_text(angle = 45, hjust = 1),
        legend.position = "right") +
  theme(axis.text.x = element_text(angle = 45, hjust = 1, size = 14, family = "Arial"),
```

```
        axis.text.y = element_text(size = 14, family = "Arial"),
        axis.title.x = element_text(size = 14, family = "Arial"),
        axis.title.y = element_text(size = 14, family = "Arial"),
        legend.position = "right",
        legend.text = element_text(size = 14, family = "Arial"),
        legend.title = element_text(size = 14, family = "Arial"),
        plot.title = element_text(size = 20, family = "Arial"))
```

5. Perform PERMANOVA on Bray-Curtis dissimilarities and Weighted-UniFrac distances - Juveniles individuals only

```
# Model beta diversity by age - juveniles

# subset phyloseq object

ps_juv <- subset_samples(ps_css, age == "J")
metadata_juv <- data.frame(sample_data(ps_juv))

# compute beta diversity metrics

dist_bc_juv <- distance(ps_juv, method = "bray")
dist_wu_juv <- distance(ps_juv, method = "wunifrac")

#set permutation scheme (nest ID as a random effect)
perm_juv <- how(nperm = 10000)
set.seed(1234)
setBlocks(perm_juv) <- with(metadata_juv, nest)

# PERMANOVA - Bray-curtis
> bc_perm_juv <- adonis2(dist_bc_juv ~ species + sex + year, data = metadata_juv,
permutations=perm_juv, by= "margin")
> bc_perm_juv

Permutation test for adonis under reduced model
Marginal effects of terms
Blocks:  with(metadata_juv, nest)
Permutation: free
Number of permutations: 10000

adonis2(formula = dist_bc_juv ~ species + sex + year, data = metadata_juv, permutations =
perm_juv, by = "margin")
         Df SumOfSqs      R2      F Pr(>F)
species   2    1.178 0.03521 1.2421 0.8648
sex       1    0.455 0.01360 0.9597 0.8061
year      1    0.527 0.01576 1.1118 0.5111
Residual 66   31.294 0.93538
Total    70   33.455 1.00000

## Analysis of homogeneity of group dispersions (variances) - Bray-Curtis
bc_juv_betadisp <-betadisper(dist_bc_juv, metadata_juv$species)
> permutest(bc_juv_betadisp, pairwise = TRUE, permutations = perm_juv)

Permutation test for homogeneity of multivariate dispersions
Blocks:  with(metadata_juv, nest)
Permutation: free
Number of permutations: 10000
```

```
Response: Distances
          Df    Sum Sq   Mean Sq      F N.Perm    Pr(>F)
Groups     2 0.0112517 0.0056258 39.535  10000 9.999e-05 ***
Residuals 68 0.0096766 0.0001423
---
Signif. codes:  0 '***' 0.001 '**' 0.01 '*' 0.05 '.' 0.1 ' ' 1

Pairwise comparisons:
(Observed p-value below diagonal, permuted p-value above diagonal)
            Cmarginatus Cpecuarius Cthoracicus
Cmarginatus              1.0000e+00           1
Cpecuarius   9.1781e-01                       1
Cthoracicus  7.6587e-08 4.2679e-11


# PERMANOVA - Wheighted UniFrac
> wu_perm_juv <- adonis2(dist_wu_juv ~ species + sex + year, data = metadata_juv,
permutations=perm_juv, by= "margin")
> wu_perm_juv

Permutation test for adonis under reduced model
Marginal effects of terms
Blocks:  with(metadata_juv, nest)
Permutation: free
Number of permutations: 10000

adonis2(formula = dist_wu_juv ~ species + sex + year, data = metadata_juv, permutations =
perm_juv, by = "margin")
         Df SumOfSqs      R2      F Pr(>F)
species   2 0.008872 0.12399 4.9143 0.2495
sex       1 0.001929 0.02696 2.1370 0.2422
year      1 0.000940 0.01313 1.0410 0.2217
Residual 66 0.059574 0.83260
Total    70 0.071553 1.00000

## Analysis of homogeneity of group dispersions (variances) - Weighted UniFrac
wu_juv_betadisp <-betadisper(dist_wu_juv, metadata_juv$species)
> permutest(wu_juv_betadisp, pairwise = TRUE, permutations = perm_juv)
Permutation test for homogeneity of multivariate dispersions
Blocks:  with(metadata_juv, nest)
Permutation: free
Number of permutations: 10000

Response: Distances
          Df    Sum Sq    Mean Sq      F N.Perm    Pr(>F)
Groups     2 0.0002147 0.00010733 0.9303  10000 9.999e-05 ***
Residuals 68 0.0078451 0.00011537
---
Signif. codes:  0 '***' 0.001 '**' 0.01 '*' 0.05 '.' 0.1 ' ' 1

Pairwise comparisons:
(Observed p-value below diagonal, permuted p-value above diagonal)
            Cmarginatus Cpecuarius Cthoracicus
Cmarginatus                1.00000           1
Cpecuarius      0.86979                      1
Cthoracicus     0.13201    0.25954



# Plot Bray-Curtis
```

```
pcoa_bc_juv  <- ordinate(physeq = ps_juv, method = "PCoA", distance = "bray") # compute pcoa

bc_species_juv <- plot_ordination(physeq = ps_juv, ordination = pcoa_bc_juv, color =
"species",shape = "sex", title = "BC PCOA species")
bc_species_juv + geom_point(size=5)+scale_color_manual(values = species_colors) +
theme_classic()+
  theme(axis.text.x = element_text(angle = 45, hjust = 1),
        legend.position = "right") +
  theme(axis.text.x = element_text(angle = 45, hjust = 1, size = 14, family = "Arial"),
        axis.text.y = element_text(size = 14, family = "Arial"),
        axis.title.x = element_text(size = 14, family = "Arial"),
        axis.title.y = element_text(size = 14, family = "Arial"),
        legend.position = "right",
        legend.text = element_text(size = 14, family = "Arial"),
        legend.title = element_text(size = 14, family = "Arial"),
        plot.title = element_text(size = 20, family = "Arial"))


# Plot Wheighted UniFrac
pcoa_wu_juv  <- ordinate(physeq = ps_juv, method = "PCoA", distance = "wunifrac") # compute
pcoa

wu_species_juv <- plot_ordination(physeq = ps_juv, ordination = pcoa_wu_juv, color =
"species",shape = "sex", title = "WU PCOA species")
wu_species_juv + geom_point(size=5)+scale_color_manual(values = species_colors) +
theme_classic()+
  theme(axis.text.x = element_text(angle = 45, hjust = 1),
        legend.position = "right") +
  theme(axis.text.x = element_text(angle = 45, hjust = 1, size = 14, family = "Arial"),
        axis.text.y = element_text(size = 14, family = "Arial"),
        axis.title.x = element_text(size = 14, family = "Arial"),
        axis.title.y = element_text(size = 14, family = "Arial"),
        legend.position = "right",
        legend.text = element_text(size = 14, family = "Arial"),
        legend.title = element_text(size = 14, family = "Arial"),
        plot.title = element_text(size = 20, family = "Arial"))
```

B) Model pairwise comparisons

1. Read in the data

```
# Load libraries
library(brms)
library(rstan)
library(parallel)
library(bayesplot)
library(ggplot2)

# Read in the data
data.dyad_adults <- readRDS("data.dyad-adults.rds")

data.dyad_juv <- readRDS("data.dyad-juv.rds")
```

3. Bray-Curtis and Weighted UniFrac Models Using brms

3.1 Model

```
# Model BC dissimilarity

ncores = detectCores()
options(mc.cores = parallel::detectCores())

model_bc <- brm(BC~1+ species_combo + age_combo + sex_combo + nest + year + (1|mm(IDA,IDB)),
                data = data.dyad,
                family= "zero_one_inflated_beta",
                warmup = 25000, iter = 50000,
                cores = ncores, chains = 4,
                init=0)

saveRDS(model_bc, "model_bc.rds")

> summary(model_bc)

Family: zero_one_inflated_beta
  Links: mu = logit; phi = identity; zoi = identity; coi = identity
Formula: BC ~ 1 + species_combo + age_combo + sex_combo + nest + year + (1 | mm(IDA, IDB))
   Data: data.dyad (Number of observations: 9180)
  Draws: 4 chains, each with iter = 50000; warmup = 25000; thin = 1;
         total post-warmup draws = 1e+05

Group-Level Effects:
~mmIDAIDB (Number of levels: 136)
              Estimate Est.Error l-95% CI u-95% CI Rhat Bulk_ESS Tail_ESS
sd(Intercept)     0.65      0.04     0.58     0.74 1.00     5804    12883

Population-Level Effects:
                  Estimate Est.Error l-95% CI u-95% CI Rhat Bulk_ESS Tail_ESS
Intercept             3.83      0.12     3.59     4.08 1.00     3193     7151
species_comboCMCT    -0.14      0.09    -0.32     0.03 1.00     3647     7968
species_comboCPCM     0.02      0.06    -0.10     0.15 1.00     3076     7058
species_comboCPCP    -0.13      0.12    -0.37     0.11 1.00     2920     6426
species_comboCPCT    -0.08      0.13    -0.33     0.16 1.00     3091     6593
species_comboCTCT    -0.44      0.18    -0.79    -0.08 1.00     3608     8145
age_comboAJ           0.03      0.06    -0.08     0.15 1.00     3120     6230
age_comboJJ           0.01      0.11    -0.21     0.23 1.00     2990     5912
sex_comboFM           0.05      0.06    -0.07     0.17 1.00     2926     7306
sex_comboMM           0.08      0.12    -0.16     0.31 1.00     2777     6856
nest1                -0.16      0.05    -0.26    -0.06 1.00    69067    71755
```

```
year1                -0.02      0.01    -0.05     0.00 1.00    59919    68978

Family Specific Parameters:
    Estimate Est.Error l-95% CI u-95% CI Rhat Bulk_ESS Tail_ESS
phi   111.40      1.76   107.99   114.87 1.00    67087    72307
zoi     0.02      0.00     0.01     0.02 1.00    68658    68986
coi     0.99      0.01     0.98     1.00 1.00    57655    36912



# Perform hypothesis testing for the remaining species comaprisons

> hypothesis(model_bc, "Intercept + species_comboAPAP = Intercept + species_comboAPAT") # APAP
vs APAT
 Hypothesis Tests for class b:
                Hypothesis Estimate Est.Error CI.Lower CI.Upper Evid.Ratio Post.Prob Star
1 (Intercept+specie... = 0    -0.05      0.09    -0.22     0.13         NA        NA

> hypothesis(model_bc, "Intercept + species_comboAPAP = Intercept + species_comboAPAM") # APAP
vs APAM
 Hypothesis Tests for class b:
                Hypothesis Estimate Est.Error CI.Lower CI.Upper Evid.Ratio Post.Prob Star
1 (Intercept+specie... = 0    -0.16      0.06    -0.28    -0.03         NA        NA    *

> hypothesis(model_bc, "Intercept + species_comboATAT = Intercept + species_comboAPAT") #  ATAT
vs ATAP
Hypothesis Tests for class b:
                Hypothesis Estimate Est.Error CI.Lower CI.Upper Evid.Ratio Post.Prob Star
1 (Intercept+specie... = 0    -0.35       0.1    -0.54    -0.17         NA        NA    *

> hypothesis(model_bc, "Intercept + species_comboATAT = Intercept + species_comboAMAT") #  ATAT
vs ATAM
   Hypothesis Tests for class b:
                Hypothesis Estimate Est.Error CI.Lower CI.Upper Evid.Ratio Post.Prob Star
1 (Intercept+specie... = 0    -0.29       0.1    -0.48     -0.1         NA        NA    *

> hypothesis(model_bc, "Intercept + species_comboATAT = Intercept + species_comboAPAP") #  ATAT
vs APAP
   Hypothesis Tests for class b:
                Hypothesis Estimate Est.Error CI.Lower CI.Upper Evid.Ratio Post.Prob Star
1 (Intercept+specie... = 0    -0.31      0.18    -0.65     0.04         NA        NA



# Model Wheighted-UniFrac distances
ncores = detectCores()
options(mc.cores = parallel::detectCores())

model_WU_all_combo <- brm(WU~1+ species_combo + age_combo + sex_combo + nest + year +
(1|mm(IDA,IDB)),
                data = data.dyad,
                family= "Beta",
                warmup = 25000, iter = 50000,
                cores = ncores, chains = 4,
                init=0)

saveRDS(model_wu, "model_wu.rds")

> summary(model_wu)
```

```
   Family: beta
  Links: mu = logit; phi = identity
Formula: WU ~ 1 + species_combo + age_combo + sex_combo + nest + year + (1 | mm(IDA, IDB))
   Data: data.dyad (Number of observations: 9180)
  Draws: 4 chains, each with iter = 50000; warmup = 25000; thin = 1;
         total post-warmup draws = 1e+05

Group-Level Effects:
~mmIDAIDB (Number of levels: 136)
              Estimate Est.Error l-95% CI u-95% CI Rhat Bulk_ESS Tail_ESS
sd(Intercept)     0.25      0.02     0.22     0.28 1.00    11509    22842

Population-Level Effects:
                  Estimate Est.Error l-95% CI u-95% CI Rhat Bulk_ESS Tail_ESS
Intercept            -3.20      0.05    -3.30    -3.10 1.00     6336    13291
species_comboCMCT     0.00      0.04    -0.07     0.07 1.00     7667    16356
species_comboCPCM     0.03      0.03    -0.02     0.08 1.00     6431    14432
species_comboCPCP     0.03      0.05    -0.06     0.13 1.00     6048    12994
species_comboCPCT     0.05      0.05    -0.05     0.14 1.00     6236    13557
species_comboCTCT    -0.20      0.07    -0.34    -0.06 1.00     7763    16665
age_comboAJ           0.02      0.02    -0.03     0.06 1.00     6540    15047
age_comboJJ           0.04      0.04    -0.04     0.13 1.00     6148    13441
sex_comboFM           0.01      0.02    -0.03     0.06 1.00     6925    15745
sex_comboMM           0.03      0.05    -0.06     0.12 1.00     6415    14226
nest1                -0.03      0.03    -0.08     0.02 1.00   126055    75122
year1                 0.00      0.01    -0.01     0.02 1.00   103749    81249

Family Specific Parameters:
    Estimate Est.Error l-95% CI u-95% CI Rhat Bulk_ESS Tail_ESS
phi   312.27      4.68   303.20   321.46 1.00   129232    76688



# Perform hypothesis testing for the remaining species comaprisons

> hypothesis(model_wu, "Intercept + species_comboAPAP = Intercept + species_comboAPAT") # APAP
vs APAT
   Hypothesis Tests for class b:
                Hypothesis Estimate Est.Error CI.Lower CI.Upper Evid.Ratio Post.Prob Star
1 (Intercept+specie... = 0    -0.02      0.03    -0.08     0.05         NA        NA

> hypothesis(model_wu, "Intercept + species_comboAPAP = Intercept + species_comboAPAM") # APAP
vs APAM
 Hypothesis Tests for class b:
                Hypothesis Estimate Est.Error CI.Lower CI.Upper Evid.Ratio Post.Prob Star
1 (Intercept+specie... = 0        0      0.02    -0.05     0.05         NA        NA

> hypothesis(model_wu, "Intercept + species_comboATAT = Intercept + species_comboAPAT") #  ATAT
vs ATAP
Hypothesis Tests for class b:
                Hypothesis Estimate Est.Error CI.Lower CI.Upper Evid.Ratio Post.Prob Star
1 (Intercept+specie... = 0    -0.25      0.04    -0.33    -0.17         NA        NA    *

> hypothesis(model_wu, "Intercept + species_comboATAT = Intercept + species_comboAMAT") #  ATAT
vs ATAM
   Hypothesis Tests for class b:
                Hypothesis Estimate Est.Error CI.Lower CI.Upper Evid.Ratio Post.Prob Star
1 (Intercept+specie... = 0     -0.2      0.04    -0.28    -0.12         NA        NA    *
```

```
> hypothesis(model_wu, "Intercept + species_comboATAT = Intercept + species_comboAPAP") #  ATAT
vs APAP
   Hypothesis Tests for class b:
                Hypothesis Estimate Est.Error CI.Lower CI.Upper Evid.Ratio Post.Prob Star
1 (Intercept+specie... = 0     -0.2      0.04    -0.28    -0.12         NA        NA    *
```

3.2 Compare distribution of response variable to distributions of predicted response variable values

```
pp_check(model_bc, ndraws = 100)

pp_check(model_wu, ndraws = 100)
```

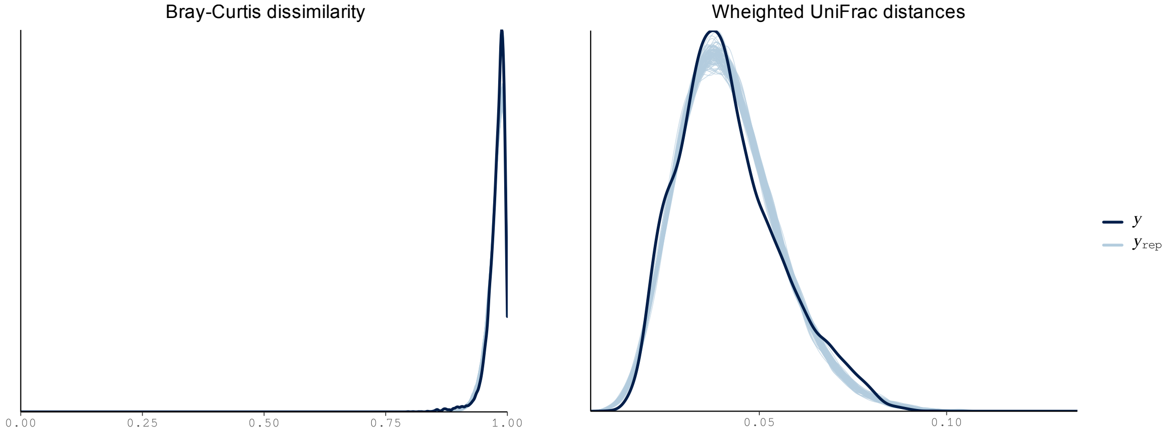

3.3 Model diagnostics

```
# BC model diagnostics
plot(model_bc)
```

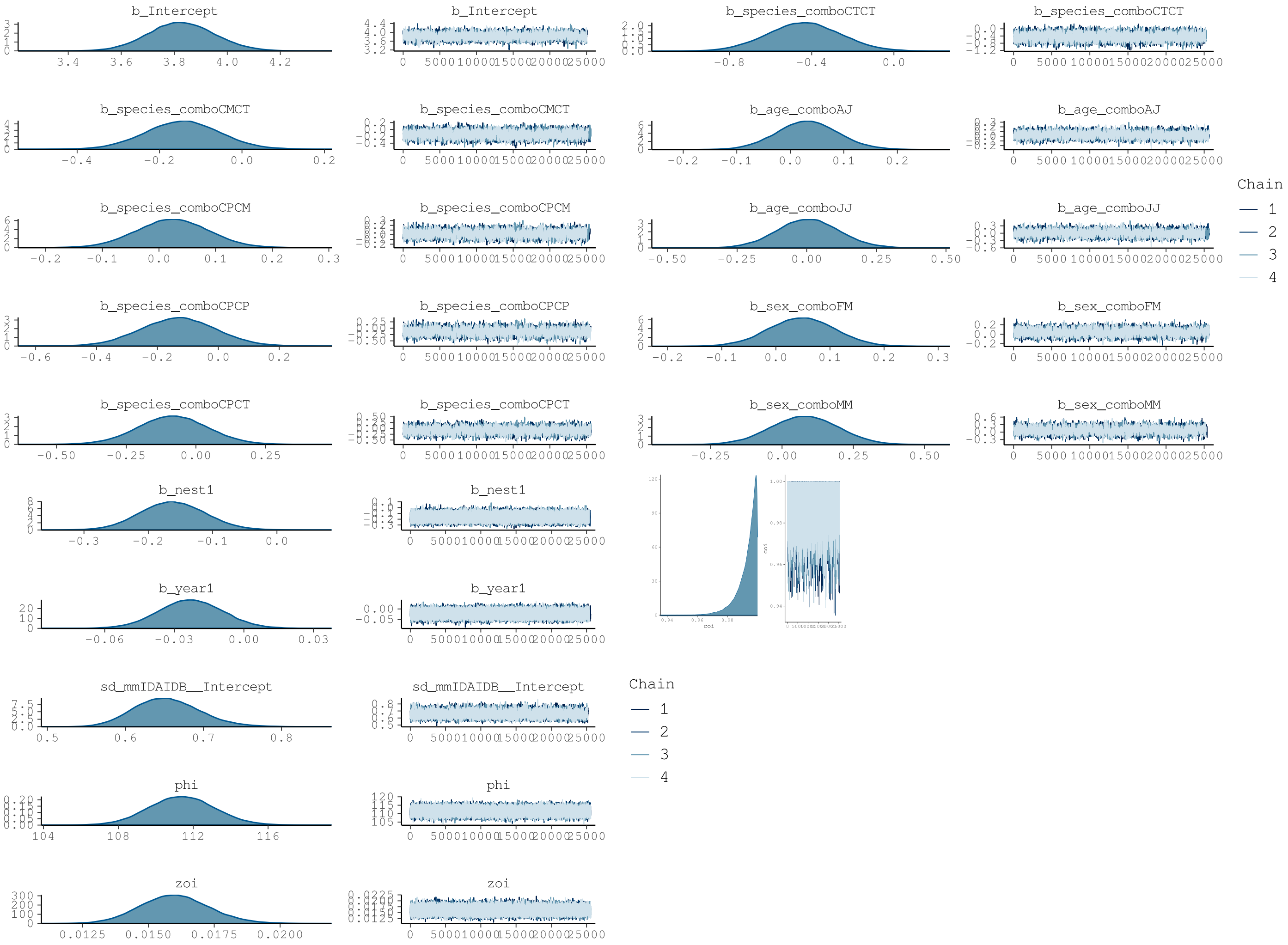


```r
# WU model diagnostics
plot(model_wu)
```

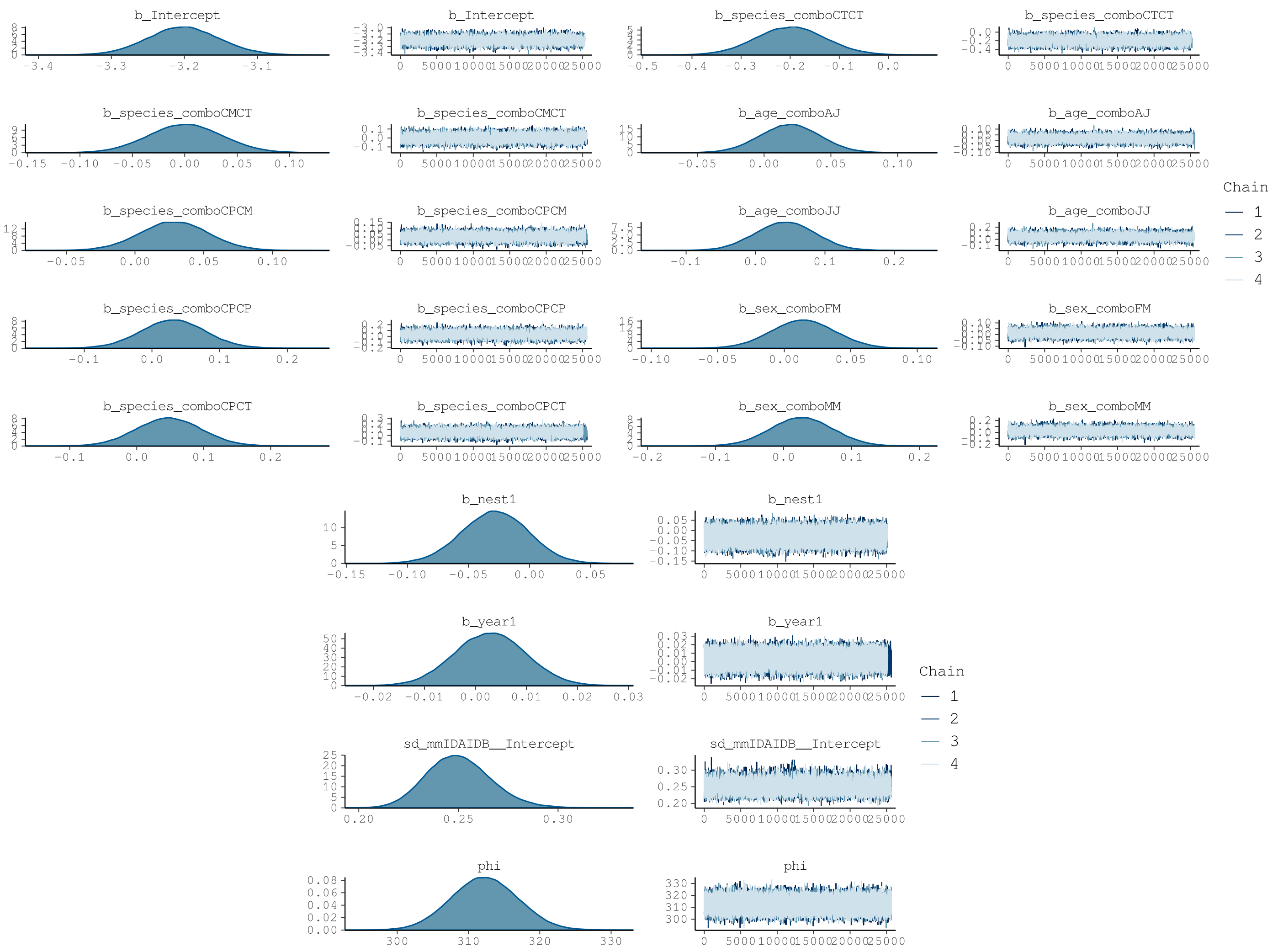

3.4 Plot model results for species comparison

```
# Bray-Curtis
effect_species <- conditional_effects(model_bc, effects="species_combo") # Default: doesn't
include random effects

plot(effect_species)[[1]] + theme_classic()+
  theme(axis.text.x = element_text(angle = 45, hjust = 1),
        legend.position = "right") +
  theme(axis.text.x = element_text(angle = 45, hjust = 1, size = 14, family = "Arial"),
        axis.text.y = element_text(size = 14, family = "Arial"),
        axis.title.x = element_text(size = 14, family = "Arial"),
        axis.title.y = element_text(size = 14, family = "Arial"),
        legend.position = "right",
        legend.text = element_text(size = 14, family = "Arial"),
        legend.title = element_text(size = 14, family = "Arial"),
        plot.title = element_text(size = 20, family = "Arial"))

# Wheighted UniFrac
effect_species <- conditional_effects(model_wu, effects="species_combo") # Default: doesn't
include random effects

plot(effect_species)[[1]] + theme_classic()+
  theme(axis.text.x = element_text(angle = 45, hjust = 1),
        legend.position = "right") +
  theme(axis.text.x = element_text(angle = 45, hjust = 1, size = 14, family = "Arial"),
        axis.text.y = element_text(size = 14, family = "Arial"),
        axis.title.x = element_text(size = 14, family = "Arial"),
```

```
        axis.title.y = element_text(size = 14, family = "Arial"),
        legend.position = "right",
        legend.text = element_text(size = 14, family = "Arial"),
        legend.title = element_text(size = 14, family = "Arial"),
        plot.title = element_text(size = 20, family = "Arial"))
```

3.5. Plot model results for remaining predictors

```
# Bray-Curtis
plot_bc <-mcmc_plot(model_bc, type = "intervals", prob_outer=0.95, prob=0.95,
                  variable =c("b_age_comboAJ", "b_age_comboJJ", "b_sex_comboFM",
"b_sex_comboMM", "b_nest1","b_year1"))
plot_bc <- plot1 + theme_classic() + geom_vline(xintercept = 0, linetype="dotted",
color="blue")+
  theme(axis.text.x = element_text(size = 16),  # Adjust the size as needed
        axis.text.y = element_text(size = 16))+
  theme(text = element_text(family = "Arial"))
plot_bc


# Wheighted UniFrac
plot_wu <-mcmc_plot(model_wu, type = "intervals", prob_outer=0.95, prob=0.95,
                  variable =c("b_age_comboAJ", "b_age_comboJJ", "b_sex_comboFM",
"b_sex_comboMM", "b_nest1","b_year1"))
plot_wu <- plot1 + theme_classic() + geom_vline(xintercept = 0, linetype="dotted",
color="blue")+
  theme(axis.text.x = element_text(size = 16),  # Adjust the size as needed
        axis.text.y = element_text(size = 16))+
  theme(text = element_text(family = "Arial"))
plot_wu
```

4. Bray-Curtis and Weighted UniFrac Models Using brms: Adult Individuals

4.1 Model

```
# Model BC dissimilarity

ncores = detectCores()
options(mc.cores = parallel::detectCores())

model_bc_adults <- brm(BC~1+ species_combo + sex_combo + nest + year + (1|mm(IDA,IDB)),
                data = data.dyad_adults,
                family= "zero_one_inflated_beta",
                warmup = 25000, iter = 50000,
                cores = ncores, chains = 4,
                init=0)

saveRDS(model_bc_adults, "model_bc_adults.rds")

> summary(model_bc_adults)

Family: zero_one_inflated_beta
  Links: mu = logit; phi = identity; zoi = identity; coi = identity
Formula: BC ~ 1 + species_combo + sex_combo + nest + year + (1 | mm(IDA, IDB))
   Data: data.dyad (Number of observations: 2080)
```

```
 Draws: 4 chains, each with iter = 50000; warmup = 25000; thin = 1;
        total post-warmup draws = 1e+05

Group-Level Effects:
~mmIDAIDB (Number of levels: 65)
              Estimate Est.Error l-95% CI u-95% CI Rhat Bulk_ESS Tail_ESS
sd(Intercept)     0.79      0.08     0.65     0.95 1.00    12168    25963

Population-Level Effects:
                  Estimate Est.Error l-95% CI u-95% CI Rhat Bulk_ESS Tail_ESS
Intercept             3.81      0.19     3.43     4.18 1.00     9107    16882
species_comboAMAT    -0.21      0.15    -0.51     0.09 1.00     9465    19605
species_comboAPAM    -0.00      0.12    -0.24     0.23 1.00     8119    17795
species_comboAPAP    -0.18      0.23    -0.63     0.27 1.00     7759    15888
species_comboAPAT    -0.28      0.22    -0.71     0.16 1.00     7864    16291
species_comboATAT    -0.47      0.30    -1.07     0.13 1.00     9287    19256
sex_comboFM           0.10      0.11    -0.12     0.32 1.00     9664    19758
sex_comboMM           0.17      0.22    -0.25     0.60 1.00     9116    17498
nest1                -0.27      0.14    -0.52     0.01 1.00    92670    67649
year1                 0.00      0.03    -0.05     0.06 1.00    89250    76748

Family Specific Parameters:
    Estimate Est.Error l-95% CI u-95% CI Rhat Bulk_ESS Tail_ESS
phi   104.95      3.54    98.15   112.04 1.00    87305    77148
zoi     0.02      0.00     0.01     0.03 1.00    97298    71307
coi     0.98      0.02     0.92     1.00 1.00    76879    40710



# Perform hypothesis testing for the remaining species comaprisons

> hypothesis(model_bc_adults, "Intercept + species_comboAPAP = Intercept + species_comboAPAT")
# APAP vs APAT
Hypothesis Tests for class b:
                Hypothesis Estimate Est.Error CI.Lower CI.Upper Evid.Ratio Post.Prob Star
1 (Intercept+specie... = 0      0.1      0.15     -0.2      0.4         NA        NA

> hypothesis(model_bc_adults, "Intercept + species_comboAPAP = Intercept + species_comboAPAM")
# APAP vs APAM
Hypothesis Tests for class b:
                Hypothesis Estimate Est.Error CI.Lower CI.Upper Evid.Ratio Post.Prob Star
1 (Intercept+specie... = 0    -0.17      0.12    -0.41     0.06         NA        NA

> hypothesis(model_bc_adults, "Intercept + species_comboATAT = Intercept + species_comboAPAT")
#  ATAT vs ATAP
Hypothesis Tests for class b:
                Hypothesis Estimate Est.Error CI.Lower CI.Upper Evid.Ratio Post.Prob Star
1 (Intercept+specie... = 0     -0.2      0.17    -0.52     0.13         NA        NA

> hypothesis(model_bc_adults, "Intercept + species_comboATAT = Intercept + species_comboAMAT")
#  ATAT vs ATAM
Hypothesis Tests for class b:
                Hypothesis Estimate Est.Error CI.Lower CI.Upper Evid.Ratio Post.Prob Star
1 (Intercept+specie... = 0    -0.26      0.17    -0.59     0.07         NA        NA

> hypothesis(model_bc_adults, "Intercept + species_comboATAT = Intercept + species_comboAPAP")
#  ATAT vs APAP
Hypothesis Tests for class b:
                Hypothesis Estimate Est.Error CI.Lower CI.Upper Evid.Ratio Post.Prob Star
1 (Intercept+specie... = 0     -0.3       0.3    -0.89      0.3         NA        NA
```

```
# Model Wheighted-UniFrac distances

ncores = detectCores()
options(mc.cores = parallel::detectCores())

model_wu_adutls <- brm(WU~1+ species_combo + + sex_combo + nest + year + (1|mm(IDA,IDB)),
                data = data.dyad_adults,
                family= "Beta",
                warmup = 25000, iter = 50000,
                cores = ncores, chains = 4,
                init=0)

saveRDS(model_wu_adults, "model_wu_adults.rds")

> summary(model_wu_adults)

Family: beta
  Links: mu = logit; phi = identity
Formula: WU ~ 1 + species_combo + +sex_combo + nest + year + (1 | mm(IDA, IDB))
   Data: data.dyad (Number of observations: 2080)
  Draws: 4 chains, each with iter = 50000; warmup = 25000; thin = 1;
         total post-warmup draws = 1e+05

Group-Level Effects:
~mmIDAIDB (Number of levels: 65)
              Estimate Est.Error l-95% CI u-95% CI Rhat Bulk_ESS Tail_ESS
sd(Intercept)     0.26      0.03     0.21     0.31 1.00    19262    29629

Population-Level Effects:
                  Estimate Est.Error l-95% CI u-95% CI Rhat Bulk_ESS Tail_ESS
Intercept            -3.22      0.06    -3.34    -3.09 1.00    16100    29541
species_comboAMAT    -0.11      0.05    -0.21    -0.00 1.00    17995    34755
species_comboAPAM     0.03      0.04    -0.05     0.11 1.00    14985    30504
species_comboAPAP     0.04      0.08    -0.11     0.19 1.00    13756    26529
species_comboAPAT    -0.07      0.07    -0.22     0.07 1.00    14244    27361
species_comboATAT    -0.33      0.11    -0.54    -0.11 1.00    18451    35741
sex_comboFM           0.04      0.04    -0.04     0.12 1.00    17739    33346
sex_comboMM           0.08      0.07    -0.06     0.23 1.00    15643    28947
nest1                -0.16      0.08    -0.32    -0.01 1.00   138942    69779
year1                 0.02      0.01    -0.01     0.04 1.00   121391    77677

Family Specific Parameters:
    Estimate Est.Error l-95% CI u-95% CI Rhat Bulk_ESS Tail_ESS
phi   352.51     11.16   330.96   374.81 1.00   134420    71543


# Perform hypothesis testing for the remaining species comaprisons
> hypothesis(model_wu_adults, "Intercept + species_comboAPAP = Intercept + species_comboAPAT")
# APAP vs APAT
Hypothesis Tests for class b:
                Hypothesis Estimate Est.Error CI.Lower CI.Upper Evid.Ratio Post.Prob Star
1 (Intercept+specie... = 0     0.11      0.05     0.01     0.21         NA        NA    *

> hypothesis(model_wu_adults, "Intercept + species_comboAPAP = Intercept + species_comboAPAM")
# APAP vs APAM
Hypothesis Tests for class b:
                Hypothesis Estimate Est.Error CI.Lower CI.Upper Evid.Ratio Post.Prob Star
```

```
1 (Intercept+specie... = 0      0.01      0.04     -0.07      0.09          NA         NA

> hypothesis(model_wu_adults, "Intercept + species_comboATAT = Intercept + species_comboAPAT")
#  ATAT vs ATAP
Hypothesis Tests for class b:
                Hypothesis Estimate Est.Error CI.Lower CI.Upper Evid.Ratio Post.Prob Star
1 (Intercept+specie... = 0    -0.25      0.07     -0.39    -0.12          NA        NA    *

> hypothesis(model_wu_adults, "Intercept + species_comboATAT = Intercept + species_comboAMAT")
#  ATAT vs ATAM
Hypothesis Tests for class b:
                Hypothesis Estimate Est.Error CI.Lower CI.Upper Evid.Ratio Post.Prob Star
1 (Intercept+specie... = 0    -0.22      0.07     -0.36    -0.08          NA        NA    *

> hypothesis(model_wu_adults, "Intercept + species_comboATAT = Intercept + species_comboAPAP")
#  ATAT vs APAP
Hypothesis Tests for class b:
                Hypothesis Estimate Est.Error CI.Lower CI.Upper Evid.Ratio Post.Prob Star
1 (Intercept+specie... = 0    -0.37      0.11     -0.58    -0.15          NA        NA    *
```

4.2 Compare distribution of response variable to distributions of predicted response variable values

```
pp_check(model_bc_adults, ndraws = 100)

pp_check(model_wu_adults, ndraws = 100)
```

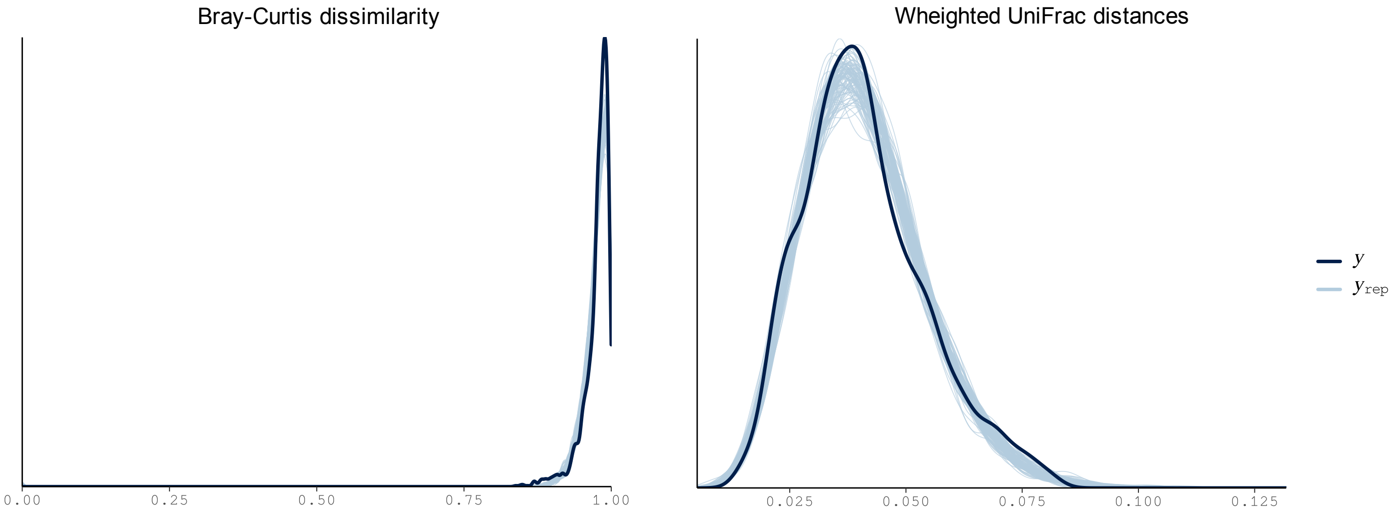

4.3 Model diagnostics

```
# BC model diagnostics
plot(model_bc_adults)
```

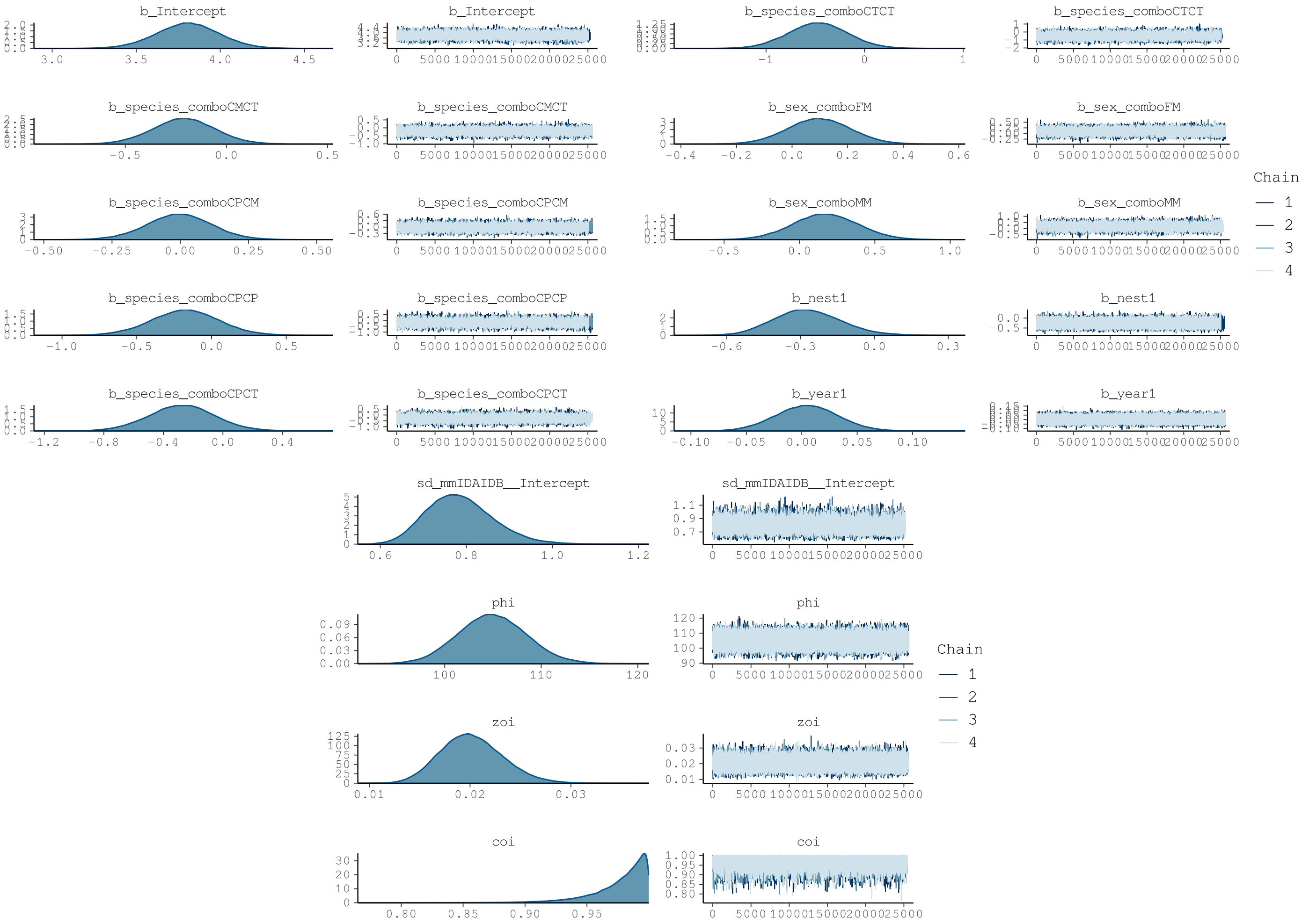


```r
# WU model diagnostics

plot(model_wu_adults)
```

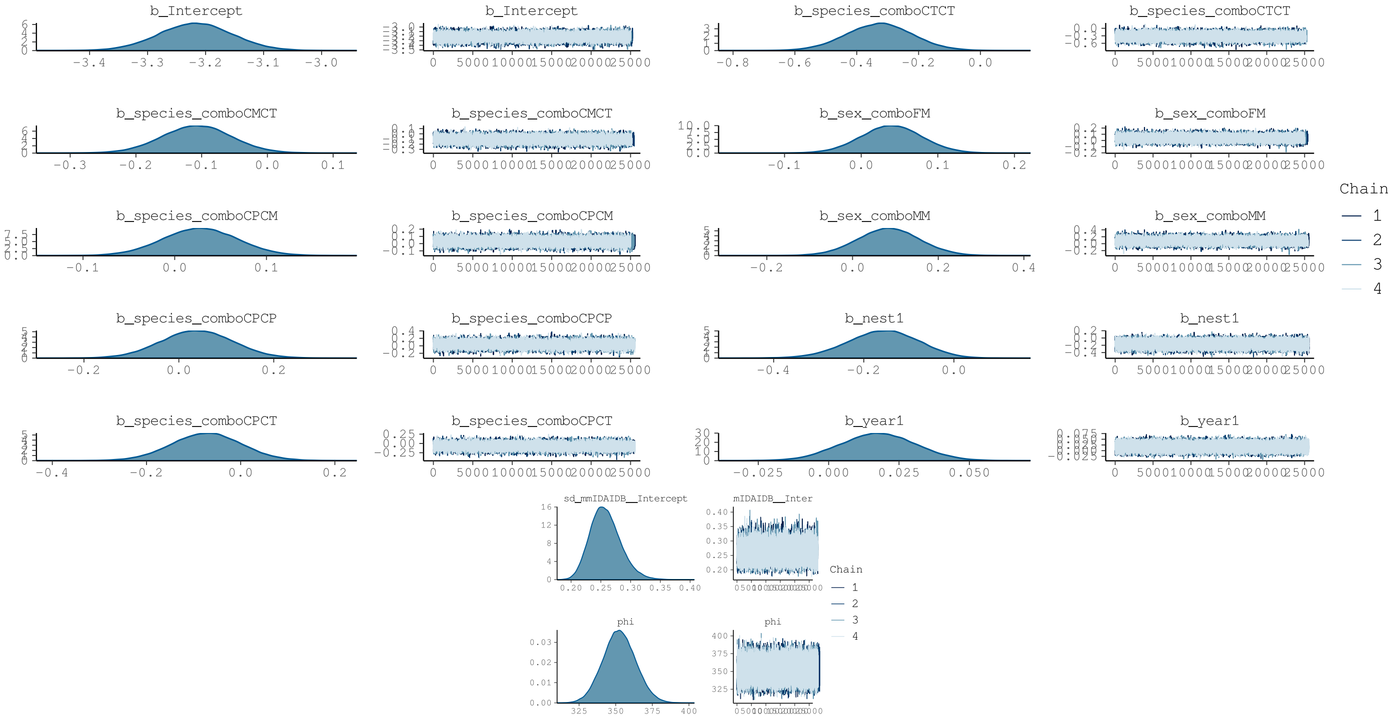

4.4 Plot model results for species comparisson

```
# Bray-Curtis

effect_species <- conditional_effects(model_bc_adults, effects="species_combo") # Default:
doesn't include random effects

plot(effect_species)[[1]] + theme_classic()+
  theme(axis.text.x = element_text(angle = 45, hjust = 1),
        legend.position = "right") +
  theme(axis.text.x = element_text(angle = 45, hjust = 1, size = 14, family = "Arial"),
        axis.text.y = element_text(size = 14, family = "Arial"),
        axis.title.x = element_text(size = 14, family = "Arial"),
        axis.title.y = element_text(size = 14, family = "Arial"),
        legend.position = "right",
        legend.text = element_text(size = 14, family = "Arial"),
        legend.title = element_text(size = 14, family = "Arial"),
        plot.title = element_text(size = 20, family = "Arial"))

# Wheighted UniFrac
effect_species <- conditional_effects(model_wu_adults, effects="species_combo") # Default:
doesn't include random effects

plot(effect_species)[[1]] + theme_classic()+
  theme(axis.text.x = element_text(angle = 45, hjust = 1),
        legend.position = "right") +
  theme(axis.text.x = element_text(angle = 45, hjust = 1, size = 14, family = "Arial"),
        axis.text.y = element_text(size = 14, family = "Arial"),
        axis.title.x = element_text(size = 14, family = "Arial"),
        axis.title.y = element_text(size = 14, family = "Arial"),
        legend.position = "right",
        legend.text = element_text(size = 14, family = "Arial"),
        legend.title = element_text(size = 14, family = "Arial"),
        plot.title = element_text(size = 20, family = "Arial"))
```

4.5. Plot model results for remaining predictors

```
# Bray-Curtis

plot_bc <-mcmc_plot(model_bc_adults, type = "intervals", prob_outer=0.95, prob=0.95,
                  variable =c("b_sex_comboFM", "b_sex_comboMM", "b_nest1","b_year1"))
plot_bc <- plot1 + theme_classic() + geom_vline(xintercept = 0, linetype="dotted",
color="blue")+
  theme(axis.text.x = element_text(size = 16),  # Adjust the size as needed
        axis.text.y = element_text(size = 16))+
  theme(text = element_text(family = "Arial"))
plot_bc


# Wheighted UniFrac
plot_wu <-mcmc_plot(model_wu_adults, type = "intervals", prob_outer=0.95, prob=0.95,
                  variable =c("b_sex_comboFM", "b_sex_comboMM", "b_nest1","b_year1"))

plot_wu <- plot1 + theme_classic() + geom_vline(xintercept = 0, linetype="dotted",
color="blue")+
  theme(axis.text.x = element_text(size = 16),  # Adjust the size as needed
        axis.text.y = element_text(size = 16))+
```

```
  theme(text = element_text(family = "Arial"))
plot_wu
```

5. Bray-Curtis and Weighted UniFrac Models Using brms: Juvenile Individuals

5.1 Model

```
# Model BC dissimilarity

ncores = detectCores()
options(mc.cores = parallel::detectCores())

model_bc_juv <- brm(BC~1+ species_combo + sex_combo + nest + year + (1|mm(IDA,IDB)),
                data = data.dyad_juv,
                family= "zero_one_inflated_beta",
                warmup = 25000, iter = 50000,
                cores = ncores, chains = 4,
                init=0)

saveRDS(model_bc_juv, "model_bc_juv.rds")

> summary(model_bc_juv)

 Family: zero_one_inflated_beta
  Links: mu = logit; phi = identity; zoi = identity; coi = identity
Formula: BC ~ 1 + species_combo + sex_combo + nest + year + (1 | mm(IDA, IDB))
   Data: data.dyad (Number of observations: 2485)
  Draws: 4 chains, each with iter = 50000; warmup = 25000; thin = 1;
         total post-warmup draws = 1e+05

Group-Level Effects:
~mmIDAIDB (Number of levels: 71)
              Estimate Est.Error l-95% CI u-95% CI Rhat Bulk_ESS Tail_ESS
sd(Intercept)     0.58      0.06     0.48     0.70 1.00    16925    27788

Population-Level Effects:
                  Estimate Est.Error l-95% CI u-95% CI Rhat Bulk_ESS Tail_ESS
Intercept             3.85      0.14     3.58     4.12 1.00    12657    23127
species_comboCMCT    -0.07      0.12    -0.30     0.16 1.00    15219    30332
species_comboCPCM     0.05      0.08    -0.11     0.21 1.00    12867    26033
species_comboCPCP    -0.14      0.15    -0.44     0.16 1.00    11750    22000
species_comboCPCT     0.09      0.16    -0.22     0.40 1.00    12723    25325
species_comboCTCT    -0.52      0.23    -0.98    -0.06 1.00    14926    29408
sex_comboFM           0.02      0.08    -0.14     0.17 1.00    12916    25905
sex_comboMM           0.08      0.14    -0.21     0.36 1.00    11663    22120
nest1                -0.36      0.11    -0.56    -0.14 1.00   118520    73174
year1                -0.06      0.03    -0.11    -0.00 1.00   102711    73782

Family Specific Parameters:
    Estimate Est.Error l-95% CI u-95% CI Rhat Bulk_ESS Tail_ESS
phi   122.26      3.70   115.11   129.59 1.00   116062    70794
zoi     0.01      0.00     0.01     0.02 1.00   124040    67418
```

```
coi     0.97      0.03     0.89     1.00 1.00    94871    46331


# Perform hypothesis testing for the remaining species comaprisons

> hypothesis(model_bc_adults, "Intercept + species_comboAPAP = Intercept + species_comboAPAT")
# APAP vs APAT
Hypothesis Tests for class b:
                Hypothesis Estimate Est.Error CI.Lower CI.Upper Evid.Ratio Post.Prob Star
1 (Intercept+specie... = 0    -0.23      0.12    -0.46        0         NA        NA

> hypothesis(model_bc_adults, "Intercept + species_comboAPAP = Intercept + species_comboAPAM")
# APAP vs APAM
   Hypothesis Tests for class b:
                Hypothesis Estimate Est.Error CI.Lower CI.Upper Evid.Ratio Post.Prob Star
1 (Intercept+specie... = 0    -0.19      0.08    -0.35    -0.03         NA        NA    *

> hypothesis(model_bc_adults, "Intercept + species_comboATAT = Intercept + species_comboAPAT")
#  ATAT vs ATAP
Hypothesis Tests for class b:
                Hypothesis Estimate Est.Error CI.Lower CI.Upper Evid.Ratio Post.Prob Star
1 (Intercept+specie... = 0    -0.61      0.14    -0.88    -0.34         NA        NA    *

> hypothesis(model_bc_adults, "Intercept + species_comboATAT = Intercept + species_comboAMAT")
#  ATAT vs ATAM
Hypothesis Tests for class b:
                Hypothesis Estimate Est.Error CI.Lower CI.Upper Evid.Ratio Post.Prob Star
1 (Intercept+specie... = 0    -0.45      0.14    -0.72    -0.18         NA        NA    *

> hypothesis(model_bc_adults, "Intercept + species_comboATAT = Intercept + species_comboAPAP")
#  ATAT vs APAP
Hypothesis Tests for class b:
                Hypothesis Estimate Est.Error CI.Lower CI.Upper Evid.Ratio Post.Prob Star
1 (Intercept+specie... = 0    -0.38      0.23    -0.84     0.08         NA        NA



# Model Wheighted-UniFrac distances
ncores = detectCores()
options(mc.cores = parallel::detectCores())

model_wu_juv <- brm(WU~1+ species_combo + + sex_combo + nest + year + (1|mm(IDA,IDB)),
                data = data.dyad_juv,
                family= "Beta",
                warmup = 25000, iter = 50000,
                cores = ncores, chains = 4,
                init=0)

saveRDS(model_wu_juv, "model_wu_juv.rds")

> summary(model_wu_juv)

 Family: beta
  Links: mu = logit; phi = identity
Formula: WU ~ 1 + species_combo + +sex_combo + nest + year + (1 | mm(IDA, IDB))
   Data: data.dyad (Number of observations: 2485)
  Draws: 4 chains, each with iter = 50000; warmup = 25000; thin = 1;
         total post-warmup draws = 1e+05

Group-Level Effects:
```

```
~mmIDAIDB (Number of levels: 71)
              Estimate Est.Error l-95% CI u-95% CI Rhat Bulk_ESS Tail_ESS
sd(Intercept)     0.24      0.02     0.20     0.29 1.00    17303    26969

Population-Level Effects:
                  Estimate Est.Error l-95% CI u-95% CI Rhat Bulk_ESS Tail_ESS
Intercept            -3.14      0.06    -3.26    -3.03 1.00    12699    24230
species_comboCMCT     0.09      0.05    -0.01     0.19 1.00    16002    28635
species_comboCPCM     0.01      0.03    -0.06     0.08 1.00    12692    26109
species_comboCPCP    -0.01      0.06    -0.14     0.11 1.00    11432    21829
species_comboCPCT     0.17      0.07     0.04     0.30 1.00    12161    23809
species_comboCTCT    -0.18      0.11    -0.40     0.03 1.00    17179    32857
sex_comboFM           0.00      0.03    -0.07     0.07 1.00    14509    27307
sex_comboMM          -0.02      0.06    -0.14     0.10 1.00    12694    23426
nest1                 0.05      0.07    -0.09     0.17 1.00    94018    69910
year1                 0.00      0.01    -0.03     0.03 1.00    74578    71076

Family Specific Parameters:
    Estimate Est.Error l-95% CI u-95% CI Rhat Bulk_ESS Tail_ESS
phi   285.65      8.22   269.72   301.90 1.00    90704    71671


# Perform hypothesis testing for the remaining species comaprisons

> hypothesis(model_wu_adults, "Intercept + species_comboAPAP = Intercept + species_comboAPAT")
# APAP vs APAT
Hypothesis Tests for class b:
                Hypothesis Estimate Est.Error CI.Lower CI.Upper Evid.Ratio Post.Prob Star
1 (Intercept+specie... = 0    -0.18      0.05    -0.28    -0.08         NA        NA    *

> hypothesis(model_wu_adults, "Intercept + species_comboAPAP = Intercept + species_comboAPAM")
# APAP vs APAM
Hypothesis Tests for class b:
                Hypothesis Estimate Est.Error CI.Lower CI.Upper Evid.Ratio Post.Prob Star
1 (Intercept+specie... = 0    -0.02      0.03    -0.09     0.05         NA        NA

> hypothesis(model_wu_adults, "Intercept + species_comboATAT = Intercept + species_comboAPAT")
#  ATAT vs ATAP
Hypothesis Tests for class b:
                Hypothesis Estimate Est.Error CI.Lower CI.Upper Evid.Ratio Post.Prob Star
1 (Intercept+specie... = 0    -0.35      0.07     -0.5    -0.21         NA        NA    *

> hypothesis(model_wu_adults, "Intercept + species_comboATAT = Intercept + species_comboAMAT")
#  ATAT vs ATAM
Hypothesis Tests for class b:
                Hypothesis Estimate Est.Error CI.Lower CI.Upper Evid.Ratio Post.Prob Star
1 (Intercept+specie... = 0    -0.28      0.07    -0.42    -0.13         NA        NA    *

> hypothesis(model_wu_adults, "Intercept + species_comboATAT = Intercept + species_comboAPAP")
#  ATAT vs APAP
  Hypothesis Tests for class b:
                Hypothesis Estimate Est.Error CI.Lower CI.Upper Evid.Ratio Post.Prob Star
1 (Intercept+specie... = 0    -0.17      0.11    -0.38     0.04         NA        NA
```

5.2 Compare distribution of response variable to distributions of predicted response variable values

```
pp_check(model_bc_juv, ndraws = 100)

pp_check(model_wu_juv, ndraws = 100)
```

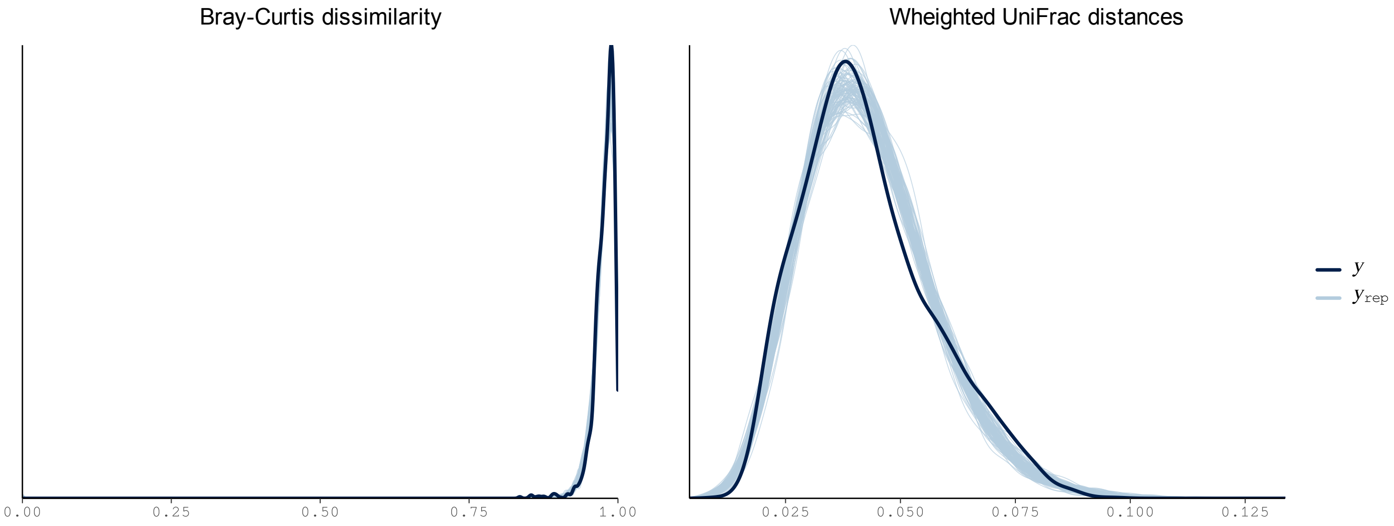

5.3 Model diagnostics

```
# BC model diagnostics
plot(model_bc_juv)
```

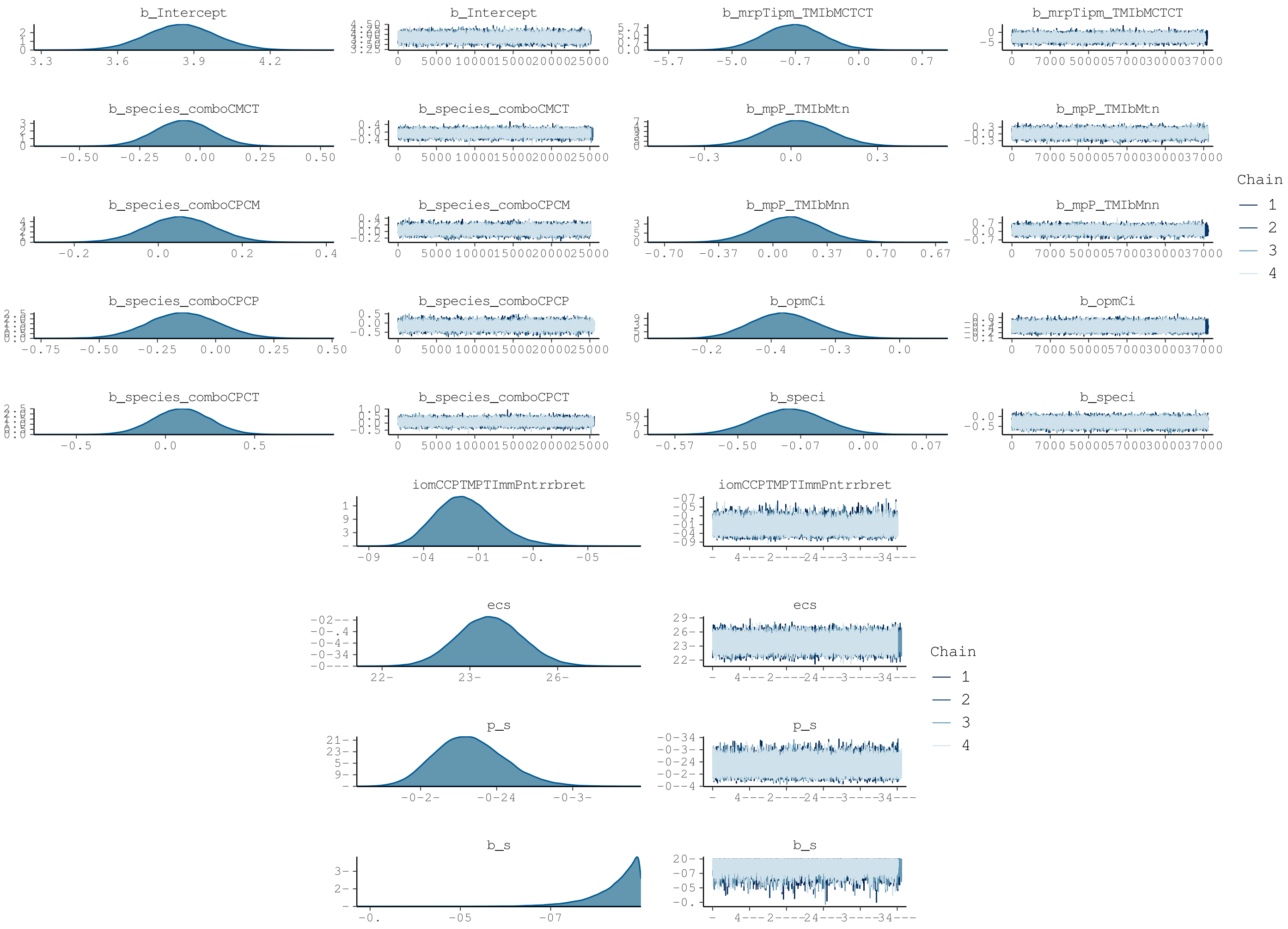

```
# WU model diagnostics
plot(model_wu_juv)
```

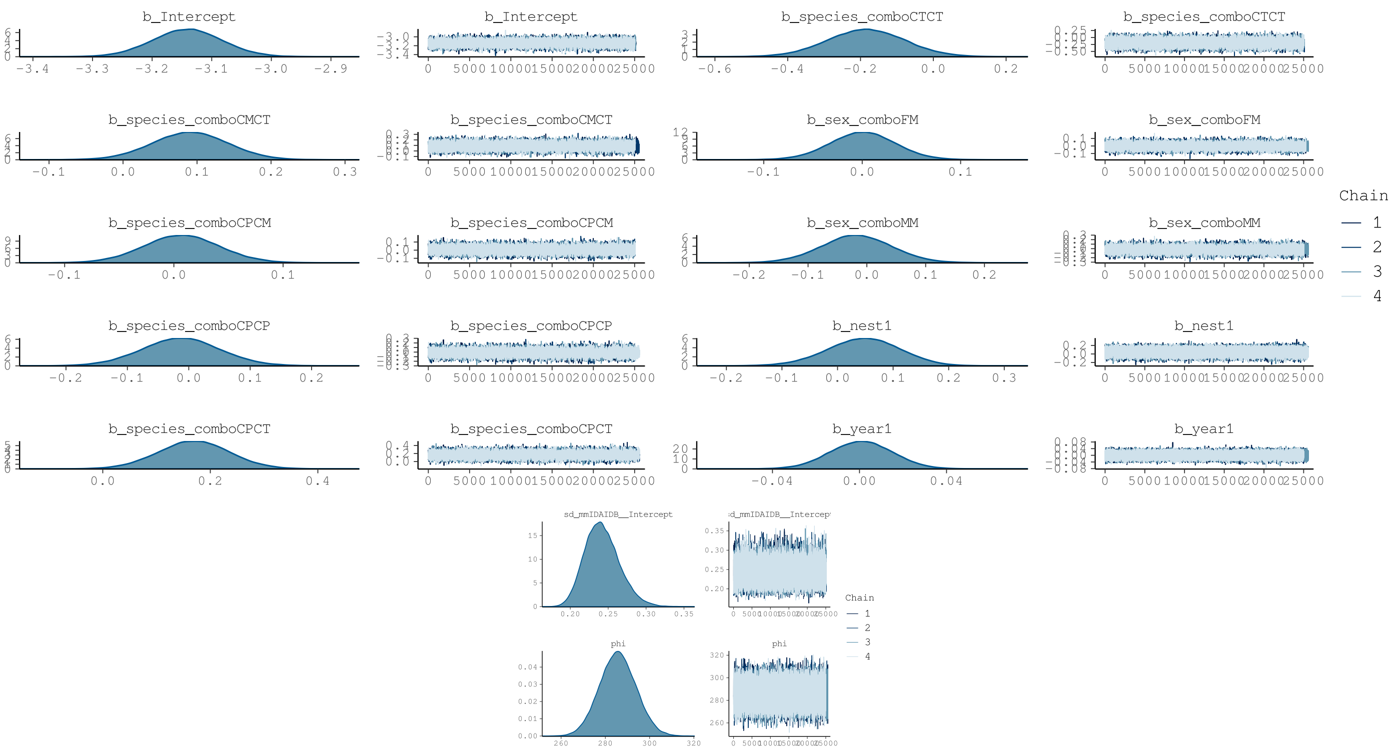

5.4 Plot model results for species comparison

```
# Bray-Curtis

effect_species <- conditional_effects(model_bc_juv, effects="species_combo") # Default: doesn't
include random effects

plot(effect_species)[[1]] + theme_classic()+
  theme(axis.text.x = element_text(angle = 45, hjust = 1),
        legend.position = "right") +
  theme(axis.text.x = element_text(angle = 45, hjust = 1, size = 14, family = "Arial"),
        axis.text.y = element_text(size = 14, family = "Arial"),
        axis.title.x = element_text(size = 14, family = "Arial"),
        axis.title.y = element_text(size = 14, family = "Arial"),
        legend.position = "right",
        legend.text = element_text(size = 14, family = "Arial"),
        legend.title = element_text(size = 14, family = "Arial"),
        plot.title = element_text(size = 20, family = "Arial"))

# Wheighted UniFrac
effect_species <- conditional_effects(model_wu_juv, effects="species_combo") # Default: doesn't
include random effects

plot(effect_species)[[1]] + theme_classic()+
  theme(axis.text.x = element_text(angle = 45, hjust = 1),
        legend.position = "right") +
  theme(axis.text.x = element_text(angle = 45, hjust = 1, size = 14, family = "Arial"),
        axis.text.y = element_text(size = 14, family = "Arial"),
        axis.title.x = element_text(size = 14, family = "Arial"),
        axis.title.y = element_text(size = 14, family = "Arial"),
        legend.position = "right",
        legend.text = element_text(size = 14, family = "Arial"),
        legend.title = element_text(size = 14, family = "Arial"),
        plot.title = element_text(size = 20, family = "Arial"))
```

5.5. Plot model results for remaining predictors

```
# Bray-Curtis

plot_bc_juv <-mcmc_plot(model_bc_juv, type = "intervals", prob_outer=0.95, prob=0.95,
                  variable =c("b_sex_comboFM", "b_sex_comboMM", "b_nest1","b_year1"))
plot_bc_juv <- plot1 + theme_classic() + geom_vline(xintercept = 0, linetype="dotted",
color="blue")+
  theme(axis.text.x = element_text(size = 16),  # Adjust the size as needed
        axis.text.y = element_text(size = 16))+
  theme(text = element_text(family = "Arial"))
plot_bc_juv


# Wheighted UniFrac
plot_wu_juv <-mcmc_plot(model_wu_juv, type = "intervals", prob_outer=0.95, prob=0.95,
                  variable =c("b_sex_comboFM", "b_sex_comboMM", "b_nest1","b_year1"))

plot_wu_juv <- plot1 + theme_classic() + geom_vline(xintercept = 0, linetype="dotted",
color="blue")+
  theme(axis.text.x = element_text(size = 16),  # Adjust the size as needed
        axis.text.y = element_text(size = 16))+
  theme(text = element_text(family = "Arial"))
plot_wu_juv
```

C) Spacial distance and compositional differences

```
# Load required packages

install.packages(c("sp", "rgdal"))
library(sp)
library(rgdal)

#read ps object
ps_css <- readRDS("ps_css.rds")

# remove individuals without gps information
individuals_to_prune <- c("FJ02235", "FJ02223", "FH80666", "FH69257")
ps_css <- prune_samples(!(sample_data(ps_css)$ring_number %in% individuals_to_prune), ps_css)

metadata <- sample_data(ps_css)

#Make Bray curtis matrix from microbiome data using vegdist function embedded in
phyloseq::distance
BCM<- as.matrix(phyloseq::distance(ps_css, method = "bray", type = "samples"))

#Make weighted unifrac matrix
WUM <- as.matrix(phyloseq::distance(ps_css, method = "wunifrac", type = "samples"))

# Make distance matrix

## Create a dataframe with dsitance data
```

```
distance_df <- metadata[, c("identifier", "utm_1", "utm_2")]
#distance_df <- na.omit(distance_df) #remove NA

## Create a Spatial Points DataFrame
coordinates <- distance_df[, c("utm_1", "utm_2")]
proj4string <- CRS("+proj=utm +zone=38K +datum=WGS84") # coordinate reference system
spdf <- SpatialPointsDataFrame(coordinates, data = distance_df, proj4string = proj4string)

# Calculate distances
distance_matrix <- spDists(spdf)
key<-data.frame(identifier=distance_df$identifier)# assigne proper names to the matrix
rownames(distance_matrix)<-key$identifier # assign ring_number to rows
colnames(distance_matrix)<-key$identifier#assign ring_number to columns

# Mantel test between BC WU and distance between individuals
distance_m<- as.dist(distance_matrix)
BC_d <- as.dist(BCM)
WU_d <- as.dist(WUM)

> mantel(BC_d,distance_m,permutations = 9999, strata = metadata$nest)

Mantel statistic based on Pearsons product-moment correlation

Call:
mantel(xdis = BC_d, ydis = distance_m, permutations = 9999, strata = metadata$nest)

Mantel statistic r: 0.005618
      Significance: 0.7083

Upper quantiles of permutations (null model):
   90%    95%  97.5%    99%
0.0195 0.0221 0.0241 0.0266
Blocks:  strata
Permutation: free
Number of permutations: 9999


> mantel(WU_d,distance_m,permutations = 9999, strata = metadata$nest)

Mantel statistic based on Pearsons product-moment correlation

Call:
mantel(xdis = WU_d, ydis = distance_m, permutations = 9999, strata = metadata$nest)

Mantel statistic r: -0.02702
      Significance: 0.8927

Upper quantiles of permutations (null model):
     90%      95%    97.5%      99%
-0.01118 -0.00918 -0.00752 -0.00570
Blocks:  strata
Permutation: free
Number of permutations: 9999
```
