# Supplementary material for: The gut microbiota of three avian species living in sympatry
Source: BMC Ecol Evol. 2024 Nov 21;24:144. doi: 10.1186/s12862-024-02329-9 (PMC11580620; doi:10.1186/s12862-024-02329-9)
Supplement: Supplementary file 5 — Additional file 5. Appendix E. Alpha diversity statistical analysis. [file 12862_2024_2329_MOESM5_ESM.pdf]

Alpha diversity statistical analysis

Table of Contents

Alpha diversity statistical analysis
- Table of Contents
- Read in the data

A) Alpha diversity - adults and juveniles
- 1. Model shannon diversity index
  - 1.1 Check Normality
  - 1.2 Model Diagnostics
  - 1.3 Model Summary
  - 1.4 Bootstrap model
  - 1.5 Marginal and conditional R-squared
  - 1.6 Plot model effects
  - 1.7 Significance of random effects
- 2. Model Faith phylogenetic diversity
  - 2.1 Check Normality
  - 2.2 Model Diagnostics
  - 2.3 Model Summary
  - 2.4 Bootstrap model
  - 2.5 Marginal and conditional R-squared
  - 2.6 Plot model effects
  - 2.7 Significance of random effects
- 3. Model n° of observed ASV's
  - 3.1 Check Normality
  - 3.2 Model Diagnostics
  - 3.3 Model Summary
  - 3.4 Bootstrap model
  - 3.5 Marginal and conditional R-squared
  - 3.6 Plot model effects
  - 3.7 Significance of random effects

B) Alpha diversity - adults
- 1. Model shannon diversity index
  - 1.1 Check Normality
  - 1.2 Model Diagnostics
  - 1.3 Model Summary
  - 1.4 Bootstrap model
  - 1.5 Marginal and conditional R-squared
  - 1.6 Plot model effects
  - 1.7 Significance of random effects
- 2. Model Faith phylogenetic diversity
  - 2.1 Check Normality
  - 2.2 Model Diagnostics
  - 2.3 Model Summary
  - 2.4 Bootstrap model
  - 2.5 Marginal and conditional R-squared
  - 2.6 Plot model effects
  - 2.7 Significance of random effects
- 3. Model n° of observed ASV's
  - 3.1 Check Normality
  - 3.2 Model Diagnostics
  - 3.3 Model Summary
  - 3.4 Bootstrap model

3.5 Marginal and conditional R-squared
3.6 Plot model effects
3.7 Significance of random effects
C) Alpha diversity - juveniles
1. Model shannon diversity index
1.1 Check Normality
1.2 Model Diagnostics
1.3 Model Summary
1.4 Bootstrap model
1.5 Marginal and conditional R-squared
1.6 Plot model effects
1.7 Significance of random effects
2. Model Faith phylogenetic diversity
2.1 Check Normality
2.2 Model Diagnostics
2.3 Model Summary
2.4 Bootstrap model
2.5 Marginal and conditional R-squared
2.6 Plot model effects
2.7 Significance of random effects
3. Model n° of observed ASV's
3.1 Check Normality
3.2 Model Diagnostics
3.3 Model Summary
3.4 Bootstrap model
3.5 Marginal and conditional R-squared
3.6 Plot model effects
3.7 Significance of random effects

Read in the data

```
# Load libraries

library(lubridate)
library(tidyverse)
library(lme4)
library(MuMIn)
library (performance)
library(datawizard)
library(car)
library(effects)
library(openxlsx)
library(multcomp)
library(grafify)
library(correlation)
library(lmerTest)
library(lmeresampler)

# Read in the data
metadata <- read.table("plover_metadata.tsv"  , header = TRUE, sep = "\t", stringsAsFactors =
FALSE)
metadata <- metadata[, -1] # This removes the first column
```

```
# Find duplicates
duplicates <- duplicated(metadata$ring_number)

# Keep only the first occurrence of each duplicate
metadata <- metadata[!duplicates, ]

# Keep only the first occurrence of each duplicate
metadata <- metadata[!duplicates, ]
#remoce individuals with no sex assignement
metadata <- subset(metadata, sex != "")
#remove individuals from nests with only one individual
non_unique_nest <- metadata$nest[duplicated(metadata$nest)]
metadata <- metadata[metadata$nest %in% non_unique_nest, ]

metadata$ring_number <- as.factor(metadata$ring_number)
metadata$nest <- as.factor(metadata$nest)
metadata$year <- as.factor(metadata$year)
metadata$species <- as.factor(metadata$species)
metadata$sex <- as.factor(metadata$sex)
metadata$age <- as.factor(metadata$age)

metadata$shannon_entropy <- as.numeric(metadata$shannon_entropy)
metadata$std_shannon <- scale(metadata$shannon_entropy)
metadata$std_shannon <- as.numeric(metadata$std_shannon)

metadata$observed_features <- as.numeric(metadata$observed_features)
metadata$std_asv <- scale(metadata$observed_features)
metadata$std_asv <- as.numeric(metadata$std_asv)

metadata$faith_pd <- as.numeric(metadata$faith_pd)
metadata$std_faith <- scale(metadata$faith_pd)
metadata$std_faith <- as.numeric(metadata$std_faith)

saveRDS(metadata, "metadata.rds")
```

A) Alpha diversity - adults and juveniles

1. Model shannon diversity index

```
# Plot data distribution
hist(metadata$std_shannon)

# Check for outliers
> check_outliers(metadata$std_shannon)
OK: No outliers detected.
- Based on the following method and threshold: zscore_robust (3.291).
- For variable: metadata$std_shannon

model_shannon <- lmer(std_shannon ~ species + age + sex + year + (1|nest),data = metadata)
```

1.1 Check Normality

```
> check_normality(model_shannon)
Warning: Non-normality of residuals detected (p = 0.005).
```

1.2 Model Diagnostics

```
> plot(check_distribution(model_shannon))

# Extract residuals
residuals_shannon <- resid(model_shannon)
# Create QQ plot
qqPlot(residuals_shannon, envelope = 0.95, main = "Std. Shannon QQ plot")
```

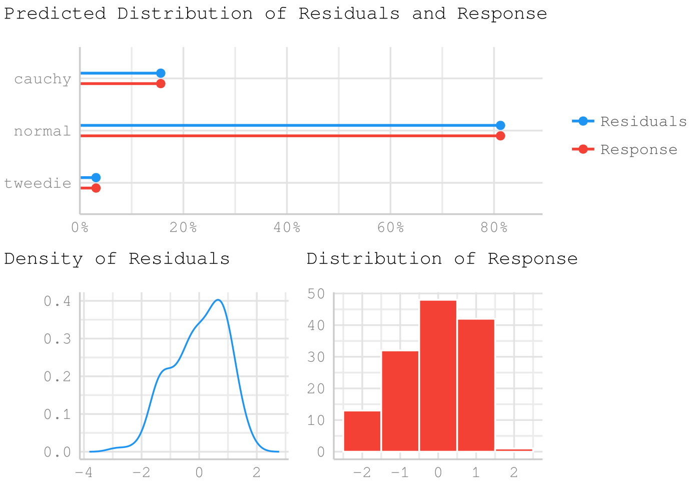

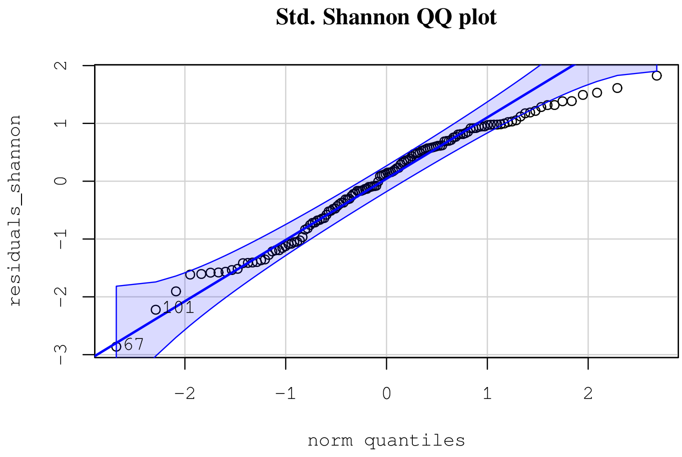

1.3 Model Summary

```
> summary(model_shannon1)
Linear mixed model fit by REML. t-tests use Satterthwaites method ['lmerModLmerTest']
Formula: std_shannon ~ species + sex + age + year + (1 | nest)
   Data: metadata

REML criterion at convergence: 379.4

Scaled residuals:
    Min      1Q  Median      3Q     Max
-2.9845 -0.7002  0.1425  0.7907  1.9038

Random effects:
 Groups   Name        Variance Std.Dev.
 nest     (Intercept) 0.0000   0.0000
 Residual             0.9199   0.9591
Number of obs: 136, groups:  nest, 55

Fixed effects:
                    Estimate Std. Error        df t value Pr(>|t|)
(Intercept)         -0.03034    0.20902 130.00000  -0.145   0.8848
speciesApecuarius    0.38855    0.18482 130.00000   2.102   0.0375 *
speciesAthoracicus  -0.57568    0.25871 130.00000  -2.225   0.0278 *
sexM                 0.02584    0.17282 130.00000   0.150   0.8814
ageJ                -0.18973    0.16535 130.00000  -1.147   0.2533
year2022             0.06620    0.18850 130.00000   0.351   0.7260
---
```

```
Signif. codes:  0 '***' 0.001 '**' 0.01 '*' 0.05 '.' 0.1 ' ' 1
```

1.4 Bootstrap model

```
> boot_model_shannon <- bootstrap(model_shannon,.f = fixef, type = "parametric", B = 10000,
resample = c(TRUE, TRUE))

> summary(boot_model_shannon)
Bootstrap type: parametric

Number of resamples: 10000

                term    observed    rep.mean        se          bias
1        (Intercept) -0.03034127 -0.03330349 0.2096602 -2.962212e-03
2  speciesApecuarius  0.38855457  0.38864123 0.1857920  8.666335e-05
3 speciesAthoracicus -0.57567724 -0.57564737 0.2590437  2.986659e-05
4               sexM  0.02584008  0.02777821 0.1719586  1.938138e-03
5               ageJ -0.18972746 -0.19007339 0.1661201 -3.459322e-04
6           year2022  0.06620038  0.06742600 0.1898363  1.225623e-03

> confint(boot_model_shannon, type = "norm")
# A tibble: 6 × 6
  term               estimate   lower   upper type  level
  <chr>                 <dbl>   <dbl>   <dbl> <chr> <dbl>
1 (Intercept)         -0.0303 -0.438   0.384  norm   0.95
2 speciesApecuarius    0.389   0.0243  0.753  norm   0.95
3 speciesAthoracicus  -0.576  -1.08   -0.0680 norm   0.95
4 sexM                 0.0258 -0.313   0.361  norm   0.95
5 ageJ                -0.190  -0.515   0.136  norm   0.95
6 year2022             0.0662 -0.307   0.437  norm   0.95

# Note: In order to compute the confidence intervals for the remaining comparison (A.
thoracicus vs. A. pecuarius)change the reference level of the metadata to Athoracicus and
repeat the analysis.
metadata$species <- relevel(metadata2$species, ref = "Athoracicus")

> confint(boot_model_shannon, type = "norm") # with Athoracicus as reference level
# A tibble: 6 × 6
  term               estimate   lower   upper type  level
  <chr>                 <dbl>   <dbl>   <dbl> <chr> <dbl>
1 (Intercept)         -0.606  -1.12   -0.0929 norm   0.95
2 speciesAmarginatus   0.576   0.0674  1.09   norm   0.95
3 speciesApecuarius    0.964   0.470   1.46   norm   0.95
4 sexM                 0.0258 -0.312   0.364  norm   0.95
5 ageJ                -0.190  -0.513   0.132  norm   0.95
6 year2022             0.0662 -0.305   0.441  norm   0.95
```

1.5 Marginal and conditional R-squared

```
> r.squaredGLMM(model_shannon)
           R2m       R2c
[1,] 0.1103745 0.1103745
```

1.6 Plot model effects

```
> plot(allEffects(model_shannon))
```

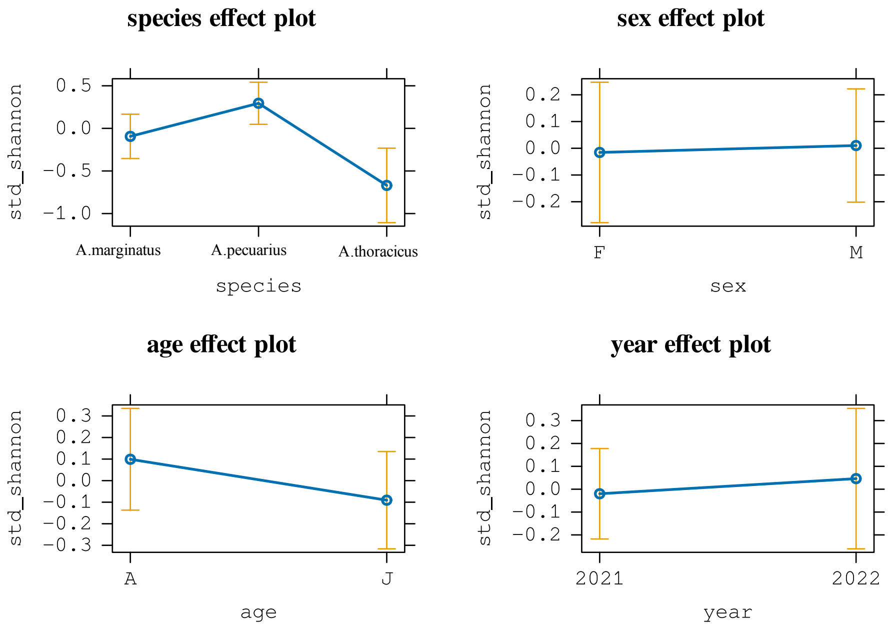

1.7 Significance of random effects

```
> ranova(model_shannon)
ANOVA-like table for random-effects: Single term deletions

Model:
std_shannon ~ species + sex + age + year + (1 | nest)
           npar  logLik    AIC LRT Df Pr(>Chisq)
<none>        8 -189.69 395.39
(1 | nest)    7 -189.69 393.39   0  1          1
```

2. Model Faith phylogenetic diversity

```
# Plot data distribution
hist(metadata$std_faith)

# Check for outliers
check_outliers(metadata$std_faith)
OK: No outliers detected.
- Based on the following method and threshold: zscore_robust (3.291).
- For variable: metadata$std_faith

# Model faith diversity
model_faith <- lmer(std_faith ~ species + age + sex + year + (1|nest),data = metadata)
> check_normality(model_faith1)
Warning: Non-normality of residuals detected (p = 0.035).

#Transform faith pd
metadata$sqrt_faith <- sqrt(metadata_$faith_pd)
```

```
# Model transformed faith diversity
model_faith <- lmer(sqrt_faith ~ species + sex + year + (1|nest),data = metadata_juv)
```

2.1 Check Normality

```
> check_normality(model_faith)
Warning: Non-normality of residuals detected (p = 0.035).
```

2.2 Model Diagnostics

```
> plot(check_distribution(model_faith))

# Extract residuals
residuals_faith <- resid(model_faith)
# Create QQ plot
qqPlot(residuals_faith, envelope = 0.95, main = "Sqrt Faith PD QQ plot")
```

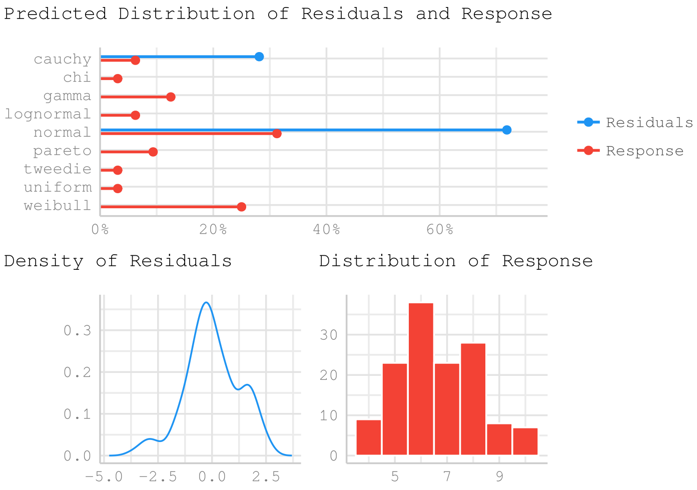

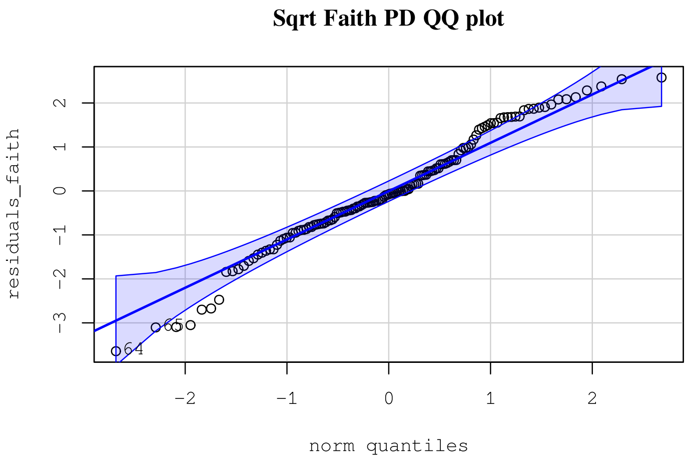

2.3 Model Summary

```
> summary(model_faith)
Linear mixed model fit by REML. t-tests use Satterthwaites method ['lmerModLmerTest']
Formula: sqrt_faith ~ species + sex + age + year + (1 | nest)
   Data: metadata

REML criterion at convergence: 471.1

Scaled residuals:
     Min       1Q   Median       3Q      Max
-2.75203 -0.56312 -0.05763  0.55627  1.94754

Random effects:
 Groups   Name        Variance Std.Dev.
 nest     (Intercept) 0.1208   0.3475
 Residual             1.7532   1.3241
Number of obs: 136, groups:  nest, 55

Fixed effects:
                   Estimate Std. Error       df t value Pr(>|t|)
(Intercept)          6.0099     0.3065  77.0815  19.608  < 2e-16 ***
```

```
speciesApecuarius    1.2851     0.2782  39.0447   4.620 4.11e-05 ***
speciesAthoracicus  -0.3103     0.3883  41.4713  -0.799    0.429
sexM                 0.1761     0.2445 125.4179   0.720    0.473
ageJ                -0.1549     0.2303  95.1482  -0.673    0.503
year2022             0.2778     0.2833  40.2536   0.981    0.333
---
Signif. codes:  0 '***' 0.001 '**' 0.01 '*' 0.05 '.' 0.1 ' ' 1
```

2.4 Bootstrap model

```
> boot_model_faith <- bootstrap(model_faith,.f = fixef, type = "parametric", B = 10000,
resample = c(TRUE, TRUE))

> summary(boot_model_faith)
Bootstrap type: parametric

Number of resamples: 10000

                term   observed   rep.mean        se          bias
1        (Intercept)  6.0099267  6.0062443 0.3069361 -0.0036824007
2  speciesApecuarius  1.2851115  1.2904791 0.2802872  0.0053676080
3 speciesAthoracicus -0.3103395 -0.3096763 0.3904975  0.0006631797
4               sexM  0.1761289  0.1754492 0.2464282 -0.0006796500
5               ageJ -0.1549177 -0.1552249 0.2296728 -0.0003071454
6           year2022  0.2778294  0.2803281 0.2822674  0.0024987097

> confint(boot_model_faith, type = "norm")
# A tibble: 6 × 6
  term                estimate  lower upper type  level
  <chr>                  <dbl>  <dbl> <dbl> <chr> <dbl>
1 (Intercept)            6.01   5.41  6.62  norm   0.95
2 speciesApecuarius      1.29   0.730 1.83  norm   0.95
3 speciesAthoracicus    -0.310 -1.08  0.454 norm   0.95
4 sexM                   0.176 -0.306 0.660 norm   0.95
5 ageJ                  -0.155 -0.605 0.296 norm   0.95
6 year2022               0.278 -0.278 0.829 norm   0.95

# Note: In order to compute the confidence intervals for the remaining comparison (A.
thoracicus vs. A. pecuarius)change the reference level of the metadata to Athoracicus and
repeat the analysis.
metadata$species <- relevel(metadata2$species, ref = "Athoracicus")

> confint(boot_model_faith, type = "norm") # with Athoracicus as reference level
# A tibble: 6 × 6
  term                estimate  lower upper type  level
  <chr>                  <dbl>  <dbl> <dbl> <chr> <dbl>
1 (Intercept)            5.70   4.94  6.47  norm   0.95
2 speciesAmarginatus     0.310 -0.457 1.07  norm   0.95
3 speciesApecuarius      1.60   0.847 2.35  norm   0.95
4 sexM                   0.176 -0.309 0.654 norm   0.95
5 ageJ                  -0.155 -0.613 0.298 norm   0.95
6 year2022               0.278 -0.274 0.831 norm   0.95
```

2.5 Marginal and conditional R-squared

```
> r.squaredGLMM(model_faith)
           R2m       R2c
[1,] 0.2059659 0.2571445
```

2.6 Plot model effects

```
> plot(allEffects(model_faith))
```

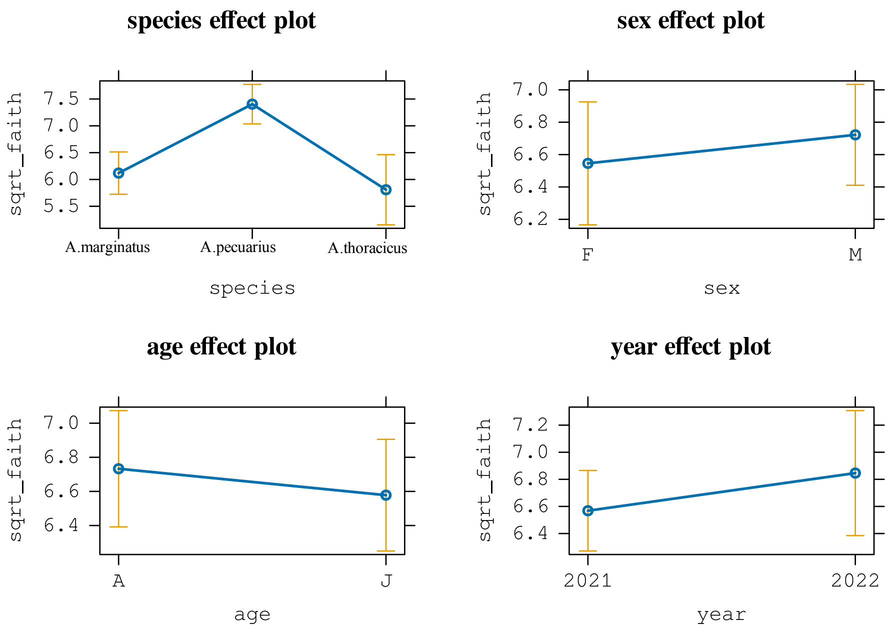

2.7 Significance of random effects

```
> ranova(model_faith)
ANOVA-like table for random-effects: Single term deletions

Model:
sqrt_faith ~ species + sex + age + year + (1 | nest)
           npar  logLik    AIC     LRT Df Pr(>Chisq)
<none>        8 -235.54 487.08
(1 | nest)    7 -235.71 485.42 0.34169  1     0.5589
```

3. Model n° of observed ASV's

```
# Plot data distribution
hist(metadata$std_asv)

# Check for outliers
> check_outliers(metadata$std_asv)
OK: No outliers detected.
- Based on the following method and threshold: zscore_robust (3.291).
- For variable: metadata$std_asv
```

```
# Model n° of aboserved asv's
model_asv <- lmer(std_asv ~ species + age +  sex + year + (1|nest),data = metadata)

# Check normality
> check_normality(model_asv)

#Log transform rsponse variable
metadata$log_asv <- log10(metadata$observed_features)
hist(metadata$log_asv)
check_distribution(metadata$log_asv)

#Model transformed response variable
model_asv <- lmer(log_asv ~ species + age + sex + year + (1|nest),data = metadata)
```

3.1 Check Normality

```
> check_normality(model_asv)
Warning: Non-normality of residuals detected (p = 0.001).
```

3.2 Model Diagnostics

```
plot(check_distribution(model_asv))

# Extract residuals
residuals_asv <- resid(model_asv1)
# Create QQ plot
qqPlot(residuals_asv, envelope = 0.95, main = "Log ASV QQ plot")
```

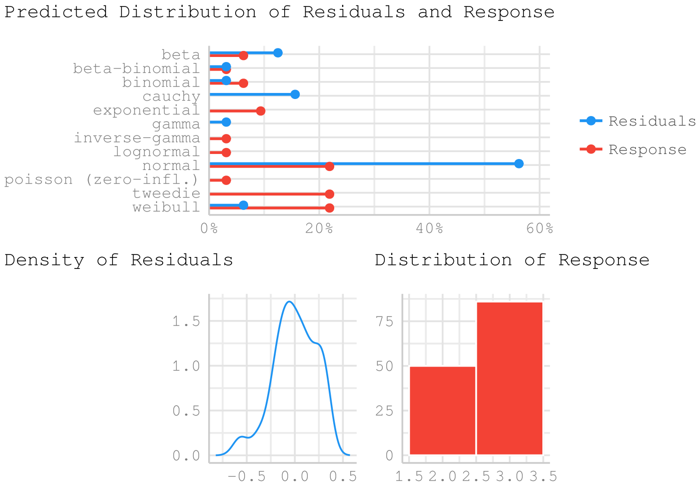

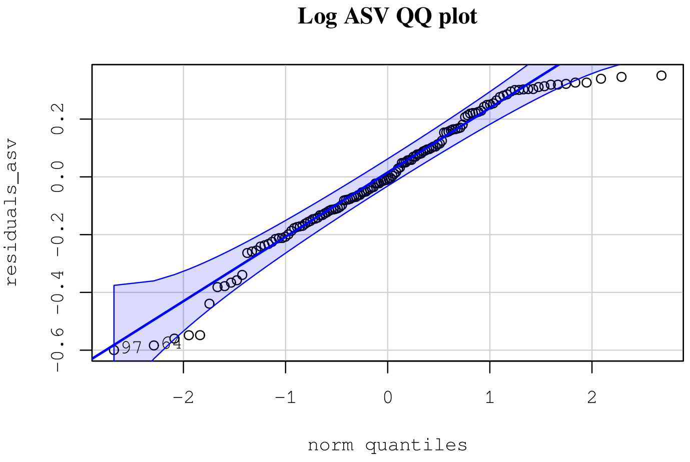

3.3 Model Summary

```
> summary(model_asv)
Linear mixed model fit by REML. t-tests use Satterthwaites method ['lmerModLmerTest']
Formula: log_asv ~ species + sex + age + year + (1 | nest)
   Data: metadata

REML criterion at convergence: 9.7

Scaled residuals:
```

```
     Min       1Q   Median       3Q      Max 
-2.63347 -0.59267 -0.03676  0.72797  1.54074 

Random effects:
 Groups   Name        Variance Std.Dev.
 nest     (Intercept) 0.001851 0.04303 
 Residual             0.051841 0.22769 
Number of obs: 136, groups:  nest, 55

Fixed effects:
                    Estimate Std. Error        df t value Pr(>|t|)    
(Intercept)          2.48965    0.05127  76.66176  48.563  < 2e-16 ***
speciesApecuarius    0.21000    0.04598  38.11750   4.567 5.06e-05 ***
speciesAthoracicus  -0.02443    0.06427  40.72999  -0.380    0.706    
sexM                 0.02054    0.04159 126.81443   0.494    0.622    
ageJ                -0.02182    0.03945  96.61883  -0.553    0.581    
year2022             0.07048    0.04687  39.30007   1.504    0.141    
---
Signif. codes:  0 '***' 0.001 '**' 0.01 '*' 0.05 '.' 0.1 ' ' 1
```

3.4 Bootstrap model

```
> boot_model_asv <- bootstrap(model_asv,.f = fixef, type = "parametric", B = 10000, resample =
c(TRUE, TRUE))

> summary(boot_model_asv1)
ootstrap type: parametric

Number of resamples: 10000

                term    observed    rep.mean          se          bias
1        (Intercept)  2.48964926  2.48840729 0.05076993 -1.241974e-03
2  speciesCpecuarius  0.20999986  0.20999610 0.04593271 -3.764616e-06
3 speciesCthoracicus -0.02442608 -0.02333597 0.06416642  1.090110e-03
4               sexM  0.02053911  0.02114403 0.04137600  6.049175e-04
5               ageJ -0.02182044 -0.02146038 0.03972102  3.600592e-04
6           year2022  0.07048407  0.07119559 0.04715458  7.115245e-04

> confint(boot_model_asv, type = "norm")
# A tibble: 6 × 6
  term                estimate   lower  upper type  level
  <chr>                  <dbl>   <dbl>  <dbl> <chr> <dbl>
1 (Intercept)           2.49     2.39   2.59  norm   0.95
2 speciesApecuarius     0.210    0.120  0.300 norm   0.95
3 speciesAthoracicus   -0.0244 -0.151  0.100  norm   0.95
4 sexM                  0.0205 -0.0612 0.101  norm   0.95
5 ageJ                 -0.0218 -0.100  0.0557 norm   0.95
6 year2022              0.0705 -0.0226 0.162  norm   0.95


# Note: In order to compute the confidence intervals for the remaining comparison (A.
thoracicus vs. A. pecuarius)change the reference level of the metadata to Athoracicus and
repeat the analysis.
metadata$species <- relevel(metadata$species, ref = "Athoracicus")

> confint(boot_model_asv, type = "norm") # with Athoracicus as reference level
# A tibble: 6 × 6
```

```
  term                 estimate   lower   upper type  level
  <chr>                   <dbl>   <dbl>   <dbl> <chr> <dbl>
1 (Intercept)            2.47     2.34    2.59  norm   0.95
2 speciesAmarginatus     0.0244 -0.102  0.150  norm   0.95
3 speciesApecuarius      0.234    0.111   0.357  norm   0.95
4 sexM                   0.0205 -0.0614 0.103  norm   0.95
5 ageJ                  -0.0218 -0.0988 0.0547 norm   0.95
6 year2022               0.0705 -0.0209 0.164  norm   0.95
```

3.5 Marginal and conditional R-squared

```
> r.squaredGLMM(model_asv)
           R2m       R2c
[1,] 0.1810203 0.2092595
```

3.6 Plot model effects

```
plot(allEffects(model_asv))
```

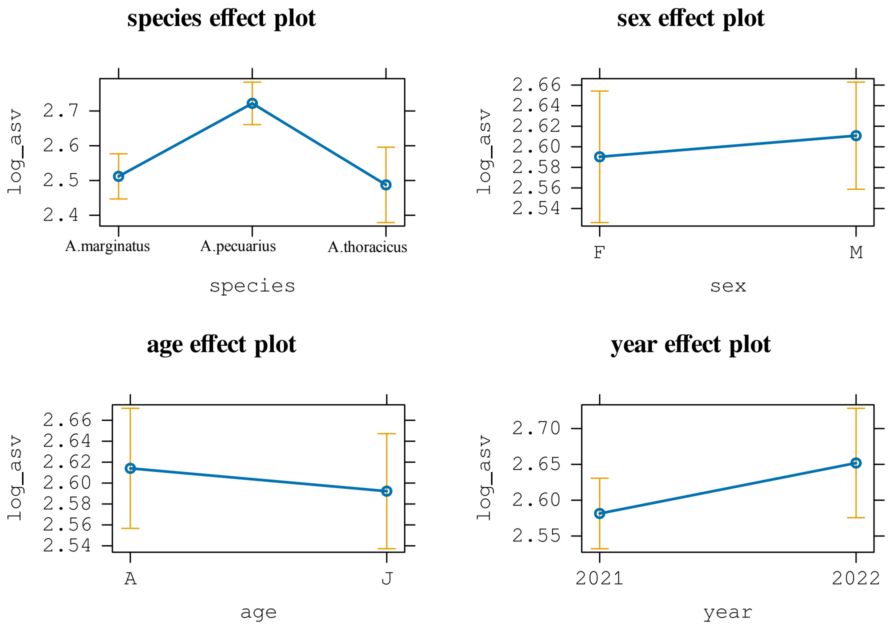

3.7 Significance of random effects

```
> ranova(model_asv1)
ANOVA-like table for random-effects: Single term deletions

Model:
log_asv ~ species + sex + age + year + (1 | nest)
           npar  logLik    AIC      LRT Df Pr(>Chisq)
<none>        8 -4.8628 25.726
(1 | nest)    7 -4.9126 23.825 0.099597  1     0.7523
```

B) Alpha diversity - adults

```
# Subset the dataset to include only adults
metadata_adults <- subset(metadata, age == "A")

# Plot data distribution
hist(metadata_adults$std_shannon)

# Check for outliers
check_outliers(metadata_adults$std_shannon)

OK: No outliers detected.
- Based on the following method and threshold: zscore_robust (3.291).
- For variable: metadata_adults$shannon_entropy
```

1. Model shannon diversity index

```
model_shannon_adults <- lmer(std_shannon ~ species + sex + year + (1|nest),data =
metadata_adults)
```

1.1 Check Normality

```
> check_normality(model_shannon_adults)
OK: residuals appear as normally distributed (p = 0.078).
```

1.2 Model Diagnostics

```
plot(check_distribution(model_shannon_adults))
```

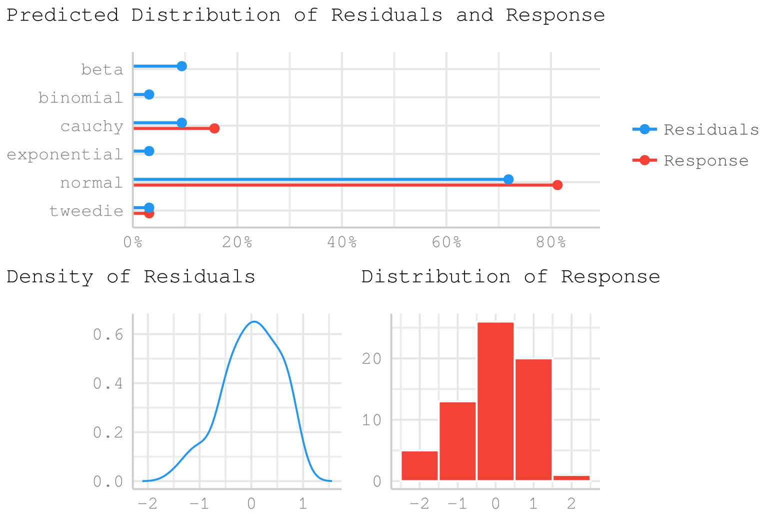

1.3 Model Summary

```
> summary(model_shannon_adults)

Linear mixed model fit by REML. t-tests use Satterthwaites method ['lmerModLmerTest']
Formula: std_shannon ~ species + sex + year + (1 | nest)
   Data: metadata_adults

REML criterion at convergence: 176.3

Scaled residuals:
     Min       1Q   Median       3Q      Max
-2.02368 -0.53319  0.04676  0.56535  1.26405

Random effects:
 Groups   Name        Variance Std.Dev.
 nest     (Intercept) 0.4035   0.6352
 Residual             0.5200   0.7211
Number of obs: 65, groups:  nest, 51

Fixed effects:
                   Estimate Std. Error       df t value Pr(>|t|)
(Intercept)         0.03557    0.27437 52.80645   0.130    0.897
speciesCpecuarius   0.17417    0.29863 45.79982   0.583    0.563
speciesCthoracicus -0.62381    0.39820 40.06428  -1.567    0.125
sexM               -0.09660    0.22840 31.90288  -0.423    0.675
year2022            0.36891    0.29154 41.38485   1.265    0.213
```

1.4 Bootstrap model

```
> boot_model_shannon_adults <- bootstrap(model_shanon_adults,.f = fixef, type = "parametric", B
= 10000, resample = c(TRUE, TRUE))

> summary(boot_model_shanon_adults)
Bootstrap type: parametric

Number of resamples: 10000

                term    observed    rep.mean        se          bias
1        (Intercept)  0.03556596  0.02966328 0.2770470 -0.0059026733
2  speciesApecuarius  0.17417214  0.17876448 0.3006861  0.0045923358
3 speciesAthoracicus -0.62381466 -0.61773817 0.3993916  0.0060764885
4               sexM -0.09659599 -0.09721253 0.2330595 -0.0006165440
5           year2022  0.36890665  0.36983417 0.2916459  0.0009275144

> confint(boot_model_shannon_adults,type = "norm")
# A tibble: 5 × 6
  term                estimate  lower upper type  level
  <chr>                  <dbl>  <dbl> <dbl> <chr> <dbl>
1 (Intercept)           0.0356 -0.502 0.584 norm   0.95
2 speciesApecuarius     0.174  -0.420 0.759 norm   0.95
3 speciesAthoracicus   -0.624  -1.41  0.153 norm   0.95
4 sexM                 -0.0966 -0.553 0.361 norm   0.95
5 year2022              0.369  -0.204 0.940 norm   0.95
```

```
# Note: In order to compute the confidence intervals for the remaining comparison (A.
thoracicus vs. A. pecuarius)change the reference level of the metadata to Athoracicus and
repeat the analysis.
metadata_adults$species <- relevel(metadata_adults$species, ref = "Athoracicus")

> confint(boot_model_shannon_adults, type = "norm") # with Athoracicus as reference level
# A tibble: 5 × 6
  term                 estimate   lower upper type  level
  <chr>                   <dbl>   <dbl> <dbl> <chr> <dbl>
1 (Intercept)           -0.588  -1.33   0.153 norm   0.95
2 speciesAmarginatus     0.624  -0.153  1.41  norm   0.95
3 speciesApecuarius      0.798   0.0389 1.56  norm   0.95
4 sexM                  -0.0966 -0.553  0.358 norm   0.95
5 year2022               0.369  -0.200  0.940 norm   0.95
```

1.5 Marginal and conditional R-squared

```
> r.squaredGLMM(model_shannon_adults)
            R2m       R2c
[1,] 0.09955472 0.492967
```

1.6 Plot model effects

```
plot(allEffects(model_shannon_adults))
```

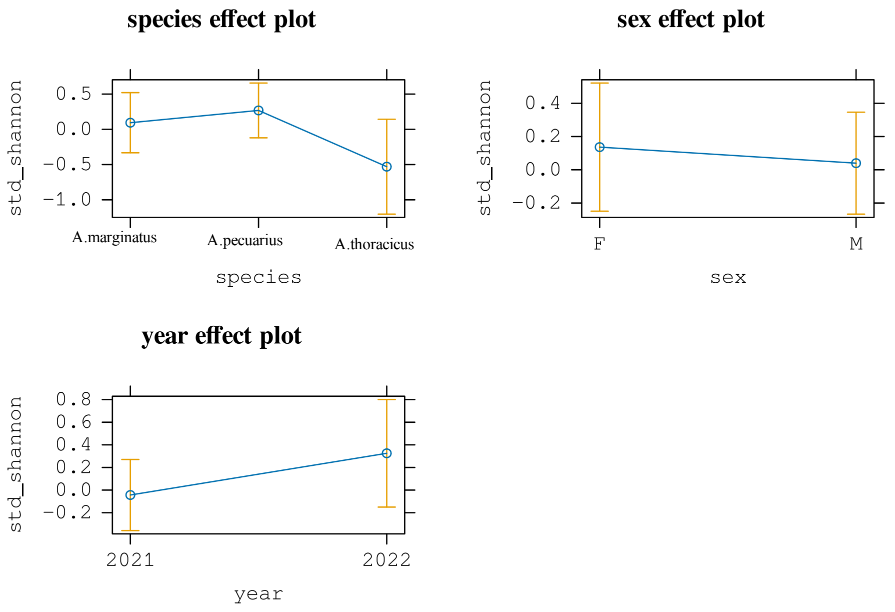

1.7 Significance of random effects

```
> ranova(model_shannon2)
ANOVA-like table for random-effects: Single term deletions

Model:
std_shannon ~ species + sex + year + (1 | nest)
           npar  logLik    AIC    LRT Df Pr(>Chisq)
<none>        7 -88.132 190.26
(1 | nest)    6 -89.424 190.85 2.5838  1      0.108
```

2. Model Faith phylogenetic diversity

```
# Plot data distribution
hist(metadata_adults$std_faith)

# Check for outliers
check_outliers(metadata_adults$std_faith)

OK: No outliers detected.
- Based on the following method and threshold: zscore_robust (3.291).
- For variable: metadata$faith_pd

# Model faith diversity
model_faith_adults <- lmer(std_faith ~ species + sex + year + (1|nest),data = metadata_juv)
```

2.1 Check Normality

```
> check_normality(model_faith_adults)
OK: residuals appear as normally distributed (p = 0.174).
```

2.2 Model Diagnostics

```
plot(check_distribution(model_faith_adults))
```

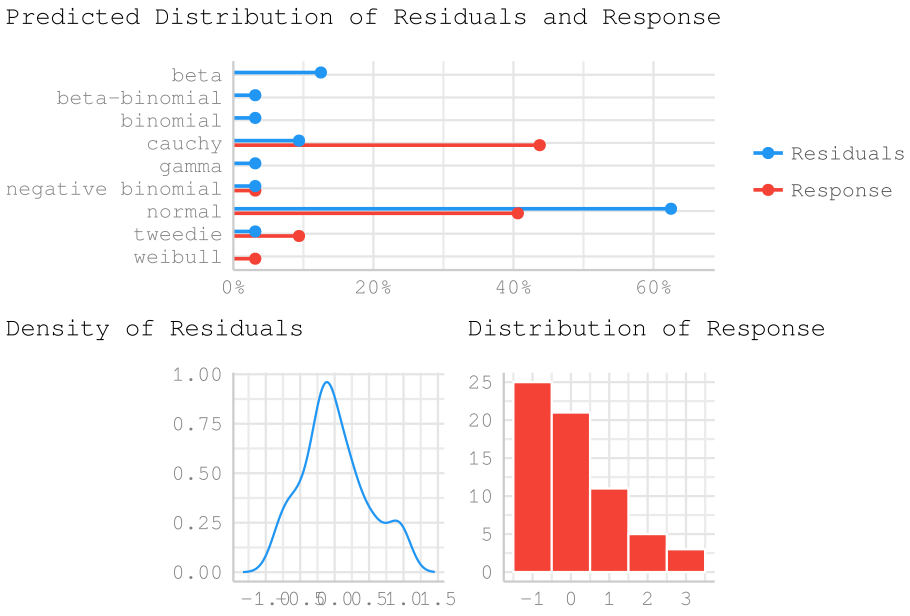

2.3 Model Summary

```
> summary(model_faith_adults)

Linear mixed model fit by REML. t-tests use Satterthwaites method ['lmerModLmerTest']
Formula: std_faith ~ species + sex + year + (1 | nest)
   Data: metadata_adults

REML criterion at convergence: 178.3

Scaled residuals:
    Min      1Q  Median      3Q     Max
-1.2878 -0.4381 -0.1200  0.3754  1.4717

Random effects:
 Groups   Name        Variance Std.Dev.
 nest     (Intercept) 0.5465   0.7392
 Residual             0.4435   0.6660
Number of obs: 65, groups:  nest, 51

Fixed effects:
                   Estimate Std. Error       df t value Pr(>|t|)
(Intercept)        -0.49156    0.28456 51.67143  -1.727  0.09006 .
species Apecuarius  0.91201    0.31270 44.33645   2.917  0.00553 **
species Athoracicus 0.05964    0.42002 38.88301   0.142  0.88782
sexM                0.04951    0.22031 24.63836   0.225  0.82405
year2022            0.26410    0.30700 40.05682   0.860  0.39476
---
Signif. codes:  0 '***' 0.001 '**' 0.01 '*' 0.05 '.' 0.1 ' ' 1
```

2.4 Bootstrap model

```
> boot_model_faith_adults <- bootstrap(model_faith_adults,.f = fixef, type = "parametric", B =
10000, resample = c(TRUE, TRUE))

> summary(boot_model_faith_adults)
```

```
Bootstrap type: parametric

Number of resamples: 10000

                 term    observed    rep.mean        se          bias
1         (Intercept) -0.49156345 -0.49326420 0.2832500 -0.0017007508
2  speciesApecuarius  0.91200984  0.91184165 0.3146867 -0.0001681944
3 speciesAthoracicus  0.05963960  0.06650180 0.4201834  0.0068621936
4                sexM  0.04950749  0.05177874 0.2266964  0.0022712448
5            year2022  0.26410425  0.25948522 0.3079346 -0.0046190333

> confint(boot_model_faith_adults, type = "norm")
# A tibble: 5 × 6
  term                estimate  lower  upper type  level
  <chr>                  <dbl>  <dbl>  <dbl> <chr> <dbl>
1 (Intercept)          -0.492  -1.05  0.0653 norm   0.95
2 speciesApecuarius     0.912   0.295 1.53   norm   0.95
3 speciesAthoracicus    0.0596 -0.771 0.876  norm   0.95
4 sexM                  0.0495 -0.397 0.492  norm   0.95
5 year2022              0.264  -0.335 0.872  norm   0.95

# Note: In order to compute the confidence intervals for the remaining comparison (A.
thoracicus vs. A. pecuarius)change the reference level of the metadata to Athoracicus and
repeat the analysis.
metadata_adults$species <- relevel(metadata_adults$species, ref = "Athoracicus")

> confint(boot_model_faith_adults, type = "norm") # with Athoracicus as reference level
# A tibble: 5 × 6
  term                estimate   lower upper type  level
  <chr>                  <dbl>   <dbl> <dbl> <chr> <dbl>
1 (Intercept)          -0.432  -1.20   0.329 norm   0.95
2 speciesAmarginatus  -0.0596 -0.884  0.767 norm   0.95
3 speciesApecuarius     0.852   0.0529 1.65  norm   0.95
4 sexM                  0.0495 -0.389  0.493 norm   0.95
5 year2022              0.264  -0.344  0.867 norm   0.95
```

2.5 Marginal and conditional R-squared

```
> r.squaredGLMM(model_faith_adults)
           R2m     R2c
[1,] 0.1663308  0.62651
```

2.6 Plot model effects

```
plot(allEffects(model_faith_adults))
```

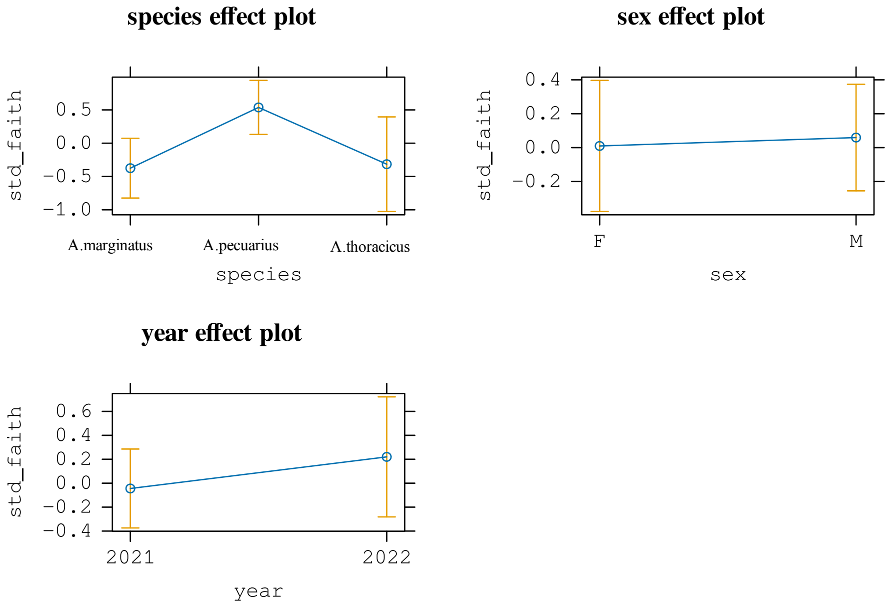

2.7 Significance of random effects

```
> ranova(model_faith_adults)

ANOVA-like table for random-effects: Single term deletions

Model:
std_faith ~ species + sex + year + (1 | nest)
            npar  logLik    AIC    LRT Df Pr(>Chisq)
<none>         7 -89.166 192.33
(1 | nest)     6 -90.710 193.42 3.0887  1    0.07884 .
---
Signif. codes:  0 '***' 0.001 '**' 0.01 '*' 0.05 '.' 0.1 ' ' 1
```

3. Model n° of observed ASV's

```
# Plot data distribution
hist(metadata_adults$std_asv)

# Check for outliers
check_outliers(metadata_adults$std_asv)

OK: No outliers detected.
- Based on the following method and threshold: zscore_robust (3.291).
- For variable: metadata$faith_pd

# Model n° of aboserved asv's
model_asv_adults <- lmer(std_asv ~ species + sex + year + (1|nest),data = metadata_juv)

# Check normality
> check_normality(model_asv_adults)
Warning: Non-normality of residuals detected (p = 0.044).

#Log transform rsponse variable
metadata_adults$log_asv <- log10(metadata_juv$observed_features)
hist(metadata_adults$log_asv)
```

```
check_distribution(metadata_adults$log_asv)

#Model transformed response variable
model_asv_adults <- lmer(log_asv ~ species + sex + year + (1|nest),data = metadata_adults)
```

3.1 Check Normality

```
> check_normality(model_asv_adults)
OK: residuals appear as normally distributed (p = 0.461).
```

3.2 Model Diagnostics

```
plot(check_distribution(model_asv_adults))
```

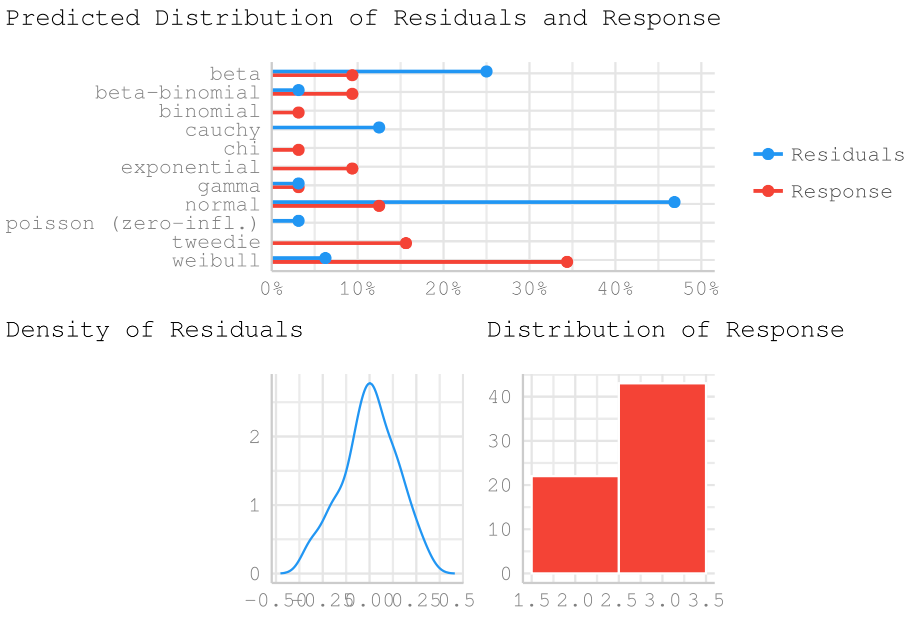

3.3 Model Summary

```
> summary(model_asv_adults)

Linear mixed model fit by REML. t-tests use Satterthwaites method ['lmerModLmerTest']
Formula: log_asv ~ species + sex + year + (1 | nest)
   Data: metadata_adults

REML criterion at convergence: 12.7

Scaled residuals:
     Min       1Q   Median       3Q      Max
-1.71035 -0.39138  0.01663  0.53705  1.60169

Random effects:
 Groups   Name        Variance Std.Dev.
 nest     (Intercept) 0.02432  0.1559
 Residual             0.03567  0.1889
Number of obs: 65, groups:  nest, 51
```

```
Fixed effects:
                     Estimate Std. Error       df t value Pr(>|t|)    
(Intercept)           2.46158    0.06986 49.56185  35.238   <2e-16 ***
speciesApecuarius     0.20454    0.07585 40.48504   2.697   0.0102 *  
speciesAthoracicus    0.06808    0.10094 33.68745   0.674   0.5046    
sexM                  0.01386    0.05914 26.80535   0.234   0.8165    
year2022              0.11758    0.07393 35.22181   1.590   0.1207    
---
Signif. codes:  0 '***' 0.001 '**' 0.01 '*' 0.05 '.' 0.1 ' ' 1
```

3.4 Bootstrap model

```
> boot_model_asv_adults <- bootstrap(model_asv_adults,.f = fixef, type = "parametric", B =
10000, resample = c(TRUE, TRUE))

> summary(boot_model_asv_adults)
Bootstrap type: parametric

Number of resamples: 10000

                term   observed   rep.mean         se          bias
1        (Intercept) 2.46158392 2.46192343 0.06979855  3.395140e-04
2  speciesCpecuarius 0.20453627 0.20489166 0.07587966  3.553913e-04
3 speciesCthoracicus 0.06807850 0.06693345 0.10050307 -1.145051e-03
4               sexM 0.01385836 0.01388420 0.06107758  2.583742e-05
5           year2022 0.11757560 0.11799396 0.07375176  4.183626e-04

> confint(boot_model_asv_adults, type = "norm")
# A tibble: 5 × 6
  term               estimate   lower upper type  level
  <chr>                 <dbl>   <dbl> <dbl> <chr> <dbl>
1 (Intercept)          2.46     2.32   2.60  norm   0.95
2 speciesApecuarius    0.205    0.0555 0.353 norm   0.95
3 speciesAthoracicus   0.0681 -0.128  0.266 norm   0.95
4 sexM                 0.0139 -0.106  0.134 norm   0.95
5 year2022             0.118  -0.0274 0.262 norm   0.95




# Note: In order to compute the confidence intervals for the remaining comparison (A.
thoracicus vs. A. pecuarius)change the reference level of the metadata to Athoracicus and
repeat the analysis.
metadata_adults$species <- relevel(metadata_adults$species, ref = "Athoracicus")

> confint(boot_model_asv_adults, type = "norm") # with Athoracicus as reference level
 A tibble: 5 × 6
  term               estimate   lower upper type  level
  <chr>                 <dbl>   <dbl> <dbl> <chr> <dbl>
1 (Intercept)          2.53     2.34   2.72  norm   0.95
2 speciesAmarginatus  -0.0681 -0.267  0.130 norm   0.95
3 speciesApecuarius    0.136  -0.0551 0.328 norm   0.95
4 sexM                 0.0139 -0.104  0.132 norm   0.95
5 year2022             0.118  -0.0293 0.264 norm   0.95
```

3.5 Marginal and conditional R-squared

```
> r.squaredGLMM(model_asv_adults)
           R2m       R2c
[1,] 0.142139 0.4899323
```

3.6 Plot model effects

```
plot(allEffects(model_asv_adults))
```

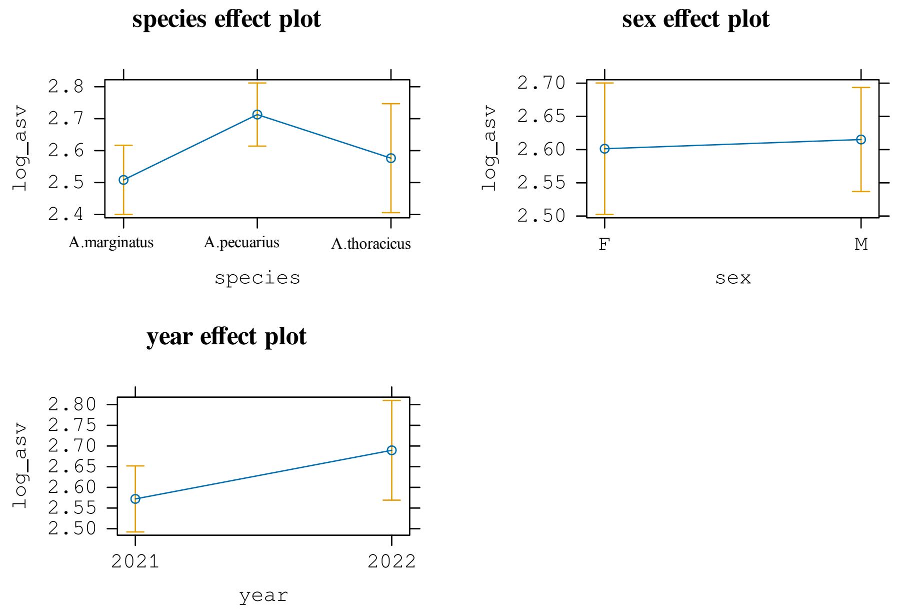

3.7 Significance of random effects

```
> ranova(model_asv_adults)

ANOVA-like table for random-effects: Single term deletions

Model:
log_asv ~ species + sex + year + (1 | nest)
           npar  logLik   AIC    LRT Df Pr(>Chisq)
<none>        7 -6.3401 26.68
(1 | nest)    6 -6.9649 25.93 1.2496  1     0.2636
```

C) Alpha diversity - juveniles

```
# Subset the dataset to include only adults
metadata_juv <- subset(metadata, age == "J")

# Plot data distribution
hist(metadata_juv$shannon_entropy)

# Check for outliers
check_outliers(metadata_juv$shannon_entropy)

OK: No outliers detected.
- Based on the following method and threshold: zscore_robust (3.291).
- For variable: metadata_juv$shannon_entropy
```

1. Model shannon diversity index

```
model_shannon_juv<- lmer(std_shannon ~ species + sex + year + (1|nest),data = metadata_juv)
```

1.1 Check Normality

```
> check_normality(model_shannon_juv)
OK: residuals appear as normally distributed (p = 0.064).
```

1.2 Model Diagnostics

```
plot(check_distribution(model_shannon_juv))
```

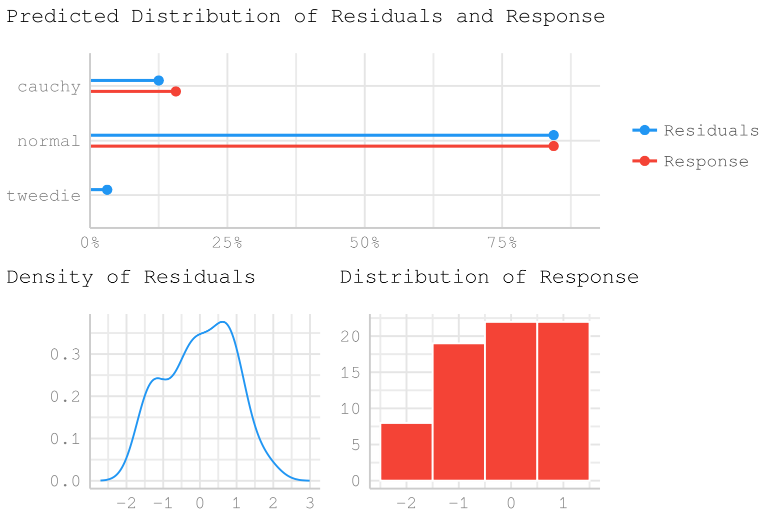

1.3 Model Summary

```
> summary(model_shannon_juv)

Linear mixed model fit by REML ['lmerMod']
Formula: std_shannon ~ species + sex + year + (1 | nest)
   Data: metadata_juv
```

```
REML criterion at convergence: 195.9

Scaled residuals:
     Min       1Q   Median       3Q      Max
-1.72906 -0.67909  0.00956  0.73845  2.02522

Random effects:
 Groups   Name        Variance Std.Dev.
 nest     (Intercept) 0.006963 0.08344
 Residual             0.908452 0.95313
Number of obs: 71, groups:  nest, 52

Fixed effects:
                    Estimate Std. Error t value
(Intercept)          -0.3325     0.2583  -1.287
speciesCpecuarius     0.5951     0.2523   2.359
speciesCthoracicus   -0.5960     0.3748  -1.590
sexM                  0.1721     0.2331   0.738
year2022             -0.1546     0.2683  -0.576
```

1.4 Bootstrap model

```
> boot_model_shannon_juv <- bootstrap(model_shannon_juv,.f = fixef, type = "parametric", B =
10000, resample = c(TRUE, TRUE))

> summary(boot_model_shannon_juv)
Bootstrap type: parametric

Number of resamples: 10000

                term   observed   rep.mean        se          bias
1        (Intercept) -0.3324814 -0.3377723 0.2598437 -0.005290867
2  speciesApecuarius  0.5951313  0.5986337 0.2523863  0.003502433
3 speciesAthoracicus -0.5960196 -0.5868362 0.3773204  0.009183431
4               sexM  0.1720963  0.1736686 0.2349709  0.001572333
5           year2022 -0.1546445 -0.1474506 0.2673873  0.007193882

> confint(boot_model_shannon_juv,type = "norm")
# A tibble: 5 × 6
  term                estimate   lower upper type  level
  <chr>                  <dbl>   <dbl> <dbl> <chr> <dbl>
1 (Intercept)           -0.332 -0.836  0.182 norm   0.95
2 speciesApecuarius      0.595  0.0970 1.09  norm   0.95
3 speciesAthoracicus    -0.596 -1.34   0.134 norm   0.95
4 sexM                   0.172 -0.290  0.631 norm   0.95
5 year2022              -0.155 -0.686  0.362 norm   0.95


# Note: In order to compute the confidence intervals for the remaining comparison (A.
thoracicus vs. A. pecuarius)change the reference level of the metadata to Athoracicus and
repeat the analysis.
metadata_juv$species <- relevel(metadata_juv$species, ref = "Athoracicus")

> confint(boot_model_shannon_juv, type = "norm") # with Athoracicus as reference level
# A tibble: 5 × 6
  term                estimate  lower  upper type  level
  <chr>                  <dbl>  <dbl>  <dbl> <chr> <dbl>
```

```
1 (Intercept)           -0.929 -1.62  -0.232 norm    0.95
2 speciesAmarginatus     0.596 -0.153  1.33  norm    0.95
3 speciesApecuarius      1.19   0.464  1.90  norm    0.95
4 sexM                   0.172 -0.289  0.639 norm    0.95
5 year2022              -0.155 -0.678  0.373 norm    0.95
```

1.5 Marginal and conditional R-squared

```
> r.squaredGLMM(model_shannon_juv)
           R2m       R2c
[1,] 0.1719504 0.1782489
```

1.6 Plot model effects

```
plot(allEffects(model_shannon_juv))
```

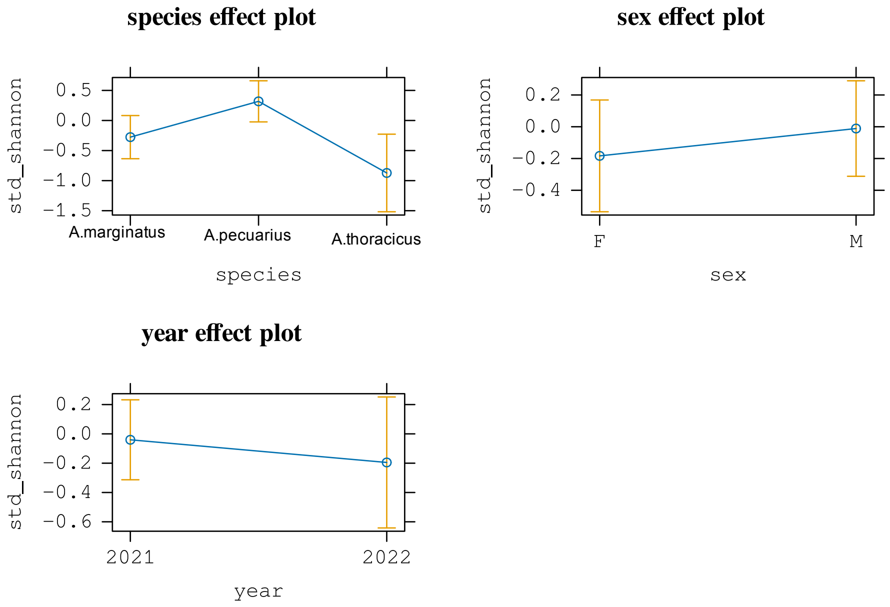

1.7 Significance of random effects

```
> ranova(model_shannon_juv)
ANOVA-like table for random-effects: Single term deletions

Model:
std_shannon ~ species + sex + year + (1 | nest)
           npar  logLik    AIC        LRT Df Pr(>Chisq)
<none>        7 -97.955 209.91
(1 | nest)    6 -97.956 207.91 0.00056251  1     0.9811
```

2. Model Faith phylogenetic diversity

```
# Plot data distribution
```

```
hist(metadata_juv$faith_pd)

# Check for outliers
check_outliers(metadata_juv$faith_pd)

OK: No outliers detected.
- Based on the following method and threshold: zscore_robust (3.291).
- For variable: metadata_juv$faith_pd

# Model faith diversity
model_faith_juv <- lmer(std_faith ~ species + sex + year + (1|nest),data = metadata_juv)

#Check normality
> check_normality(model_faith_juv)
Warning: Non-normality of residuals detected (p = 0.043).

#Transform faith pd
metadata_juv$sqrt_faith <- sqrt(metadata_juv$faith_pd)

# Model transformed faith diversity
model_faith_juv <- lmer(sqrt_faith ~ species + sex + year + (1|nest),data = metadata_juv)
```

2.1 Check Normality

```
> check_normality(model_faith_juv)
OK: residuals appear as normally distributed (p = 0.174).
```

2.2 Model Diagnostics

```
plot(check_distribution(model_faith_juv))
```

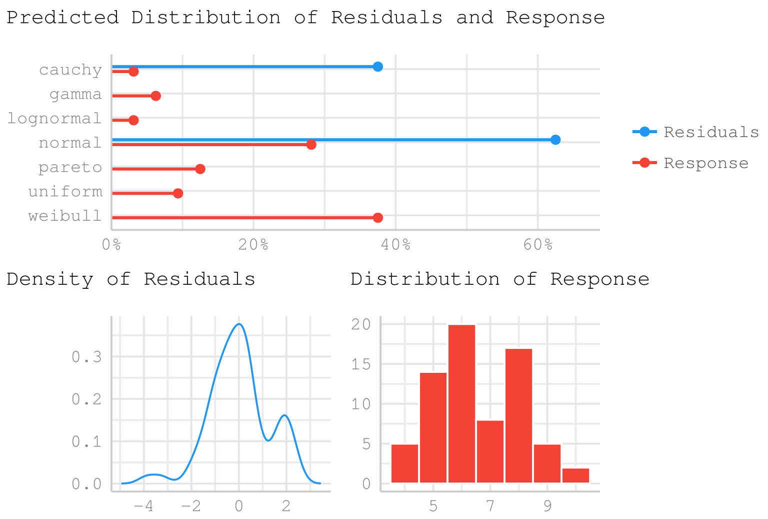

2.3 Model Summary

```
> summary(model_faith_juv)

Linear mixed model fit by REML. t-tests use Satterthwaites method ['lmerModLmerTest']
Formula: sqrt_faith ~ species + sex + year + (1 | nest)
   Data: metadata_juv

REML criterion at convergence: 235.5

Scaled residuals:
     Min       1Q   Median       3Q      Max
-3.01163 -0.53725 -0.02478  0.41600  1.85350

Random effects:
 Groups   Name        Variance Std.Dev.
 nest     (Intercept) 0.000    0.000
 Residual             1.668    1.291
Number of obs: 71, groups:  nest, 52

Fixed effects:
                   Estimate Std. Error      df t value Pr(>|t|)
(Intercept)          5.8960     0.3480 66.0000  16.942  < 2e-16 ***
speciesApecuarius    1.3298     0.3397 66.0000   3.914 0.000217 ***
speciesAthoracicus  -0.8460     0.5046 66.0000  -1.677 0.098355 .
sexM                 0.2281     0.3147 66.0000   0.725 0.471161
year2022             0.1761     0.3613 66.0000   0.487 0.627682
---
Signif. codes:  0 '***' 0.001 '**' 0.01 '*' 0.05 '.' 0.1 ' ' 1
```

2.4 Bootstrap model

```
> boot_model_faith_juv <- bootstrap(model_faith_juv,.f = fixef, type = "parametric", B = 10000,
resample = c(TRUE, TRUE))

> summary(boot_model_faith_juv)
Bootstrap type: parametric

Number of resamples: 10000

                term   observed   rep.mean         se         bias
1        (Intercept)  5.8959861  5.8916458 0.3496435 -0.004340260
2  speciesApecuarius  1.3297537  1.3347146 0.3386427  0.004960981
3 speciesAthoracicus -0.8459644 -0.8435346 0.5046154  0.002429789
4               sexM  0.2280968  0.2311806 0.3208265  0.003083860
5           year2022  0.1760648  0.1772531 0.3637967  0.001188301

> confint(boot_model_juv, type = "norm")
# A tibble: 5 × 6
  term               estimate  lower upper type  level
  <chr>                 <dbl>  <dbl> <dbl> <chr> <dbl>
1 (Intercept)           5.90   5.20  6.59  norm   0.95
2 speciesApecuarius     1.33   0.663 2.00  norm   0.95
3 speciesAthoracicus   -0.846 -1.85  0.164 norm   0.95
4 sexM                  0.228 -0.397 0.856 norm   0.95
5 year2022              0.176 -0.521 0.885 norm   0.95
```

```
# Note: In order to compute the confidence intervals for the remaining comparison (A.
thoracicus vs. A. pecuarius)change the reference level of the metadata to Athoracicus and
repeat the analysis.
metadata_juv$species <- relevel(metadata_juv$species, ref = "Athoracicus")

> confint(boot_model_faith_juv, type = "norm") # with Athoracicus as reference level
# A tibble: 5 × 6
  term                 estimate  lower upper type  level
  <chr>                   <dbl>  <dbl> <dbl> <chr> <dbl>
1 (Intercept)             5.05   4.12  5.97  norm   0.95
2 speciesAmarginatus      0.846 -0.139 1.83  norm   0.95
3 speciesApecuarius       2.18   1.22  3.13  norm   0.95
4 sexM                    0.228 -0.394 0.846 norm   0.95
5 year2022                0.176 -0.519 0.886 norm   0.95
```

2.5 Marginal and conditional R-squared

```
> r.squaredGLMM(model_faith_juv)
           R2m       R2c
[1,] 0.2844682 0.2844682
```

2.6 Plot model effects

```
plot(allEffects(model_faith_juv))
```

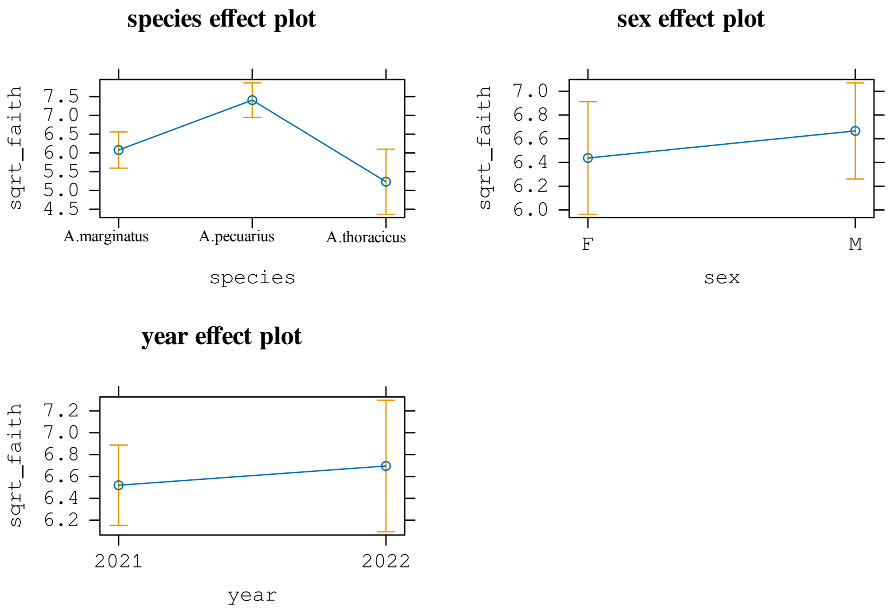

2.7 Significance of random effects

```
> ranova(model_faith_juv)
ANOVA-like table for random-effects: Single term deletions

Model:
sqrt_faith ~ species + sex + year + (1 | nest)
           npar  logLik    AIC   LRT  Df  Pr(>Chisq)
<none>        7 -117.76 249.52
(1 | nest)    6 -117.76 247.52     0   1          1
```

3. Model n° of observed ASV's

```
# Plot data distribution
hist(metadata_juv$std_asv)

# Check for outliers
check_outliers(metadata_juv$std_asv)

OK: No outliers detected.
- Based on the following method and threshold: zscore_robust (3.291).
- For variable: metadata_juv$std_asv

# Model n° of aboserved asv's
model_asv_juv <- lmer(std_asv ~ species + sex + year + (1|nest),data = metadata_juv)

# Check normality
> check_normality(model_asv_juv)
Warning: Non-normality of residuals detected (p = 0.048).

#Log transform rsponse variable
metadata_juv$log_asv <- log10(metadata_juv$observed_features)
hist(metadata_juv$log_asv)
check_distribution(metadata_juv$log_asv)

#Model transformed response variable
model_asv_juv <- lmer(log_asv ~ species + sex + year + (1|nest),data = metadata_juv)
```

3.1 Check Normality

```
> check_normality(model_asv_juv)
OK: residuals appear as normally distributed (p = 0.121).
```

3.2 Model Diagnostics

```
plot(check_distribution(model_asv_juv))
```

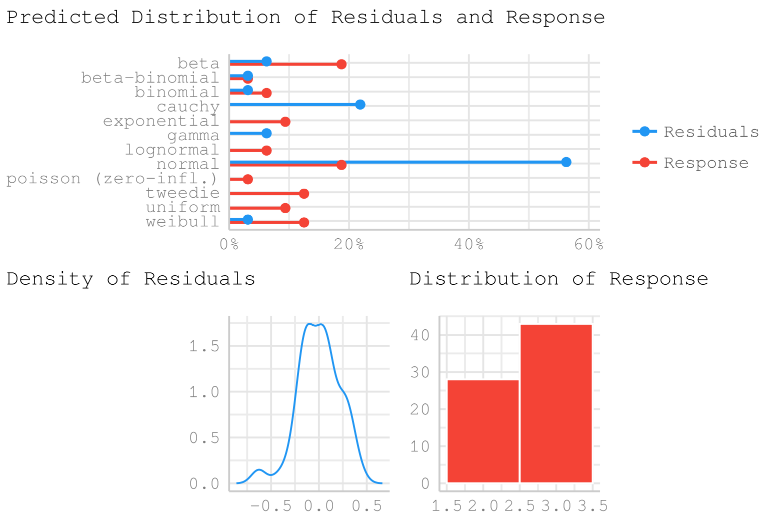

3.3 Model Summary

```
> summary(model_asv_juv)

Linear mixed model fit by REML. t-tests use Satterthwaites method ['lmerModLmerTest']
Formula: log_asv ~ species + sex + year + (1 | nest)
   Data: metadata_juv

REML criterion at convergence: 3.5

Scaled residuals:
     Min       1Q   Median       3Q      Max
-2.83836 -0.64045  0.04173  0.56186  1.94654

Random effects:
 Groups   Name        Variance Std.Dev.
 nest     (Intercept) 0.00000  0.0000
 Residual             0.04958  0.2227
Number of obs: 71, groups:  nest, 52

Fixed effects:
                   Estimate Std. Error       df t value Pr(>|t|)
(Intercept)         2.48407    0.06000 66.00000  41.399  < 2e-16 ***
speciesCpecuarius   0.21979    0.05858 66.00000   3.752 0.000372 ***
speciesCthoracicus -0.12625    0.08700 66.00000  -1.451 0.151445
sexM                0.02501    0.05426 66.00000   0.461 0.646358
year2022            0.03192    0.06230 66.00000   0.512 0.610115
---
Signif. codes:  0 ‘***’ 0.001 ‘**’ 0.01 ‘*’ 0.05 ‘.’ 0.1 ‘ ’ 1
```

3.4 Bootstrap model

```
> boot_model_asv_juv <- bootstrap(model_asv_juv,.f = fixef, type = "parametric", B = 10000,
resample = c(TRUE, TRUE))

> summary(boot_model_asv_juv)
Bootstrap type: parametric

Number of resamples: 10000
```

```
                  term    observed    rep.mean         se          bias
1         (Intercept)  2.48407253  2.48320190 0.06135174 -8.706282e-04
2  speciesCpecuarius  0.21979134  0.22095195 0.05904554  1.160614e-03
3 speciesCthoracicus -0.12625366 -0.12505219 0.08749058  1.201468e-03
4                sexM  0.02501157  0.02495783 0.05503979 -5.373506e-05
5           year2022  0.03191863  0.03286223 0.06257820  9.435987e-04

> confint(boot_model_asv_juv, type = "norm")
# A tibble: 5 × 6
  term                estimate   lower  upper type  level
  <chr>                  <dbl>   <dbl>  <dbl> <chr> <dbl>
1 (Intercept)           2.48     2.36   2.61   norm   0.95
2 speciesApecuarius     0.220    0.103  0.334  norm   0.95
3 speciesAthoracicus   -0.126   -0.299  0.0440 norm   0.95
4 sexM                  0.0250 -0.0828 0.133  norm   0.95
5 year2022              0.0319 -0.0917 0.154  norm   0.95


# Note: In order to compute the confidence intervals for the remaining comparison (A.
thoracicus vs. A. pecuarius)change the reference level of the metadata to Athoracicus and
repeat the analysis.
metadata_juv$species <- relevel(metadata_juv$species, ref = "Athoracicus")

> confint(boot_model_asv_juv, type = "norm") # with Athoracicus as reference level
# A tibble: 5 × 6
  term                estimate   lower upper type  level
  <chr>                  <dbl>   <dbl> <dbl> <chr> <dbl>
1 (Intercept)           2.36     2.20   2.52  norm   0.95
2 speciesCmarginatus    0.126   -0.0450 0.298 norm   0.95
3 speciesCpecuarius     0.346    0.181  0.512 norm   0.95
4 sexM                  0.0250 -0.0826 0.132 norm   0.95
5 year2022              0.0319 -0.0900 0.154 norm   0.95
```

3.5 Marginal and conditional R-squared

```
> r.squaredGLMM(model_asv_juv)
           R2m       R2c
[1,] 0.256622 0.256622
```

3.6 Plot model effects

```
plot(allEffects(model_asv_juv))
```

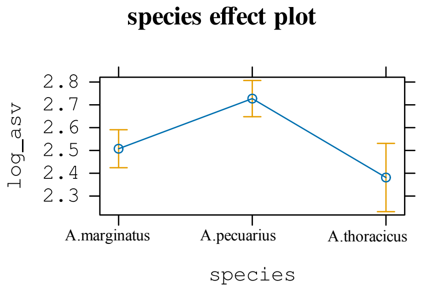

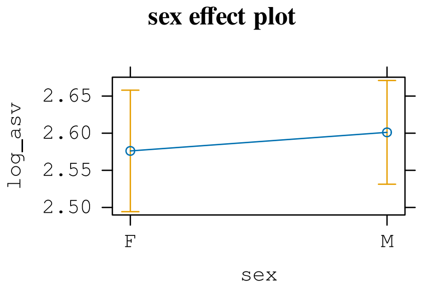

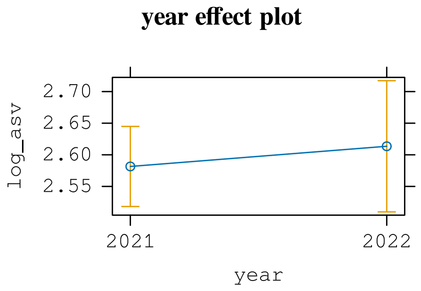

3.7 Significance of random effects

```
> ranova(model_asv_juv)

ANOVA-like table for random-effects: Single term deletions

Model:
log_asv ~ species + sex + year + (1 | nest)
           npar  logLik    AIC      LRT      Df Pr(>Chisq)
<none>        7 -1.7412 17.482
(1 | nest)    6 -1.7412 15.482    5.862e-14  1          1
```
