# Supplementary material for: The gut microbiota of three avian species living in sympatry
Source: BMC Ecol Evol. 2024 Nov 21;24:144. doi: 10.1186/s12862-024-02329-9 (PMC11580620; doi:10.1186/s12862-024-02329-9)
Supplement: Supplementary file 6 — Additional file 6. Appendix F. Differential abundance analysis. [file 12862_2024_2329_MOESM6_ESM.pdf]

Differential Abundant Analysis

Differential Abundant Analysis
1. Read in the data
2. Differential abundant analysis - combined dataset
2.1 Differential abundant taxa between species
3. Differential abundant analysis - Adults
3.1 Differential abundant taxa between years
4. Differential abundance analysis - Juveniles
4.1 Differential abundant taxa between species
4.2 Plot differential abundant taxa

1. Read in the data

```
# Load libraries
library(Maaslin2)
library(phyloseq)
library(qiime2R)
library(microbiome)
library(tidyverse)
library(ggplot2)

# Create phyloseq object
ps <- qza_to_phyloseq(
  features="filtered-table-final.qza",
  tree="rooted-tree.qza",
  taxonomy = "taxonomy.qza",
  metadata = "plover_meta_alpha.tsv")

#remove individuals with more than one sampling point
duplicates <- duplicated(sample_data(ps)$ring_number)
ps <- prune_samples(!duplicates, ps)

#Filter ps object, remove samples with no sex assignment, remove samples from nests with only
one individual.
# remove samples with no sex
ps <- subset_samples(ps, !is.na(sample_data(ps)$sex) & sample_data(ps)$sex != "")#remove
samples with no sex assignement

#remove samples from nests with only one individual
duplicate_nest <- duplicated(sample_data(ps)$nest) | duplicated(sample_data(ps)$nest, fromLast
= TRUE)
ps <- subset_samples(ps, duplicate_nest)

# Remove samples with zero counts
ps <- prune_samples(!zero_counts_samples, ps)
ps <- prune_taxa(taxa_sums(otu_table(ps)) > 1, ps) # remove singletons

summarize_phyloseq(ps)
```

2. Differential abundant analysis - combined dataset

```
# Split data by age.
ps <- readRDS("ps.rds")
ps_genus <- ps %>% tax_glom("Genus") # agglomerate at genus level

metadata <- data.frame(sample_data(ps))
asv_table_genus <- data.frame(otu_table(ps_genus))
asv_table_genus <- t (asv_table_genus)
asv_table_genus <- data.frame(asv_table_genus)

# Run Maaslin2

results_all1 <- Maaslin2(input_data      = asv_table_genus,
                            input_metadata = metadata,
                            normalization   = "CSS",
                            transform = "LOG",
                            analysis_method = "LM",
                            max_significance = 0.05,
                            output          = "genus_all_pecuarius",
                            fixed_effects   = c("species", "sex", "age", "year"),
                            reference       = c("species,Cpecuarius") #pecuarius as referecence
                            random_effects = c("nest"),
                            correction = "holm",
                            plot_heatmap = TRUE,
                            min_prevalence = 0.1,
                            min_abundance   = 0.0001)

results_all2 <- Maaslin2(input_data      = asv_table_genus,
                            input_metadata = metadata,
                            normalization   = "CSS",
                            transform = "LOG",
                            analysis_method = "LM",
                            max_significance = 0.05,
                            output          = "genus_all_marginatus",
                            fixed_effects   = c("species", "sex", "age", "year"),
                            reference       = c("species,Cmarginatus"), #marginatus as reference
in order to have all pairwise comparisons
                            random_effects = c("nest"),
                            correction = "holm",
                            plot_heatmap = TRUE,
                            min_prevalence = 0.1,
                            min_abundance   = 0.0001)
```

2.1 Differential abundant taxa between species

Comparison	Kingdom	Phylum	Class	Order	Family	Genus	Coefficient	Std.Err	N	N.not.0	pval	qval
**Athoracicus-Apecuarius**	Bacteria	Firmicutes	Clostridia	Peptostreptococcales-Tissierellales	Anaerovoracaceae	Eubacterium brachy	5.22	0.81	136	75	2.10E-08	5.66E-05
**Athoracicus-Apecuarius**	Bacteria	Firmicutes	Clostridia	Lachnospirales	Lachnospiraceae	CHKCI001	4.17	0.74	136	79	1.06E-07	2.87E-04
**Athoracicus-Apecuarius**	Bacteria	Bacteroidota	Bacteroidia	Bacteroidales	Marinifilaceae	Odoribacter	-3.58	0.64	136	98	6.78E-07	1.83E-03
**Athoracicus-Apecuarius**	Bacteria	Firmicutes	Clostridia	Oscillospirales	Ruminococcaceae	Paludicola	5.55	1.13	136	103	2.66E-06	7.17E-03

Comparison	Kingdom	Phylum	Class	Order	Family	Genus	Coefficient	Std.Err	N	N.not.0	pval	qval
**Athoracicus-Apecuarius**	Bacteria	Firmicutes	Clostridia	Oscillospirales	Ruminococcaceae	Negativibacillus	4.63	0.95	136	90	8.81E-06	2.37E-02
**Athoracicus-Apecuarius**	Firmicutes	Clostridia	Clostridia vadinBB60	Clostridia vadinBB60	Clostridia vadinBB60	Clostridia vadinBB60	4.25	0.96	136	109	1.84E-05	4.94E-02
**Athoracicus-Amarginatus**	Bacteria	Firmicutes	Clostridia	Lachnospirales	Lachnospiraceae	CHKCI001	4.42	0.76	136	79	3.92E-08	1.06E-04
**Athoracicus-Amarginatus**	Bacteria	Actinobacteriota	Actinobacteria	Frankiales	Geodermatophilaceae	Geodermatophilus	1.32	0.28	136	14	5.69E-06	1.54E-02
**Athoracicus-Amarginatus**	Bacteria	Proteobacteria	Alphaproteobacteria	Rhizobiales	Beijerinckiaceae	Microvirga	4.32	0.87	136	69	1.71E-05	4.61E-02
**Amarginatus-Apecuarius**	Bacteria	Proteobacteria	Alphaproteobacteria	Rhizobiales	Rhizobiales_Incertae_Sedis	Nordella	-1.21	0.26	136	25	1.12E-05	3.02E-02
**Amarginatus-Apecuarius**	Bacteria	Firmicutes	Negativicutes	Veillonellales-Selenomonadales	Veillonellaceae	Megasphaera	-2.60	0.52	136	65	1.13E-05	3.06E-02
**Juveniles-Adults**	Bacteria	Proteobacteria	Gammaproteobacteria	Enterobacterales	Succinivibrionaceae	Anaerobiospirillum	-2.61	0.51	136	104	1.74E-06	4.70E-03

3. Differential abundant analysis - Adults

```
# Split data by age.
ps <- readRDS("ps.rds")
ps_adults <- subset_samples(ps, age == "A") # split by age

ps_genus_adults <- ps_adults %>% tax_glom("Genus") # agglomerate at genus level
taxonomy_adults <- data.frame(tax_table(ps_adults))
taxonomy_adults$ASV <- row.names(taxonomy_adults)

table_genus_adults <- data.frame(otu_table(ps_genus_adults))
table_genus_adults <- t (table_genus_adults)
table_genus_adults <- data.frame(table_genus_adults)

# Run Maaslin2

results_genus_adults1 <- Maaslin2(input_data     = table_genus_adults,
                            input_metadata = metadata_adults,
                            normalization  = "CSS",
                            transform = "LOG",
                            analysis_method = "LM",
                            max_significance = 0.05,
                            output         = "genus_adults_marginatus",
                            fixed_effects  = c("species", "sex", "year"),
                            reference      = c("species,Cmarginatus"), #marginatus as
referecence
                            random_effects = c("nest"),
                            correction = "holm",
                            min_prevalence = 0.1,
                            min_abundance  = 0.0001)

results_genus_adults2 <- Maaslin2(input_data     = table_genus_adults,
                            input_metadata = metadata_adults,
                            normalization  = "CSS",
                            transform = "LOG",
                            analysis_method = "LM",
                            max_significance = 0.05,
                            output         = "genus_adults_pecuarius",
                            fixed_effects  = c("species", "sex", "year"),
```

```
                             reference      = c("species,Cpecuarius"), #pecuarius as reference
in order to have all pairwise comparisons
                             random_effects = c("nest"),
                             correction = "holm",
                             min_prevalence = 0.1,
                             min_abundance  = 0.0001)

# No DAA taxa detected between species
#Detected one diferential abundant taxa between years
```

3.1 Differential abundant taxa between years

Comparison	Kingdom	Phylum	Class	Order	Family	Genus	Coeficient	Std.err	N	N.not.0	pval	qval
2021 vs. 2022	*Bacteria*	*Firmicutes*	*Bacilli*	*Erysipelotrichales*	*Erysipelotrichaceae*	*Ileibacterium*	2.067182196	0.416582761	65	21	1.34E-05	0.030157687

4. Differential abundance analysis - Juveniles

```
# Split data by age.)
ps_juv <- subset_samples(ps, age == "J") # split by age

ps_genus_juv <- ps_juv %>% tax_glom("Genus") # agglomerate at genus level
taxonomy_juv <- data.frame(tax_table(ps_juv))
taxonomy_juv$ASV <- row.names(taxonomy_juv)

table_genus_juv <- data.frame(otu_table(ps_genus_juv))
table_genus_juv <- t (table_genus_juv)
table_genus_juv <- data.frame(table_genus_juv)

# Run Maaslin2

results_genus_juv1 <- Maaslin2(input_data     = table_genus_juv,
                             input_metadata = metadata_juv,
                             normalization  = "CSS",
                             transform = "LOG",
                             analysis_method = "LM",
                             max_significance = 0.05,
                             output         = "genus_juv_marginatus",
                             fixed_effects  = c("species", "sex", "year"),
                             reference      = c("species,Cmarginatus"), #marginatus as
referecence
                             random_effects = c("nest"),
                             correction = "holm",
                             min_prevalence = 0.1,
                             min_abundance  = 0.0001)

results_genus_juv2 <- Maaslin2(input_data     = table_genus_juv,
                             input_metadata = metadata_juv,
                             normalization  = "CSS",
                             transform = "LOG",
                             analysis_method = "LM",
                             max_significance = 0.05,
                             output         = "genus_juv_pecuarius",
                             fixed_effects  = c("species", "sex", "year"),
                             reference      = c("species,Cpecuarius"), #pecuarius as reference
in order to have all pairwise comparisons
```

```
                              random_effects = c("nest"),
                              correction = "holm",
                              min_prevalence = 0.1,
                              min_abundance  = 0.0001)



# Get taxonomy of the DAA taxa

diferential_genus_juv <- c("eed573c05495947419cf90d591c1dd2c",
"9c564fcc88c51566a00b856c243b30e6", "aa1a1593d17d547e0d71c07b7b7d4aed",
"10d231ec069b6e07f8661d855adbcd90", "89f2da9aa12ce5c3236c99b85535907d",
"1b68aabc541b47fc2c5c297be4e1f572")

DAA_genus_juv_ps <- prune_taxa(taxa_names(ps_genus_juv) %in% diferential_genus_juv,
ps_genus_juv)
DAA_genus_juv_ps@tax_table

Taxonomy Table:     [6 taxa by 7 taxonomic ranks]:
                                       Kingdom       Phylum         Class          Order
                        Family
eed573c05495947419cf90d591c1dd2c "d__Bacteria" "Bacteroidota" "Bacteroidia" "Bacteroidales"
                  "Marinifilaceae"
10d231ec069b6e07f8661d855adbcd90 "d__Bacteria" "Firmicutes"   "Bacilli"     "Lactobacillales"
                  "Lactobacillaceae"
aa1a1593d17d547e0d71c07b7b7d4aed "d__Bacteria" "Firmicutes"   "Bacilli"     "Lactobacillales"
                  "Lactobacillaceae"
1b68aabc541b47fc2c5c297be4e1f572 "d__Bacteria" "Firmicutes"   "Clostridia"
 "Peptostreptococcales-Tissierellales" "Anaerovoracaceae"
89f2da9aa12ce5c3236c99b85535907d "d__Bacteria" "Firmicutes"   "Clostridia"  "Lachnospirales"
                   "Lachnospiraceae"
9c564fcc88c51566a00b856c243b30e6 "d__Bacteria" "Firmicutes"   "Clostridia"  "Lachnospirales"
                   "Lachnospiraceae"
                                 Genus                          Species
eed573c05495947419cf90d591c1dd2c "Odoribacter"                  NA
10d231ec069b6e07f8661d855adbcd90 "Lactobacillus"                NA
aa1a1593d17d547e0d71c07b7b7d4aed "Limosilactobacillus"          NA
1b68aabc541b47fc2c5c297be4e1f572 "[Eubacterium]_brachy_group"   NA
89f2da9aa12ce5c3236c99b85535907d "CHKCI001"                     NA
9c564fcc88c51566a00b856c243b30e6 "Lachnospiraceae_NK4A136_group" NA
```

4.1 Differential abundant taxa between species

Comparison	Kingdom	Phylum	Class	Order	Family	Genus	Coefficient	Std.Err	N	N.not.0	pval	qval
Athoracicus vs. Apecuarius	Bacteria	Bacteroidota	Bacteroidia	Bacteroidales	Marinifilaceae	Odoribacter	-4.249959199	0.823241341	71	53	2.43E-06	0.004954326
Athoracicus vs. Apecuarius	Bacteria	Firmicutes	Clostridia	Lachnospirales	Lachnospiraceae	Lachnospiraceae_NK4A136_group	-4.119498848	0.849350475	71	59	7.86E-06	0.016020002
Athoracicus vs. Apecuarius	Bacteria	Firmicutes	Bacilli	Lactobacillales	Lactobacillaceae	Limosilactobacillus	-3.844588121	0.803858545	71	55	1.01E-05	0.020559331
Athoracicus vs. Apecuarius	Bacteria	Firmicutes	Bacilli	Lactobacillales	Lactobacillaceae	Lactobacillus	-2.286990754	0.483105178	71	70	1.21E-05	0.024590252
Athoracicus vs. Apecuarius	Bacteria	Firmicutes	Clostridia	Lachnospirales	Lachnospiraceae	CHKCI001	6.114523026	1.275053397	71	37	2.26E-05	0.046104227
Athoracicus vs. Apecuarius	Bacteria	Firmicutes	Clostridia	Peptostreptococcales-Tissierellales	Anaerovoracaceae	[Eubacterium]_brachy_group	4.648127117	1.019500118	71	36	2.28E-05	0.046459465

4.2 Plot differential abundant taxa

```r
daa_df <- read.table("daa_results.tsv", header = TRUE, sep = "\t")

# Create the plot
ggplot(daa_df, aes(x = coef, y = Genus, fill = ifelse(coef > 0, "positive", "negative"))) +
  geom_bar(stat = "identity", color = "black") +
  geom_vline(xintercept = 0, color = "black") +  # Add vertical line at 0
  labs(x = "Coefficient", y = "Genus") +  # Label axes
  theme_classic() +  # Use minimal theme
  guides(fill = FALSE) +   # Remove the legend
  scale_fill_manual(values = c("positive" = "blue", "negative" = "red"))+   # Set fill colors
  theme(axis.text.x = element_text(size = 14, family = "Arial"),
        axis.text.y = element_text(size = 14, family = "Arial"),
        axis.title.x = element_text(size = 14, family = "Arial"),
        axis.title.y = element_text(size = 14, family = "Arial"))
```
